
JOURNAL INFORMATION
==============================
NLM Title Abbreviation: Spine Deform
Journal ID: Spine Deform
NLM Journal ID: 101603979

Spine Deformity

ISSN: 2212-134X
EISSN: 2212-1358

ARTICLE INFORMATION
==============================
PMCID: PMC7661559
PMID: 33185856
DOI: 10.1007/s43390-020-00234-x
Article ID: 234
Article version: 1
Subjects: Abstracts

14th International Congress on Early Onset Scoliosis

November 14, 2020

Electronic publication date: 2020 Nov 13
Print publication date: 2020
Volume: 8
Issue: 6
First page: 1389
Last page: 1422
Copyright: © The Author(s) 2020
License: Open AccessThis article is licensed under a Creative Commons Attribution 4.0 International License, which permits use, sharing, adaptation, distribution and reproduction in any medium or format, as long as you give appropriate credit to the original author(s) and the source, provide a link to the Creative Commons licence, and indicate if changes were made. The images or other third party material in this article are included in the article's Creative Commons licence, unless indicated otherwise in a credit line to the material. If material is not included in the article's Creative Commons licence and your intended use is not permitted by statutory regulation or exceeds the permitted use, you will need to obtain permission directly from the copyright holder. To view a copy of this licence, visit http://creativecommons.org/licenses/by/4.0/.
License URL: https://creativecommons.org/licenses/by/4.0/

issue-copyright-statement: © Scoliosis Research Society 2020

==============================

### Paper # 1.

## Rib-based anchors do not impair chest wall motion in early onset scoliosis

*Yubing Tong, Jayaram K. Udupa, Joseph M. McDonough, Caiyun Wu, Catherine Qiu, Carina Lott, MS, Nirupa Galagedera, Jason B. Anari, MD, Drew A. Torigian, Patrick J. Cahill, MD*

**Summary**: Currently, there are no quantitative approaches to evaluate the motion of the chest wall after surgical intervention. Quantitative dynamic magnetic resonance imaging (QdMRI), a novel methodology, can quantify the relative contribution of chest wall motion. Analyses of 30 early onset scoliosis patients (EOS) before and after surgery demonstrate that rib-based fixation does not decrease the volumetric contribution of the chest wall to the respiratory cycle volume.

**Hypothesis**: Rib-based fixation in EOS patients will decrease chest wall contribution to respiratory volumes.

**Introduction**: There is concern that rib-based fixation limits chest wall motion. We propose evaluating the contribution of chest wall excursion to respiration before and after surgical intervention using a novel methodology, QdMRI.

**Methods**: Dynamic MR images acquired from 30 EOS patients (pre- and post-surgical) and 51 normal children were evaluated. Tidal volumetric parameters for chest walls and hemi-diaphragms via QdMRI based on segmentation results from 4D constructed images were analyzed for concave and convex sides of the spinal curve. Differential functional responses in thoracic components (concave and convex, separately) were compared before and after surgery.

**Results**: Tidal volumes of chest wall and diaphragm components increased after surgery, with 116.69% and 51.04% tidal volume increase for hemi-diaphragm ($p < .001$) and chest wall ($p = .009$) on concave side, and 60.91% and 12.66% tidal volume increase for hemi-diaphragm ($p = .004$) and chest wall ($p = .41$) on convex side. Tidal volume ratios of diaphragm/chest wall (Dtv/CWtv) are closer to the ratio for normal subjects for concave ($p = .68$) and convex ($p = .95$) sides after surgery than before surgery ($p = .01$ for both concave and convex sides). No significant difference was found in the ratio between concave and convex sides before surgery ($p = .97$) or after surgery ($p = .75$).

**Conclusion**: Chest wall and diaphragm component tidal volumes significantly improve after rib-based fixation on both concave and convex sides. The improvement is significantly greater on the concave side than the convex side. Rib-based surgery appears to normalize the relative contribution of the chest wall to diaphragm in the respiratory cycle.

**Table 1.** Diaphragm and chest wall component tidal volumes (mean ± SD) and their ratios (mean ± SD) before and after surgery shown separately for concave and convex sides. Volume ratio $r = (Volume_{post\text{-}surgical} - Volume_{pre\text{-}surgical}) / Volume_{pre\text{-}surgical} \times 100\%$

| | Concave (Dtv) | Convex (Dtv) | Concave (CWtv) | Convex (CWtv) | Concave (Dtv/CWtv) | Convex (Dtv/CWtv) | Normal (Dtv/CWtv) | P value (Concave, Normal) | P value (Convex, Normal) | P value (Concave, Convex) |
|---|---|---|---|---|---|---|---|---|---|---|
| Pre-surgical | 11.80 ± 10.98 | 12.96± 9.37 | 15.07± 11.47 | 16.61± 10.70 | 1.00 ± 0.62 | 1.01 ± 0.55 | 1.41 ± 0.76 | **.01** | **.01** | .97 |
| Post-surgical | 25.56 ± 21.65 | 19.58± 11.42 | 24.25 ± 16.19 | 18.72± 10.06 | 1.31 ± 1.03 | 1.39 ± 0.92 | | .68 | .95 | .75 |
| r (%) | 116.69 | 51.04 | 61.09 | 12.66 | 30.76 | 38.0 | | - | - | - |
| P value | **< .001** | **.009** | **.004** | .41 | .25 | **.01** | | - | - | - |

CWtv: chest wall tidal volume, left or right, separately (in ml)
Dtv: hemi-diaphragm tidal volume, left or right, separately (in ml)

*Author Affiliations and Disclosures: Jason B. Anari, MD, Children's Hospital of Philadelphia; Patrick J. Cahill, MD, The Children's Hospital of Philadelphia; NuVasive Inc (Consultant), Children's Spine Foundation (Grants/Research), Setting Scoliosis Straight Foundation (Grants/Research); Nirupa Galagedera, Children's Hospital of Philadelphia; Carina Lott, MS, Children's Hospital of Philadelphia; Joseph M. McDonough, Children's Hospital of Philadelphia; Catherine Qiu, The Children's Hospital of Philadelphia; Yubing Tong, Department of Radiology, University of Pennsylvania; Drew A. Torigian, Department of Radiology, University of Pennsylvania; Jayaram K. Udupa, Department of Radiology, University of Pennsylvania; Caiyun Wu, Department of Radiology, University of Pennsylvania*

### Paper # 2.

## Surgeon distraction forces in open lengthenings exceed MCGR maximums: an in vivo biomechanical study

*Vincent Ruggeri, Benjamin Sinder, PhD, Catherine Qiu, Carina Lott, MS, Patrick J. Cahill, MD, Jason B. Anari, MD*

**Summary**: Surgeon distraction forces vary by construct type and frequently exceed MCGR maximums.

**Hypothesis**: Growing construct distraction force can be accurately measured in vivo and will vary by factors such as construct type.

**Introduction**: Existing manual distraction techniques benefit from utilizing surgeon "feel" and experience in determining the force applied. While newer technologies such as magnetically controlled growing rods (MCGR) provide the benefit of non-invasive distraction, the force is not controlled. Future remote lengthening technologies may allow for selection of specific distraction forces, but knowledge of what force to apply is not well characterized and may vary by factors such as construct type. Therefore, the purpose of this study was to quantify the force applied by surgeons in vivo to determine appropriate baseline values for distraction force and assess differences by construct type.

**Methods**: A distraction device was outfitted with an electronic strain gauge and calibrated to accurately measure distraction force. After validation in a cadaver lab, IRB approval was attained to measure distraction force in vivo. Peak distraction force is reported. A one-way ANOVA followed by t-tests was used.

**Results**: A total of 26 patients were measured, ranging in age from 3 to 14, with 15 males and 11 females. 38 distraction force measurements were made: 18 rib to pelvis, 12 rib to rib and 8 rib to spine. In rib to rib constructs (336 ± 143 N) we found surgeons used 58% more force ($p < 0.01$) than in rib to pelvis constructs (212 ± 100 N). Similarly, surgeons used 70% greater force ($p < 0.01$) for rib to spine (362 ± 133 N) constructs compared with rib to pelvis. Across all construct types, 74% (28/38) of distractions used a peak force exceeding 187 N, the reported maximum possible force with MCGR technology.

**Conclusion**: Distraction force varies by construct type and exceeds MCGR maximums.

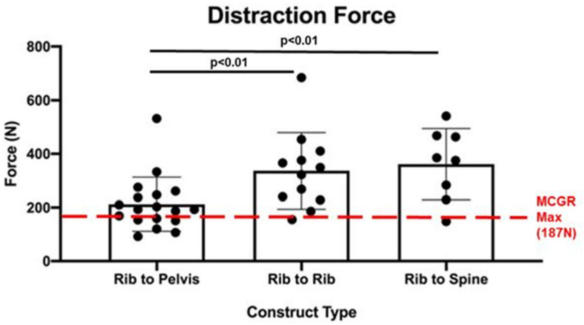

**Figure:** 38 *in vivo* distraction force measurements shows less force is used in rib to pelvis constructs and 74% of all distractions exceeded the magnetically controlled growing rod (MCGR) maximum force of 187N.

*Author Affiliations and Disclosures: Jason B. Anari, MD, Children's Hospital of Philadelphia; Patrick J. Cahill, MD, The Children's Hospital of Philadelphia; NuVasive Inc (Consultant), Children's Spine Foundation (Grants/Research), Setting Scoliosis Straight Foundation (Grants/Research); Carina Lott, MS, Children's Hospital of Philadelphia; Catherine Qiu, The Children's Hospital of Philadelphia; Vincent Ruggeri, Children's Hospital of Philadelphia; Benjamin Sinder, PhD, Children's Hospital of Philadelphia*

### Paper # 3.

### The effect of apex vertebra position on growing rod treatment

*Muharrem Yazici, MD, Gőkay Dursun, Riza Mert Cetik, MD, Gokhan Demirkiran, Mehmet Ayvaz*

**Summary**: The purpose of GR treatment is to preserve spinal growth at a near-normal level while effectively controlling deformity. In index surgery, bringing the apex in line with longitudinal members (rods) provides more effective growth preservation and rotational deformity control.

**Hypothesis**: Bringing apical vertebral segments to midline with GR application positively affects growth preservation and deformity control.

**Introduction**: GR aims to preserve growth and control deformity by the longitudinal distraction of the spine. The size and flexibility of the deformity and the degree of correction achieved in index surgery affect the success of the technique.

**Methods**: Between 2000 and 19, 140 patients treated with GR were evaluated. Exclusion criteria: < 2-year FU, a vertebral anomaly at apical segments. Deformity characteristics (in the coronal/sagittal planes) and length of T1-12, T1-S1, instrumented segments were measured on pre- and post-index and final FU X-rays. The degree of apical rotation (Nash-Moe) was measured. The patients were divided into 3 groups according to the apex rotation on the post-index X-ray: Gr 1(both pedicles are located between 2 rods), Gr 2(concave rod located between 2 pedicles), Gr 3(concave pedicle located lateral to the convex rod) (Fig. 1).

**Results**: Fifty-seven patients (36F, 21 M) fulfilled criteria (mean age at index surgery 7.3 (3.2–12.2)). Forty-five patients were managed with TGR, 12 with MCGR. According to the rotational position of the apical vertebra; 10 patients (17.5%) were in Gr 1, 33 (57.9%) in Gr 2, and 14 (24.6%) in Gr 3. Radiologic results were summarized in Fig. 2. Longitudinal growth was significantly higher in Gr1 for every parameter measured. While there was no difference between the groups in terms of coronal and sagittal plane deformity corrections, rotational deformity increase was significantly higher in Gr 3.

**Conclusion**: Bringing the apex in line with GR in index surgery increases the capacity of growth preservation and rotational control in GR. If the apex cannot be brought to the midline with simple distraction, apical control strategies should be considered (preop HGT; concave rib osteotomies or apical control with screw/cable as adjunct procedure).

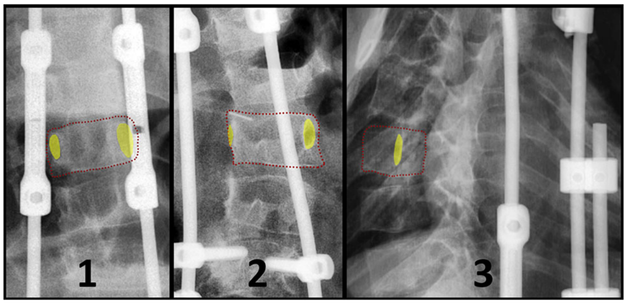

| | Group 1 | Group 2 | Group 3 | p value |
|---|---|---|---|---|
| **Duration of lengthening (years)** | 7.5 (4.2-10) | 5.8 (2.1-12) | 4.3 (2-9.6) | 0.207 |
| **# of instrumented segments** | 13 (12-15) | 13 (11-16) | 14 (12-17) | 0.290 |
| **T1-T12 height gain (cm/year)** | 0.62 (0.42-0.93) | 0.52 (0.18-1.23) | 0.36 (0.17-0.56) | **<0.001** |
| **T1-S1 height gain (cm/year)** | 1.02 (0.7-1.4) | 0.84 (0.43-1.22) | 0.62 (0.35-0.92) | **<0.001** |
| **Height change per each instrumented segment (cm/year)** | 0.057 (0.016-0.093) | 0.043 (0.011-0.090) | 0.035 (0.009-0.049) | **=0.003** |
| **Coronal plane correction Pre-index vs Post-index (%)** | 50.2 (22.9-84.4) | 47.5 (9.4- 80.2) | 44.7 (4.9-62.9) | =0.282 |
| **Coronal plane correction Pre-index vs Final (%)** | 35.2 (0-87.7) | 36.2 (-2 – 81.8) | 25.4 (-1.8 – 51.2) | =0.296 |
| **Apical vertebra rotation Pre-index vs Final** | p=0.261 | p=0.172 | p=0.846 | |
| **Apical vertebra rotation Post-index vs Final** | p=0.223 | p=0.435 | **p=0.050** | |

*Author Affiliations and Disclosures: Mehmet Ayvaz, Kayacan Group; Riza Mert Cetik, MD, Hacettepe University Faculty of Medicine; Gokhan Demirkiran, Hacettepe University Medical Faculty; Gőkay Dursun, Hacettepe University Orthopaedics and Traumatology Department; Muharrem Yazici, MD, Hacettepe University*

### Paper # 4.

### Predicting unplanned return to the OR with MCGR

*Matthew Oetgen, MD MBA, John T. Smith, MD, Michael G. Vitale, MD MPH, John Heflin, MD, John M. Flynn, MD*

**Summary**: A predictive model for the risk of UPROR after treatment with MCGR for idiopathic and congenital EOS is possible. Patients with long segment kyphosis, short pre-operative trunks, and maximal initial correction of the deformity are at higher risk for UPROR following implantation with MCGR.

**Hypothesis**: A model to predict the probability of UPROR for MCGR can be developed.

**Introduction**: As MCGR has become ubiquitous to treat EOS patient outcomes have demonstrated higher than expected complications, particularly unplanned return to OR (UPROR). A better understanding of patient selection is needed to optimize use of MCGR and minimize UPROR. We investigated patient and surgical characteristics of patients treated with MCGR attempting to develop a predictive model of UPROR.

**Methods**: A prospective multicenter EOS database was queried to identify patients treated with primary MCGR for EOS with minimum 2-year follow-up. The group was analyzed based on etiology of scoliosis: Group 1: Idiopathic, congenital. Group 2: neuromuscular, syndromic.

**Results**: 272 patients treated with MCGR for EOS, 57% female; etiologies: 7% congenital, 26% idiopathic, 39% neuromuscular, 22% syndromic, 6% other. 44% of the patients had at least one complication at 2 years follow up, with 78 patients (28%) having UPROR. For the Group 1, a predictive model of UPROR was developed with the following predictors: gender, pre-op kyphosis length (# vertebra levels), pre-op T1-S1 length (cm), immediate post-op T1-T12 and T1-S1 length(cm). The predictive ability of this model was tested with area under the curve analysis, with an AUC of 0.84: excellent model discrimination. An equation to estimate the probability of UPROR was developed (Fig. 1). Data was insufficient for a predictive model for Group 2 patients.

**Conclusion**: MCGR has become widely adopted as a treatment for EOS, but complication rates remain high. Understanding patients at higher risk for UPROR may assist in treatment planning and family education. In the idiopathic and congenital cohort, patients with long segment kyphosis, short pre-operative trunks, and maximal initial correction of the deformity are at higher risk for UPROR following implantation with MCGR.

Table 1. Significant predictors of UPROR for idiopathic and congenital patients

| Predictor | β (SE) | $e^{\beta}$ (Odds Ratio) | p-value |
|---|---|---|---|
| Constant 1 (α) | 3.401 (2.217) | Not necessary | Not necessary |
| Gender (0=Male, 1=Female) | -0.861 (0.555) | 0.42 | 0.12 |
| **Pre index kyphosis length** | 0.273 (0.141) | 1.31 | **0.05** |
| **Pre index T1-S1** | -0.018 (0.007) | 0.98 | **0.006** |
| **Post implant T1-T12** | -0.05 (0.019) | 0.95 | **0.008** |
| **Post implant T1-S1** | 0.025 (0.014) | 1.03 | **0.08** |

Equation for prediction of Probability of UPROAR=

$$\frac{e^{(3.401 - 0.861 \cdot \text{Gender} + 0.273 \cdot \text{Pre index kyphosis length} - 0.018 \cdot \text{Pre index T1-S1} - 0.05 \cdot \text{Post implant T1-T12} + 0.025 \cdot \text{Post implant T1-S1})}}{1 + e^{(3.401 - 0.861 \cdot \text{Gender} + 0.273 \cdot \text{Pre index kyphosis length} - 0.018 \cdot \text{Pre index T1-S1} - 0.05 \cdot \text{Post implant T1-T12} + 0.025 \cdot \text{Post implant T1-S1})}}$$

*Author Affiliations and Disclosures: John M. Flynn, MD, Children's Hospital of Philadelphia, Biomet (Consultant); John Heflin, MD, University of Utah; Matthew Oetgen, MD MBA, Children's National Health System; John T. Smith, MD, University of Utah; Michael G. Vitale, MD MPH, Columbia University Medical Center/Morgan Stanley Children's Hospital*

### Paper # 5.

### Actuator end cap separation in magnetically controlled growing rods (MCGR)

*Ying Li, MD, Tricia St. Hilaire, MPH, John T. Anderson, Mark Erickson, MD, MMM, Amy McIntosh, MD, Peter F. Sturm, MD, David L. Skaggs, MD, Michael G. Vitale, MD MPH, Ron El-Hawary, MD, Pediatric Spine Study Group*

**Summary**: Actuator end cap separation in MCGR can be visualized on an AP spine radiograph but also on ultrasound in select cases. The affected rods continued to lengthen and the scoliosis remained well-controlled. A serum titanium (Ti) level was checked in only one patient and was significantly elevated.

**Hypothesis**: MCGR lengthening can continue successfully after end cap separation but patients may have elevated serum Ti levels.

**Introduction**: MCGR are a popular growth friendly technique to control early onset scoliosis (EOS). In February 2020, a class II FDA recall was issued for the newest generation of MCGR due to separation of the actuator end cap. While the rod may continue to lengthen, separation of the end cap may expose the inside of the actuator, potentially leading to degeneration of the internal components and extravasation of Ti alloy wear debris. The purpose of this study was to review MCGR patients with reported end cap separation from an EOS database.

**Methods**: Children treated with the newest generation of MCGR with reported end cap separation were identified.

**Results**: Six patients were identified. End cap separation occurred after initial MCGR insertion in 4 patients and after MCGR revision in 2 patients. Demographic, radiographic, and surgical data are shown in Table 1. Complete radiographic data was available for 4 patients. Details of the end cap separation were available for 5 patients (Table 2). End cap separation occurred at mean 0.7 years (0.6–0.9 years) after rod insertion and was noted in only one rod for each patient. The rod on the concave side of the major curve was most commonly affected. Rod lengthening continued successfully (Fig. 1) and the spinal deformity remained well-controlled. No clinical symptoms were noted and none of the rods were exchanged. One patient had serum Ti levels checked. She had a pre-index serum Ti level < 1 ng/mL (normal) that increased to 26 ng/mL after end cap separation was found 0.6 years after rod insertion.

**Conclusion**: Patients with MCGR should be closely monitored for end cap separation with serial imaging. The rod continues to lengthen so prophylactic rod exchange may not be necessary. However, serum Ti levels may be elevated and warrant observation despite unclear significance.

**Table 1.** Demographic, radiographic, and surgical data for patients who experienced MCGR end cap separation.

| *Demographics* | N=6 |
|---|---|
| Age at index surgery [(years; mean (range)] | 6.5 (4.8-10.1) |
| Sex, male [n (%)]) | 3 (50) |
| Etiology of scoliosis (n) | |
| Idiopathic | 1 |
| Congenital | 2 |
| Neuromuscular (muscular dystrophy) | 1 |
| Syndromic (Beckwith-Weidemann, Soto's) | 2 |
| BMI at index surgery [kg/m²; mean (range)] | 17.3 (13.3-20.6) |
| Ambulatory status at index surgery (n) | |
| Ambulatory | 4 |
| Nonambulatory | 2 |
| Duration of follow-up [(years; mean (range)] | 1.2 (0.6-3.0) |
| | |
| *Radiographic data* | N=4 |
| Pre-index major curve [°; mean (range)] | 54 (32-78) |
| Pre-index maximum kyphosis [°; mean (range)] | 27 (0-46) |
| Post-index major curve [°; mean (range)] | 46 (16-65) |
| Post-index maximum kyphosis [°; mean (range)] | 45 (12-87) |
| Most recent major curve [°; mean (range)] | 49 (23-67) |
| Most recent maximum kyphosis [°; mean (range)] | 52 (8-94) |
| | |
| *Surgical data* | N=6 |
| Rod construct (n) | |
| Standard/offset | 3 |
| Standard/standard | 2 |
| Offset/offset | 1 |
| Length of construct (n) | |
| T3-L3 | 1 |
| T3-L4 | 3 |
| T3-pelvis | 1 |
| T4-pelvis | 1 |
| Length of actuator with end cap separation | |
| 70 mm | 1 |
| 90 mm | 5 |
| Diameter of rod with end cap separation | |
| 4.5 mm | 2 |
| 5.0 mm | 2 |
| 5.5 mm | 1 |
| 6.0 mm | 1 |

BMI, body mass index

**Table 2.** End cap separation data.

| | N=5 |
|---|---|
| Time from MCGR insertion to end cap separation [years; mean (range)] | 0.7 (0.6-0.9) |
| Diagnostic imaging modality (n) | |
| AP spine radiograph | 3 |
| AP spine radiograph + ultrasound | 2 |
| Rod affected (n) | |
| Standard rod on concave side of major curve | 3 |
| Offset rod on concave side of major curve | 1 |
| Standard rod on convex side of major curve | 1 |
| Number of rod lengthenings prior to end cap separation (range) | 1-2 |
| Number of rod lengthenings after end cap separation (range) | 1-2 |

AP, anteroposterior

Figure 1. End cap separation (shown by the circles) in right MCGR noted on AP spine radiograph and ultrasound. Note the appearance of the intact left rod pre- and post-lengthening and the right rod pre- and post-lengthening on ultrasound.

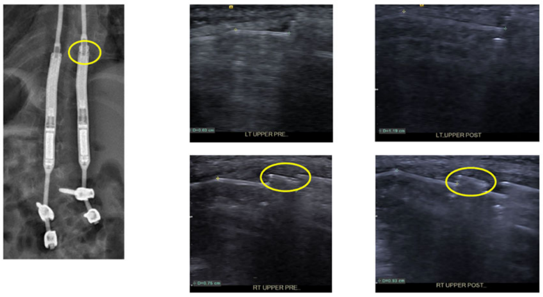

*Author Affiliations and Disclosures: John T. Anderson, Children's Mercy Kansas City; Ron El-Hawary, MD, IWK Health Centre, Depuy Synthes (Grants/Research), Medtronic (Grants/Research), Orthopediatrics (Consultant, Advisory Board or Panel, Stock/Shareholder (self-managed)); Mark Erickson, MD, MMM, Children's Hospital Colorado; Ying Li, MD, C. S. Mott Children's Hospital, Michigan Medicine, Scoliosis Research Society (Grants/Research), Medtronic, Inc. (Advisory Board or Panel); Amy McIntosh, MD, Texas Scottish Rite Hospital for Children, Nuvasive (Consultant); David L. Skaggs, MD, Children's Hospital of Los Angeles; Tricia St. Hilaire, MPH, Children's Spine Foundation; Pediatric Spine Study Group, Children's Spine Foundation; Peter F. Sturm, MD, Cincinnati Children's Hospital Medical Center; Michael G. Vitale, MD MPH, Columbia University Medical Center/Morgan Stanley Children's Hospital; Pediatric Spine Study Group, Pediatric Orthopaedic Society of North America (Grants/Research), FDA (Grants/Research), NuVasive (Grants/Research)*

### Paper # 6.

### Perioperative serious adverse events after instrumented cervical spine fusion in young children

*Maxwell H. McKee-Proctor, Bram Verhofste, John B. Emans, MD, M. Timothy Hresko, MD, Mark R. Proctor, MD, Daniel Hedequist, MD*

**Summary**: This is the largest single-center study of 57 children < 8 years old which evaluated acute 90-day perioperative serious adverse events (SAE) after cervical spine instrumentation and fusion (CSIF). We found that seven patients (12%) developed SAE after CSIF, with grade III SAE being the most frequent. Increased SAE were found in patients with greater number of comorbidities and those undergoing occipitocervical fusion.

**Hypothesis**: Is instrumented cervical spine fusion safe in young children?

**Introduction**: Limited data exists on SAE in young children undergoing CSIF. The purpose of this study was to examine acute perioperative SAE after CSIF in young children < 8 years.

**Methods**: 57 children (mean 5.8 ± 2.3 y; range 0.2–8.9 y) who underwent modern CSIF at a single institution were analyzed (2003–2019). The modified Clavien–Dindo-Sink classification was used to assess the severity of complications based on the required intervention and outcome after SAE. Grades III-V were considered SAE. Statistical analysis compared patients with and without SAE.

**Results**: Seven patients (12%) sustained SAE following CSIF: 6 grade III (11%) and 1 grade IVa (2%). There was 1 intraoperative SAE. The remaining 6 postoperative SAE occurred after a mean of 23 days postoperative (range 4–74 days). SAE included 1 respiratory (2%), 1 implant (2%), 2 wound (4%), 2 halo (4%), and 1 vascular (2%) complications. Children with and without SAE had similar surgical/anesthesia durations, ICU days, and hospital admissions. However, 2 patients (4%) developed SAE after discharge and required readmission. Children who developed SAE had increased comorbidities (5.0 vs 2.3; p = 0.03) and underwent occipitocervical fusion more often (86% vs 28%; p = 0.003).

**Conclusion**: In this large series of 57 children < 8-years old, 7 children (12%) sustained an SAE in the 90-day perioperative period after CSIF. Grade III SAE were the most frequent. The presence of occipitocervical fusion and number of comorbidities were risk factors for SAE. However, more research is necessary to develop risk stratification scores to identify high-risk patients and prevent SAE.

**Table 1. Cohort summary**

| Patient demographics | Mean | ± SD | Range | P value |
|---|---|---|---|---|
| Age at surgery (years) | 5.8 | ± 2.3 | 0.2 – 8.9 | 0.69 |
| Sex (freq; % male) | 29 | 51% | / | 0.65 |
| ≥1 comorbidity (freq; %) | 43 | 75% | / | / |
| Pre-op neurologic deficit | 28 | 49% | / | 0.65 |

| Operative details | Mean | ± SD | Range | P value |
|---|---|---|---|---|
| ASA classification (N=55; N; %) | | | | 0.31 |
| ASA class 1 + 2 | 17 | 31% | / | |
| ASA class 3 + 4 | 38 | 69% | / | |
| Occipitocervical fusion (N; %) | 20 | 35% | / | 0.003 |
| Estimated blood loss (mL) | 115 | ± 163 | 0 – 1000 | 0.39 |
| Anesthesia duration (mins) | 290 | ± 99 | 151 – 686 | 0.11 |
| Surgical duration (mins) | 231.1 | ± 96 | 50 – 672 | 0.82 |
| Bone morphogenic protein (N; %) | 19 | 33% | / | 0.57 |
| Intubation ≥1 day | 19 | 33% | / | 0.78 |
| ICU admission (days) | 3.1 | ± 6.8 | 0 – 39 | 0.95 |

| Serious adverse events | | Etiology | Indication | CSIF | Type | Grade | Treatment |
|---|---|---|---|---|---|---|---|
| Case 1 | 8yo F | Pleiomorphic astrocytoma | Post-laminectomy kyphosis | cAP O-C7 | Wound: Deep SSI | 3 | I&D VAC x7 |
| Case 2 | 6yo M | T21 | Non-union w/ BI | PSF O-C3 | Wound: Seroma/hematoma | 3 | I&D Drain X3 |
| Case 3 | 4yo F | Intramedullary AVM | Post-laminectomy kyphosis | PSF C2-C5 | Halo: Pin-site infection | 3 | Pin revision |
| Case 4 | 6yo M | Klippel-Feil | Cervical scoliosis | PSF O-C3 | Halo: Pin-site loosening | 3 | Pin revision |
| Case 5 | 7yo F | T21 | Os odontoideum w/ C1-2 instability | PSF O-C2 | Respiratory: Atelectasis/aspiration pneumonitis | 4A | Intubation + ICU readmission, tracheotomy |
| Case 6 | 5yo F | T21 | OC instability | cAP O-C2 | Vascular: L VA thrombosis | 3 | IR arteriogram |
| Case 7 | 3yo M | Cornelia de Lange syndrome | C2-3 instability w/ severe spinal stenosis | PSF O-C3 | Implant: TA screw loosening | 3 | Implant revision, fusion extension |

*Author Affiliations and Disclosures: John B. Emans, MD, Boston Children's Hospital; Daniel Hedequist, MD, Boston Children's Hospital; M. Timothy Hresko, MD, Childrens Hospital Boston, Harvard Medical School; Maxwell H. McKee-Proctor, Boston Children's Hospital; Mark R. Proctor, MD, Boston Children's Hospital; Bram Verhofste, Boston Children's Hospital*

### Paper # 7.

### Respiratory and post-operative outcomes in early onset scoliosis patients treated with rib based growing system: single centre experience of 15 years

*Norman Ramirez-Lluch, MD, Lyanne Camacho, Mary Ibañez, Nicole Ramirez, Yashira Torres, Jose Montañez, Gerardo Olivella*

**Summary**: This is a 15-year experience follow-up study that characterizes respiratory, spinal deformity & post-op complications of early onset scoliosis (EOS) patients who underwent rib based growing system (RBGS).

**Hypothesis**: The purpose of this study was to evaluate the respiratory, spinal deformity & post-operative complications of EOS patients (pts) who underwent RBGS.

**Introduction**: The RBGS have been presented as an alternative to improve clinical status, spinal deformity & pulmonary function in patients with EOS. The aim of this study is to evaluate respiratory & post-op long-term outcomes in EOS pts who undergo a RBGS.

**Methods**: Retrospective single-center study of all EOS pts treated with a RBGS and repeated lengthening procedures every 6-months from 2005 to 2019 who had a minimum of 2 years of follow up. Demographic & primary coronal-sagittal Cobb angles were performed at preoperative, immediate postoperative, & last follow-up. Pulmonary function test (PFT) was evaluated at preoperative & last follow-up. Post-op complications were analyzed until last available examination.

**Results**: Thirty-six EOS pts (44.4% female) were enrolled in the study, with 20 neuromuscular, 10 congenital, 5 syndromic & 1 idiopathic. An average age of 7.2 + 2.9 years with a mean follow up of 6.6 + 3.4 years & mean of 6.0 + 3.0 subsequent lengthening procedures were found at 6-month intervals. Average preoperative coronal Cobb angle was 60.0 + 15.0? with immediate postoperative angle of 33.0 + 14.4?, & 60.0 + 23.5? angle at last follow-up. Mean preoperative sagittal Cobb angle showed 36.0 + 20.6? with immediate post-op angle of 27.9 + 13.2?, & 36.0 + 20.2? angle at last follow-up. Twenty-one out of 36 pts were able to complete PFTs with an average of 4 PFTs per patient. Mean FVC percentage changed from 77.3 + 41.0% initially to 46.8 + 14.5% at last expansion. The average of FEV1 changed from 74.3 + 37.7% initially to 47.8 + 14.9% at last expansion. A total of 38 complications in 21/36 pts were reported in the study. The most common complications were infection & wound dehiscence followed by device migration.

**Conclusion**: This 15-year follow-up study of EOS pts undergoing RBGS shows a concerning decrease in PFT and spinal correction with a high post-op complication rate at last follow up visit.

**Table I: Type and Rate of Postoperative Complications of Early-onset Scoliosis patients who underwent Rib Based Growing System**

| Variable | Frequency (%) |
|---|---|
| **Type of Complication (qty)** | N (%) = 38 complications |
| Infection & wound dehiscence | 13 (34.2) |
| Device Migration & related issues | 12 (31.6) |
| Hardware Failure | 5 (13.2) |
| Paraplegic, paralysis and neurologic | 3 (7.9) |
| Hemothorax | 2 (5.3) |
| Neurogenic Pain | 1 (2.6) |
| Pneumothorax | 1 (2.6) |
| Colon perforation | 1 (2.6) |
| Total | 38 (100.0) |
| **Rate of post-operative complications per patient (qty)** | N (%) = 36 pts |
| No complications | 15 (41.7) |
| One complication | 13 (36.1) |
| Two complications | 3 (8.3) |
| Three complications | 2 (5.6) |
| Four complications | 2 (5.6) |
| Five complications | 1 (2.8) |
| Total | 36 (100.0) |

*Author Affiliations and Disclosures: Lyanne Camacho, Ponce Health Sciences University; Mary Ibañez, Ponce Health Sciences University; Jose Montañez, Department of Orthopedic Surgery, University of Puerto Rico; Gerardo Olivella, Department of Orthopedic Surgery, University of Puerto Rico; Nicole Ramirez, University of Puerto Rico, Mayaguez Campus; Norman Ramirez-Lluch, MD, Hospital de la Concepcion—San German; Yashira Torres, Pediatric Department, Puerto Rico Women and Children's Hospital*

### Paper #8.

### Definitive fusions are better than growth friendly procedures for juvenile patients with cerebral palsy and scoliosis: a prospective comparative cohort study

*Arun R. Hariharan, MD, MS, Suken A. Shah, MD, Paul D. Sponseller, MD, Burt Yaszay, MD, Michael P. Glotzbecker, MD, Patrick J. Cahill, MD, Tracey Bastrom*

**Summary**: In this prospective comparative cohort study of fusion versus growth friendly surgery with minimum 2 year follow up from "definitive" surgery for juvenile CP scoliosis, we have shown that radiographic results were better in the PSF group and the complication rate and number of unplanned reoperations were higher in the GR group. Caregiver reported outcomes also rated PSF surgery with greater satisfaction.

**Hypothesis**: Juvenile patients with cerebral palsy (CP) who undergo fusion have fewer complications and better outcomes than those undergoing growth friendly surgery.

**Introduction**: In juvenile patients with CP and scoliosis, there is controversy regarding surgical treatment with posterior fusion (PSF) versus growing rod (GR) constructs. This study compared the outcomes of juvenile CP patients with scoliosis who underwent PSF versus GR.

**Methods**: Prospective registries were queried for patients 8–10 yrs old with minimum 2 yrs follow-up who underwent PSF or GR surgery. Demographics, radiographs, complications and outcomes scores were recorded.

**Results**: There were 41 patients in the PSF group and 15 in the GR group. The mean age at initial surgery was 10 yrs (8.1–10.9) and 9.3 yrs (8–10.9) in the PSF and GR groups, respectively ($p = 0.01$). In the PSF group, the mean preop curve was 86 deg and 80 deg in the GR group ($p = 0.20$). "Definitive" surgery in the GR group consisted of PSF in 10, implant retention in 3, and implant removal in 2. In the PSF group, 27% had complications ($n = 11$) and in the GR group, 73% had complications ($n = 11$) ($p = 0.02$) (see Table). In the PSF group, 2 patients had re-ops for infection while 8 patients in the GR group had re-ops for infection and implant complications ($p < 0.01$). In the PSF group, 26/35 parents (75%) noted that the child's life "improved a lot". In the GR group, 3/7 parents (43%) noted they were "neutral" about their child's ability to do things and 3/7 (43%) were "very dissatisfied".

**Conclusion**: Our study shows that PSF in juvenile CP patients with scoliosis results in fewer complications and reoperations and better radiographic outcomes compared to GR. Quality of life improvements were better in the PSF group.

| PSF Complications | Rate (n) |
|---|---|
| Infection | 7% (n=3) |
| Respiratory | 17% (n=7) |
| GI | 7% (n=3) |
| Medical | 2% (n=1) |
| **GR Complications** | **Rate (n)** |
| Infection | 46% (n=7) |
| Hardware | 13% (n=2) |
| PJK | 13% (n=2) |
| Respiratory | 20% (n=3) |
| Decubitus Ulcer | 13% (n=2) |

*Author Affiliations and Disclosures: Tracey Bastrom, Rady Children's Hospital; Patrick J. Cahill, MD, The Children's Hospital of Philadelphia; NuVasive Inc (Consultant), Children's Spine Foundation (Grants/Research), Setting Scoliosis Straight Foundation (Grants/Research); Michael P. Glotzbecker, MD, Rainbow Babies & Children's Hospital; Arun R. Hariharan, MD, MS, Nemours/A.I.DuPont Hospital for Children; Suken A. Shah, MD, Alfred I DuPont Hospital for Children; Paul D. Sponseller, MD, Johns Hopkins University; Burt Yaszay, MD, Rady Children's Hospital—San Diego*

### Paper # 9.

### Magnetic controlled growing rods (MCGR) vs single fusion vs tether in older EOS patients

*Jaime A. Gomez, MD, Catherine Mackey, Regina Hanstein, Majella Vaughan, Tricia St. Hilaire, MPH, Scott J. Luhmann, MD, Michael G. Vitale, MD MPH, Michael P. Glotzbecker, MD, Amer Samdani, Stefan Parent, MD PhD, Pediatric Spine Study Group.*

**Summary**: In ambulatory 8–11 y/o idiopathic EOS patients, MCGRs, PSF and VBT are effective in controlling scoliosis curves. VBT and PSF patients had fewer complications, fewer surgical complications and improved QoL compared to MCGR patients. Spinal growth similarly increased in all groups.

**Hypothesis**: In older idiopathic EOS patients, PSF and VBT provide curve control, improve QoL and reduce complications compared to MCGRs.

**Introduction**: MCGRs, PSF and VBT control scoliosis curves in older EOS patients. While MCGRs and VBT allow for spinal growth and preserve motion respectively, their outcomes compared to spinal fusion have not been compared.

**Methods**: 130 ambulatory idiopathic EOS patients from a multicenter registry, 81% female, aged 8–11 at index surgery (mean 10.5 yrs) were included. Scoliosis curve, Kyphosis, T1-S1 length, complications and QoL were assessed pre-operatively and at most recent FU (mean 3.4 yrs ± 1.5).

**Results**: 51 MCGR, 42 PSF and 37 VBT patients included (Table 1). Pre-operatively, VBT patients were older, had smaller curves and less kyphosis compared to PSF or MCGR patients ($p < 0.005$). At recent FU, scoliosis curve had decreased in all groups ($p < 0.0005$), but curves were smaller in VBT and PSF compared to MCGR patients ($p < 0.0005$). Kyphosis changed with VBT and PSF ($p = 0.005$) but was similar between all groups at recent FU. Although pre-operative T1-S1 length was smaller in the MCGR group compared to the other groups ($p = 0.025$), it increased in all groups by 5 cm at FU. 15 complications occurred in 10 VBT patients (27%), 9 in 6 PSF patients (13%), and 43 in 30 (59%) MCGR patients ($p < 0.0001$; Table 2). Complications that required surgery occurred in 8 VBT, 5 PSF and 25 MCGR patients ($p < 0.0005$). At recent FU, 16 MCGR had definitive fusions and 2 tethers required fusion. QoL improved for VBT and PSF patients ($p < 0.05$), but not for MCGR patients (Table 3).

**Conclusion**: While MCGRs, PSF and VBT control curves effectively and increase spinal height in older EOS patients, VBT and PSF patients had less complications and improved QoL.

**Table 1 Demographic and Radiographic Parameters of 130 idiopathic EOS Patients**

| Demographics | VBT N=37 | MCGR N=51 | PSF N=42 | p-value |
|---|---|---|---|---|
| Female, n (%) | 36 (97%) | 35 (69%) | 34 (81%) | ***0.002*** |
| Age, median (IQR) | 11.3 (10.9, 11.8) | 9.6 (8.8, 10.2) | 10.9 (10.3, 11.5) | ***<0.005*** |
| Follow-Up (FU), median (IQR) | 3.0 (2.1, 3.6) | 2.9 (2.4, 3.9) | 3.6 (2.2, 5.1) | *0.052* |
| BMI % for age pre-op, median (IQR) | 45 (16.5, 69.5) | 45 (27, 76) | 64.5 (20, 91) | *0.312* |
| BMI % for age @ FU, median (IQR) | 59 (35, 82) | 57 (17.5, 76.5) | 68.5 (36, 88) | *0.503* |
| **Scoliosis curve** | | | | |
| Major Scoliosis angle pre-op (°), median (IQR) | 50 (43.5, 58) | 64.5 (55, 75) | 63 (57, 72) | ***<0.0005*** |
| Major Scoliosis angle @ FU (°), median (IQR) | 27 (21, 32) | 40 (31, 50.5) | 29 (22, 36) | ***<0.0005*** |
| *Major scoliosis angle pre-op vs. FU within Surgery group, p-value* | ***<0.0005*** | ***<0.0005*** | ***<0.0005*** | |
| Δ Scoliosis angle pre to FU (%), mean ± SD | 41.1 ±22.4 | 28.7 ±24.4 | 52.2 ±19.9 | ***<0.0005*** |
| **Kyphosis (maximum)** | | | | |
| Kyphosis pre-op (°), median (IQR) | 26 (20.5, 32) | 39 (33, 49) | 49 (34, 60) | ***<0.0005*** |
| Kyphosis @ FU (°), median (IQR) | 40 (29, 50) | 37.6 (25.5, 52) | 47 (35.0, 64.0) | *0.366* |
| *Kyphosis pre-op vs. FU within Surgery group, p-value* | ***0.005*** | ***0.420*** | ***<0.0005*** | |
| Δ Kyphosis pre-op to FU (°), mean ± SD | 8.6 ±15.9 | -2.0 ±22.8 | -13.6 ±17.0 | ***<0.0005*** |
| **Spinal height (T1-S1)** | | | | |
| T1-S1 pre-op (cm), median (IQR) | 34.2 (31.8, 35.6) | 31.5 (29.7, 35.7) | 35.4 (32.6, 37.3) | ***0.025*** |
| T1-S1 @ FU (cm), median (IQR) | 39.6 (36.8, 40.7) | 35.9 (33.8, 39.5) | 39.8 (36.9, 43) | ***0.003*** |
| *T1-S1 pre-op vs. FU within Surgery group, p-value* | ***<0.0005*** | ***<0.0005*** | ***<0.0005*** | |
| Δ T1-S1 pre-op to FU (cm), mean ± SD | 5.0 ±2.0 | 5.0 ±5.0 | 5.8 ±6.8 | *0.827* |

*n=number of patients; IQR: interquartile range*
*Statistics: Fisher's exact test, Kruskal Wallis test or Anova*
*FU=most recent FU; for VBT and MCGR patients with definitive fusion, radiographic FU was prior to the fusion.*

**Table 2 Complications**

| | VBT N=37 | MCGR N=51 | PSF N=42 | p-value |
|---|---|---|---|---|
| Complications, n (%) | 10 (27) | 30 (58.8) | 6 (13) | ***<.0001*** |
| # Complications | 15 | 43 | 9 | |
| Minor complications, n (%) | 4 (10.8) | 9 (17.6) | 2 (4.8) | *0.151* |
| # Minor complications | 5 | 11 | 3 | |
| Surgical Complication, n (%) | 8 (21.6) | 25 (49) | 5 (11.9) | ***<0.0005*** |
| # Surgical Complications | 10 | 31 | 6 | |
| Hardware Issue, n (%) | 5 (13.5) | 10 (19.6) | 1 (2.4) | ***0.023*** |

*n=number of patients, # number of*
*Statistics: chi square or Fisher's exact test*

**Table 3: Health-related Quality of Life - EOS Questionnaire 24 (EOSQ-24)**

| EOSQ-24 Domains, median (IQR) | VBT N=10 | MCGR N=30 | PSF N=16 | p-value |
|---|---|---|---|---|
| General Health pre-op | 75 (75, 87.5) | 75 (75, 87.5) | 81.3 (68.8, 87.5) | *0.987* |
| General Health @ FU | 62.5 (62.5, 87.5) | 75 (62.5, 100) | 93.8 (68.8, 100) | *0.103* |
| *pre-op to FU within surgical group* | *0.211* | *0.463* | *0.241* | |
| Pain/Discomfort pre-op | 56.3 (37.5, 62.5) | 75 (50, 100) | 75 (62.5, 100) | *0.058* |
| Pain/Discomfort @ FU | 75 (50, 100) | 75 (50, 93.8) | 75 (50, 93.8) | *0.963* |
| *pre-op to FU within surgical group* | ***0.023*** | *0.381* | *0.306* | |
| Pulmonary Function pre-op | 93.8 (87.5, 100) | 100 (75, 100) | 100 (87.5, 100) | *0.894* |
| Pulmonary Function @ FU | 100 (75, 100) | 100 (87.5, 100) | 100 (87.5, 100) | *0.889* |
| *pre-op to FU within surgical group* | *1.000* | *0.624* | *0.828* | |
| Transfer pre-op | 100 (50, 100) | 100 (100, 100) | 100 (100, 100) | *0.069* |
| Transfer @ FU | 100 (100, 100) | 100 (75, 100) | 100 (100, 100) | *0.155* |
| *pre-op to FU within surgical group* | *0.313* | *0.065* | *1.000* | |
| Physical Function pre-op | 95.8 (83.3, 100) | 100 (83.3, 100) | 100 (95.8, 100) | *0.453* |
| Physical Function @ FU | 100 (91.7, 100) | 95.8 (75, 100) | 100 (95.8, 100) | *0.178* |
| *pre-op to FU within surgical group* | *0.500* | *0.661* | *1.000* | |
| Daily Living pre-op | 100 (100, 100) | 100 (75, 100) | 100 (100, 100) | ***0.009*** |
| Daily Living @ FU | 100 (87.5, 100) | 100 (75, 100) | 100 (100, 100) | ***0.028*** |
| *pre-op to FU within surgical group* | *0.250* | *0.555* | *1.000* | |
| Fatigue/Energy pre-op | 87.5 (62.5, 87.5) | 100 (75, 100) | 100 (75, 100) | *0.183* |
| Fatigue/Energy @ FU | 75 (62.5, 100) | 87.5 (75, 100) | 93.8 (87.5, 100) | *0.328* |
| *pre-op to FU within surgical group* | *0.816* | *0.140* | *0.725* | |
| Emotion pre-op | 75 (50, 75) | 62.5 (62.5, 75) | 81.3 (68.8, 87.5) | *0.112* |
| Emotion @ FU | 93.8 (75, 100) | 81.3 (50, 100) | 87.5 (75, 100) | *0.057* |
| *pre-op to FU within surgical group* | ***0.020*** | *0.062* | *0.090* | |
| Parental Impact pre-op | 70 (55, 80) | 75 (65, 95) | 90 (80, 95) | ***0.017*** |
| Parental Impact @ FU | 87.5 (75, 95) | 90 (75, 95) | 100 (85, 100) | *0.053* |
| *pre-op to FU within surgical group* | ***0.020*** | *0.206* | ***0.005*** | |
| Child Satisfaction pre-op | 75 (50, 75) | 75 (50, 100) | 100 (75, 100) | *0.059* |
| Child Satisfaction @ FU | 75 (75, 100) | 75 (50, 100) | 100 (75, 100) | *0.059* |
| *pre-op to FU within surgical group* | *0.219* | *0.873* | *1.000* | |
| Parent Satisfaction pre-op | 62.5 (50, 75) | 75 (50, 100) | 100 (75, 100) | *0.089* |
| Parent Satisfaction @ FU | 75 (75, 100) | 75 (50, 100) | 100 (75, 100) | ***0.038*** |
| *pre-op to FU within surgical group* | *0.063* | *0.563* | *0.781* | |
| *Kruskal Wallis test or Wilcoxon signed rank test FU=most recent FU* | | | | |

*Author Affiliations and Disclosures: Michael P. Glotzbecker, MD, Rainbow Babies & Children's Hospital; Jaime A. Gomez, MD, Montefiore/Albert Einstein College of Medicine; Regina Hanstein, Children's Hospital at Montefiore Medical Center; Scott J. Luhmann, MD, Washington University School of Medicine; Catherine Mackey, Montefiore Medical Center; Stefan Parent, MD PhD, CHU Hôpital Ste-Justine; Amer Samdani, Shriners Hospitals for Children, Philadelphia; Tricia St. Hilaire, MPH, Children's Spine Foundation; Pediatric Spine Study Group, Children's Spine Foundation; Majella Vaughan, Children's Spine Foundation; Michael G. Vitale, MD MPH, Columbia University Medical Center/Morgan Stanley Children's Hospital; Pediatric Spine Study Group, Pediatric Orthopaedic Society of North America (Grants/Research), FDA (Grants/Research), NuVasive (Grants/Research)*

### Paper # 10.

### Casting for non-idiopathic EOS (NIEOS)—combined results from 2 centers

*Jacques D'Astous, Graham Fedorak, Dong-Phuong Tran, MS, David C. Thornberg, Charles E. Johnston, MD*

**Summary**: Casting for NIEOS controlled (= surgery avoided) 16/46 cases, compared to 57/77 idiopathic (EOIS) cases ($p < 0.001$) at mean 7 yr follow-up. NIEOS pts were older (3.7 vs 2.1 yrs, $p = 0.000$) at 1st cast and had larger deformity (62.5 vs 53.5°, $p < 0.002$) than EOIS. Patients requiring surgery were also older at 1st cast, had larger RVAD and Cobb compared to successful cast patients.

**Hypothesis**: NIEOS curves (congenital, syndromic, N-M) can be treated by casting with similar efficacy as in EOIS.

**Introduction**: Serial casting has become a viable treatment for IEOS < 3 yrs of age to control or cure curvature. We aim to report on results of serial casting in a NIEOS cohort at two experienced casting centers.

**Methods**: Age at 1st cast, # of casts, treatment after casting, clinical outcome (observation, bracing, surgery) were determined along with curve magnitude, T1-12/T1-S1 lengths were compared prior to 1st cast and at latest follow-up (min of 5 years from 1st cast). Cast failure = progression to surgery (last visit before surgery); success = continued brace or observation.

**Results**: 123 pts were reviewed at a mean 7 yrs from 1st cast; 46 NIEOS, 77 EOIS (68 and 92-month follow-up) ($p < 0.001$). Specifics regarding NIEOS cohort outlined in Table 1 below. NIEOS cohort were older at 1st cast (3.7 vs 2.1 yr $p = 0.000$) with no difference in #casts (6.2). Initial curve was larger in NIEOS (62.5 vs 53.5°, $p < 0.002$) as was RVAD (37.1 vs 24.0°, $p < 0.01$). At follow-up curvature was 74° (range 13°–126°) for entire NIEOS group vs 36° (0°–105°) for all EOIS $p = 0.000$; T1-12 (16.3 vs 20.5 cm) and T1-S1 (26.7 vs 33.7 cm, both $p = 0.000$) were both longer in EOIS pts. Success was achieved in 16 NIEOS (4 observation,11 in brace, 1 cure), and 57 EOIS (24 observation, 27 brace,6 cure). Cases requiring surgery were older at 1st cast (3.8 vs 2.0 yr), had larger RVAD (33.5 vs 23.7°) and Cobb (65.2 vs 51.4°). There was no difference in number of abnormal MRIs comparing success vs surgery.

**Conclusion**: Casting is worth trying for NIEOS, expecting less success compared to EOIS results. However, NIEOS curves were larger and patients older at 1st cast application.

| Diagnosis Main | Sub Group 1 "Stiff" | Sub Group 2 "Hyperlax" |
|---|---|---|
| Syndromic (n=23) | AMC, Marfan, Beals (n=10) | Prader-Willi, Ehler-Danlos (n=13) |
| Neuro Muscular (n=10) | | |
| Congenital {Including NF-1} (n=13) | | |
| **NIEOS Total from above (n=46)** | | |
| **IEOS (n=77)** | | |

*Author Affiliations and Disclosures: Jacques D'Astous, Shriners Hospital Salt Lake City; Graham Fedorak; Charles E. Johnston, MD, Texas Scottish Rite Hospital; Anna M. McClung, Researcher, Texas Scottish Rite Hospital for Children; David C. Thornberg, Texas Scottish Rite Hospital for Children; Dong-Phuong Tran, MS, Texas Scottish Rite Hospital*

### Paper # 11.

### Exposure and variability of anesthesia during mehta casting procedures in early onset scoliosis (EOS)

*Lisa Bonsignore-Opp, Jacob Ball, Matthew Simhon, Hiroko Matsumoto, PhD, Peter F. Sturm, MD, Joshua Pahys, Michael G. Vitale, MD MPH, Annalise N. Larson, MD, Benjamin D. Roye, MD-MPH, Pediatric Spine Study Group*

**Summary**: Variability of anesthesia exposure during Mehta casting for EOS has not been studied. 208 EOS patients who underwent 1097 Mehta casting procedures were identified. Anesthesia time varied significantly between institutions and 75% of patients < 3 yrs old exceeded the 3 h cumulative threshold identified by the FDA as potentially associated with developmental delay. Anesthesia time was reduced when performing procedures using specialized Mehta casting tables.

**Hypothesis**: There exists significant institutional variability in anesthesia exposure during Mehta casting procedures that is attributed to modifiable factors.

**Introduction**: Mehta casting is a potentially curative intervention for EOS that typically requires multiple anesthetics. The FDA reported that > 3 h of anesthesia before age 3 may alter brain development; however, no standard exists for duration of anesthetic during casting. The purpose of this study is to quantify variability in anesthesia during Mehta casting.

**Methods**: An EOS registry identified patients who underwent at least one Mehta casting procedure. Anesthesia exposure was quantified and site variability was assessed by patient characteristics, cast placement, procedure type, and equipment used.

**Results**: 208 patients from 5 institutions were identified (age 2.6 ± 1.4 yo). There were 1097 Mehta casting procedures with 5.4 ± 3.6 castings per patient. Anesthesia time for casting was 69 ± 31 min and varied significantly between sites (59 ± 14–117 ± 46 min; p < 0.001). Cumulative anesthesia time for patients under 3 yo was 320 ± 197 min with 120/161 (74.5%) patients exceeding 3 h. Use of tables not specifically designed for Mehta casting was associated with significantly increased anesthesia exposure (93 ± 44 vs. 64 ± 25 min, P < 0.001). Among idiopathic patients undergoing application only casting procedures, anesthesia time was 62 ± 19 min when using Mehta tables compared to 89 ± 49 min for non-specific tables (p = 0.001).

**Conslusion**: Patients undergoing Mehta casting are at significant risk of exceeding 3hrs of anesthesia which the FDA has stated may be harmful for children < 3 yo. Significant site variability indicates that standardization protocols, including use of Mehta casting tables, should be developed to encourage best practice.

*Author Affiliations and Disclosures: Jacob Ball, Columbia University; Lisa Bonsignore-Opp, Columbia University; Annalise N. Larson, MD, Mayo Clinic; Hiroko Matsumoto, PhD, Columbia University; Joshua Pahys, Shriners Hospitals for Children; Benjamin D. Roye, MD-MPH, Columbia University; Matthew Simhon, Columbia University; Pediatric Spine Study Group, Children's Spine Foundation; Peter F. Sturm, MD, Cincinnati Children's Hospital Medical Center; Michael G. Vitale, MD MPH, Columbia University Medical Center/Morgan Stanley Children's Hospital; Pediatric Spine Study Group, Pediatric Orthopaedic Society of North America (Grants/Research), FDA (Grants/Research), NuVasive (Grants/Research)*

### Paper # 12.

### Use of vancomycin powder in guided growth procedures for early onset scoliosis is associated with different microbiology cultures after surgical site infection

*Jerry Y. Du, Anne Marie Dumaine, Stefan Parent, MD PhD, Peter F. Sturm, MD, Paul D. Sponseller, MD, Michael P. Glotzbecker, MD*

**Summary**: In this retrospective cohort analysis of a multicenter database, the use of vancomycin powder in early onset scoliosis patients undergoing guided growth procedures complicated by surgical site infections (SSI) was independently associated with higher risk of no culture growth.

**Hypothesis**: The use of vancomycin powder in EOS patients undergoing guided growth procedures will alter the microbiology of SSI.

**Introduction**: The use of vancomycin powder has been shown to decrease risk of surgical site infection in early onset scoliosis (EOS) patients undergoing guided growth procedures. However, the effects of vancomycin powder on microbiology in patients that develop SSIs has not been studied.

**Methods**: A multicenter database for EOS patients was retrospectively analyzed. All patients that underwent guided growth surgery for EOS performed after 2010 that sustained at least one SSI with culture and antibiotic details were included. Cohorts were constructed based on vancomycin powder use at index surgery. A multivariate regression model was used to control for potential confounding factors, including use of other antibiotics.

**Results**: There were 55 patients included in this study, including 26 males (47.3%) and 29 females (52.7%). Mean age at guided growth procedure insertion was 7.2 ± 6.9 years. Vancomycin powder was utilized in 18 cases (32.7%). Mean time from guided growth procedure to SSI event was 2.0 ± 1.3 years.

**Conclusion**: In EOS patients undergoing guided growth procedures complicated by SSI, the use of vancomycin powder was independently associated with increased risk of no culture growth. Surgeons and infectious disease physicians should be aware that vancomycin powder may alter microbiology environments and should adjust their treatment strategies accordingly.

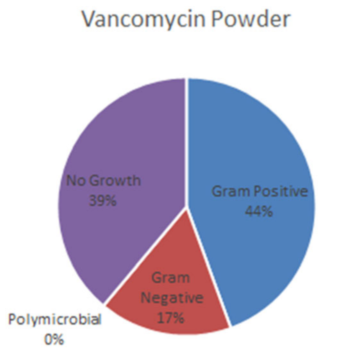

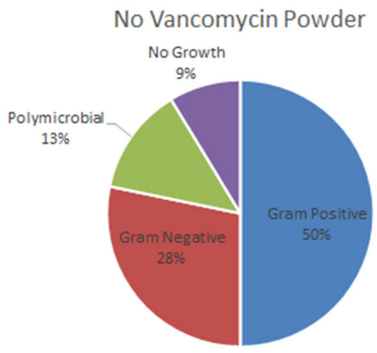

Table 1. Microbiology details

| Bacteria | Vancomycin Powder (n=18) | No Vancomycin Powder (n=37) | Relative Risk | 95% Confidence Interval | P-value |
|---|---|---|---|---|---|
| Gram Positive | 8 (44.4%) | 23 (62.2%) | 0.786 | 0.529-1.169 | 0.214 |
| Gram Negative | 3 (16.7%) | 13 (35.1%) | 0.757 | 0.538-1.066 | 0.213 |
| Polymicrobial | 1 (5.6%) | 7 (18.9%) | 0.729 | 0.520-1.024 | 0.250 |
| No Growth | 7 (38.9%) | 4 (10.8%) | 2.063 | 0.927-4.591 | **0.028** |
| S. aureus | 6 (33.3%) | 16 (43.2%) | 0.875 | 0.608-1.258 | 0.481 |
| Methicillin Resistant S. aureus | 1 (25.0%) | 2 (16.7%) | 1.154 | 0.491-2.710 | >0.999 |
| Coagulase negative staphylococcus | 1 (5.6%) | 7 (18.9%) | 0.729 | 0.520-1.024 | 0.250 |
| S. epidermidis | 2 (11.1%) | 0 (0%) | - | - | 0.103 |
| E. coli | 0 (0%) | 3 (8.1%) | - | - | 0.543 |
| P. aeruginosa | 3 (16.7%) | 7 (18.9%) | 0.952 | 0.604-1.502 | >0.999 |
| E. faecalis | 0 (0%) | 1 (2.7%) | - | - | >0.999 |
| K. pneumoniae | 0 (0%) | 1 (2.7%) | - | - | >0.999 |
| Other gram positive spp. | 0 (0%) | 1 (2.7%) | - | - | >0.999 |
| Other gram negative spp. | 0 (0%) | 4 (10.8%) | - | - | 0.291 |

*Author Affiliations and Disclosures: Jerry Y. Du, Rainbow Babies &*

*Children's Hospitals; Anne Marie Dumaine, University Hospitals of Cleveland; Michael P. Glotzbecker, MD, Rainbow Babies & Children's Hospital; Stefan Parent, MD PhD, CHU Hôpital Ste-Justine; Paul D. Sponseller, MD, Johns Hopkins University; Peter F. Sturm, MD, Cincinnati Children's Hospital Medical Center*

### Paper # 13.

#### Musculocutaneous flap coverage to salvage rib-based construct hardware complications

*Christopher L. Kalmar, Zachary D. Zapatero, Carina Lott, MS, Patrick J. Cahill, MD, Laura S. Humphries, Carrie E. Zimmerman, Giap H. Vu, Jordan W. Swanson, Jason B. Anari, MD, Jesse A. Taylor*

**Summary**: We aim to examine flap choice and salvage rates of rib-based construct wound complications in early onset scoliosis (EOS) patients.

**Hypothesis**: Musculocutaneous flap coverage plays an important role in salvage of rib-based construct wound compilations.

**Introduction**: EOS patients are often treated with rib-based devices that often result in wound breakdown, implant exposure, and infection. At the onset of these device complications, musculocutaneous flaps offer robust vascular supply and tissue coverage, which has provided promise in preventing need for hardware removal. We examined flap choice and salvage rates of rib-based construct wound complications.

**Methods**: We identified EOS patients who underwent musculocutaneous flap coverage at our institution between 2014 and 2020. Wounds were categorized by location and indication for surgery. Salvage was considered successful if the hardware was able to be retained and the patient did not require any operative procedure at that site until their subsequent rib-based expansion surgeries.

**Results**: 43 patients underwent musculocutaneous flap coverage at a median of 3.7 yrs and 6.5 expansions after initial hardware placement. Indications for flap coverage were exposed hardware (58%) and threatened exposure (42%). Most common location requiring flap coverage was the middle back (61%), followed by the upper back (34%). Most common location for successful salvage was the lower back (83%), followed by upper back (73%) and middle back (73%). Most common flap used was the latissimus muscle (61%) followed by paraspinous muscles (28%). 74% of wound complications requiring plastic surgery were successfully salvaged. Latissimus flaps achieved successful salvage in 65% (n = 17 of 26) patients with a success range of 67–80.0% at each region of the back. Paraspinous flaps achieved successful salvage in 92% (n = 11 of 12) patients with a success range of 67–100.0% at each region of the back. Presence of infection was significantly implicated in flap failure (p = 0.011; OR 6.5, 95%CI 1.4–29.4).

**Conclusion**: Musculocutaneous flaps demonstrated reasonable salvage rates for wound complications. While the latissimus was most commonly used, other pedicled flaps played an important role in salvage of wound complications.

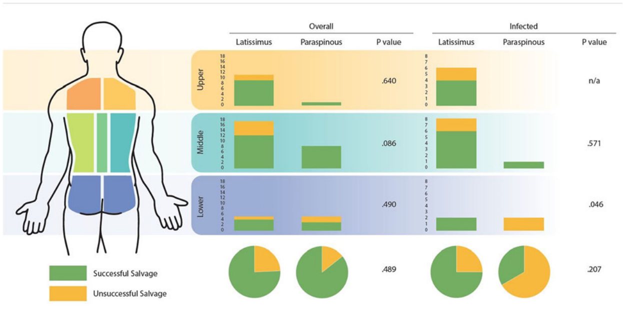

**Figure 1.** Flap choice, location, and salvage rates in early onset scoliosis patients with hardware complications.

*Author Affiliations and Disclosures: Jason B. Anari, MD, Children's Hospital of Philadelphia; Patrick J. Cahill, MD, The Children's Hospital of Philadelphia; NuVasive Inc (Consultant), Children's Spine Foundation (Grants/Research), Setting Scoliosis Straight Foundation (Grants/Research); Laura S. Humphries, Children's Hospital of Philadelphia; Christopher L. Kalmar, Children's Hospital of Philadelphia; Carina Lott, MS, Children's Hospital of Philadelphia; Jordan W. Swanson, Children's Hospital of Philadelphia; Jesse A. Taylor, Children's Hospital of Philadelphia; Giap H. Vu, Children's Hospital of Philadelphia; Zachary D. Zapatero, Children's Hospital of Philadelphia; Carrie E. Zimmerman, Children's Hospital of Philadelphia*

### Paper # 14.

#### Quantity vs quality of distal fixation points in neuromuscular early onset scoliosis: does it matter?

*Jaysson T. Brooks, MD, Robert F. Murphy, MD, Ron El-Hawary, MD, Tyler Mcdonald, Paul D. Sponseller, MD, Michael G. Vitale, MD MPH, Matthew Oetgen, MD MBA, Patrick Wright, Ryan E. Fitzgerald, Patrick J. Cahill, MD, Hamdi Sukkarieh*

**Summary**: The quantity of distal fixation points does not affect outcomes in eoNMS, but the quality of fixation does. Instrumentation with bilateral SAI screws yields the lowest complications in patients with eoNMS.

**Hypothesis**: The quantity of fixation is most important to decrease complications in eoNMS.

**Introduction**: Modern pelvic fixation for patients with eoNMS includes sacral-alar-iliac (SAI) or iliac screws (IS), however there is no consensus on which construct is better, or how many points of distal fixation are required to decrease complications, or whether augmentation with S1 screws is important. The purpose of this study is to determine if the number of distal fixation points or the quality of distal fixation points affects postoperative outcomes in patients with eoNMS.

**Methods**: A database was queried for all eoNMS patients, treated with growing rods anchored to the pelvis with SAI or IS, yielding 57

patients. The quantity of distal fixation was measured, and the quality of fixation was formed from 5 constructs (Table 2). The mean follow-up was 3.4 ± 2 years and there were only 16 UPROR events for the cohort. There was no difference in UPRORs or complications based on the quantity of fixation (Table 1). There was also no significant correlation between the quantity of fixation and pelvic obliquity (p = 0.75). Further, the quality of distal fixation had no effect on UPRORs, but it did have a significant effect on complications (Table 2). Sub-group analysis (Table 3) showed a significantly higher rate of distal fixation complications in patients with a mixed distal fixation (construct 5). In addition, patients with construct 5 had a significantly higher pelvic obliquity (18 ± 13 deg.) than patients with constructs 1 through 4 (mean pelvic obliquity = 7.2–8.2 deg., p = 0.02). There was a significantly higher rate of complications in patients whose pelvic fixation included bilateral iliac screws (67%) compared to patients with bilateral SAI screws (33%, p = 0.035).

**Results**: The mean follow-up was 3.4 ± 2 years and there were only 16 UPROR events for the cohort. There was no difference in UPRORs or complications based on the quantity of fixation (Table 1). There was also no significant correlation between the quantity of fixation and pelvic obliquity (p = 0.75). Further, the quality of distal fixation had no effect on UPRORs, but it did have a significant effect on complications (Table 2). Sub-group analysis (Table 3) showed a significantly higher rate of distal fixation complications in patients with a mixed distal fixation (construct 5). In addition, patients with construct 5 had a significantly higher pelvic obliquity (18 ± 13 deg.) than patients with constructs 1 through 4 (mean pelvic obliquity = 7.2–8.2 deg., p = 0.02). There was a significantly higher rate of complications in patients whose pelvic fixation included bilateral iliac screws (67%) compared to patients with bilateral SAI screws (33%, p = 0.035).

**Conclusion**: The quantity of distal fixation points does not affect outcomes in eoNMS, but the quality of fixation does. Instrumentation with bilateral SAI screws yields the lowest complications in patients with eoNMS.

Table 1. UPRORs and Distal Fixation Complications Based on Quantity of Distal Fixation Points

| *Quantity of Fixation* | UPROR Distal Fixation | | P | UPROR Infection | | P | UPROR Rods | | P | Distal Fixation Complications | | P |
|---|---|---|---|---|---|---|---|---|---|---|---|---|
| | Yes | No | | Yes | No | | Yes | No | | Yes | No | |
| Mean # of Distal Fixation Points (Post-Index) | 2 | 5 ± 2 | 0.12 | 5 ± 2 | 4.8 ± 2 | 0.8 | 5.6 ± 1 | 4.6 ± 2 | **.045** | 3.6 ± 2 | 5 ± 2 | 0.14 |
| Mean # of Distal Fixation Points (Final Follow-up) | 2 | 5 ± 2 | 0.08 | 5 ± 2 | 4.9 ± 2 | 0.8 | 5.4 ± 1 | 4.9 ± 2 | 0.27 | 4.8 ± 2 | 5 ± 2 | 0.85 |

Table 2. UPRORs and Distal Fixation Complications Based on Quality of Distal Fixation Points

| *Distal Fixation Constructs* | UPROR Distal Fixation (n=51) | | UPROR Rod (n=51) | | UPROR Infection (n=52) | | Distal Fixation Complications (n=57) | |
|---|---|---|---|---|---|---|---|---|
| | Yes | No | Yes | No | Yes | No | Yes | No |
| Bilateral S1 & SAI screws | 0% | 100% (20) | 25% (5) | 75% (15) | 5% (1) | 95% (19) | 13% (3) | 87% (20) |
| Bilateral SAI screws only | 0% | 100% (14) | 21% (3) | 79% (11) | 21% (3) | 79% (11) | 6% (1) | 94% (15) |
| Bilateral S1 & Iliac screws | 0% | 100% (3) | 33% (1) | 66% (2) | 33% (1) | 67% (2) | 0% | 100% (3) |
| Bilateral Iliac Screws only | 0% | 100% (9) | 0% | 100% (9) | 11% (1) | 89% (8) | 11% (1) | 89% (8) |
| *Mixed Construct | 20% (1) | 80% (4) | 0% | 100% (5) | 0% | 100% (5) | 60% (3) | 40% (2) |
| p-value | 0.052 | | 0.528 | | 0.385 | | **0.041** | |

*A mixed construct included either a unilateral IS or SAI screw, or an iliac screw on one side and a SAI screw on the other.

Table 3. Sub-Group Analysis of Distal Fixation Complications Based on Quality of Distal Fixation Points

| *Distal Fixation Construct* | Distal Fixation Complications | | p-value |
|---|---|---|---|
| | Yes | No | |
| Bilateral S1 & SAI screws VS | 13% (3) | 87% (20) | |
| Bilateral SAI screws only | 6% (1) | 94% (15) | 0.492 |
| Bilateral S1 & Iliac screws | 0% | 100% (3) | 0.506 |
| Bilateral Iliac Screws only | 11% (1) | 89% (8) | 0.882 |
| Mixed Construct | 60% (3) | 40% (2) | **0.020** |
| | | | |
| Bilateral SAI screws only VS | 6% (1) | 94% (15) | |
| Bilateral S1 & Iliac screws | 0% | 100% (3) | 0.656 |
| Bilateral Iliac Screws only | 11% (1) | 89% (8) | 0.667 |
| Mixed Construct | 60% (3) | 40% (2) | **0.008** |
| | | | |
| Bilateral S1 & Iliac screws VS | 0% | 100% (3) | |
| Bilateral Iliac Screws only | 11% (1) | 89% (8) | 0.546 |
| Mixed Construct | 60% (3) | 40% (2) | 0.090 |
| | | | |
| Bilateral Iliac Screws only VS | 11% (1) | 89% (8) | |
| Mixed Construct | 60% (3) | 40% (2) | 0.052 |

*Author Affiliations and Disclosures: Jaysson T. Brooks, MD, Children's of Mississippi, OrthoPediatrics (Consultant), Depuy-Synthes (Consultant); Patrick J. Cahill, MD, The Children's Hospital of Philadelphia; NuVasive Inc (Consultant), Children's Spine Foundation (Grants/Research), Setting Scoliosis Straight Foundation (Grants/Research); Ron El-Hawary, MD, IWK Health Centre; Depuy Synthes (Grants/Research), Medtronic (Grants/Research), Orthopediatrics (Consultant, Advisory Board or Panel, Stock/Shareholder (self-managed));Ryan E. Fitzgerald, Riley Hospital for Children; Tyler Mcdonald, Children's of Mississippi; Robert F. Murphy, MD, Medical University of South Carolina; Matthew Oetgen, MD MBA, Children's National Health System; Paul D. Sponseller, MD, Johns Hopkins University; Hamdi Sukkarieh, Children's of Mississippi; Michael G. Vitale, MD MPH, Columbia University Medical Center/ Morgan Stanley Children's Hospital; Patrick Wright, Children's of Mississippi*

### Paper # 15.

### When is pelvic fixation necessary in children with cerebral palsy and scoliosis treated with growing rod constructs?

*Ying Li, MD, Jennylee Swallow, Joel Gagnier, John T. Smith, MD, Robert F. Murphy, MD, Patrick J. Cahill, MD, Paul D. Sponseller, MD, Pediatric Spine Study Group*

**Summary**: Lower baseline L5 tilt may be an indication to exclude the pelvis at the time of growing rod (GR) insertion in children with CP scoliosis. Distal spine anchors may provide better long-term control of the major curve than distal pelvic anchors.

**Hypothesis**: The pelvis can be successfully excluded in properly selected children with CP scoliosis treated with GR.

**Introduction**: Previous studies have shown that lower preoperative pelvic obliquity and L5 tilt, and greater thoracic kyphosis were associated with good radiographic outcomes when the fusion ended short of the pelvis in children with neuromuscular scoliosis. Our purpose was to identify indications to exclude the pelvis in children with CP scoliosis treated with GR.

**Methods**: Children with CP scoliosis treated with TGR, MCGR, or rib-based growing constructs with minimum 2-year follow-up after the index surgery were identified.

**Results**: 98 patients were identified. 27 patients had distal spine anchors (DSA) and 71 patients had distal pelvic anchors (DPA) placed at the index surgery. The only pre-index difference was lower pelvic obliquity in DSA (7 o vs 13 o, P = 0.020) (Table 1). Post-index and most recent radiographic data were similar except DSA had a smaller final major curve (47 o vs 58 o, P = 0.038).

**Conclusion**: Lower L5 tilt may be an indication to exclude the pelvis at the time of GR insertion for CP scoliosis. Study limitations include incomplete pre-index EOSQ-24 and not all patients have undergone final fusion.

**Table 1.** Comparison of patients with distal spine anchors vs distal pelvic anchors placed at the index surgery.

| *Demographics* | Distal spine anchors (N=27) | Distal pelvic anchors (N=71) | p-value |
|---|---|---|---|
| Age at index surgery (years; mean ± SD) | 6.8 ± 2.2 | 7.7 ± 2.0 | 0.069 |
| BMI at index surgery (kg/m$^2$; mean ± SD) | 15.7 ± 2.7 | 16.9 ± 3.0 | 0.145 |
| Nonambulatory status at index surgery [n (%)] | 19 (70) | 46 (65) | 0.098 |
| Length of follow-up (years; mean ± SD) | 5.9 ± 3.2 | 4.9 ± 2.6 | 0.093 |
| | | | |
| *Radiographic data* | | | |
| Pre-index major curve (°; mean ± SD) | 78 ± 23 | 80 ± 23 | 0.718 |
| **Pre-index pelvic obliquity (°; mean ± SD)** | **7 ± 5** | **13 ± 9** | **0.020** |
| Pre-index L5 tilt (°; mean ± SD) | 12 ± 7 | 14 ± 10 | 0.669 |
| Pre-index maximum sagittal deformity (°; mean ± SD) | 63 ± 22 | 59 ± 25 | 0.473 |
| Post-index major curve (°; mean ± SD) | 47 ± 18 | 44 ± 19 | 0.540 |
| Post-index pelvic obliquity (°; mean ± SD) | 8 ± 6 | 7 ± 5 | 0.762 |
| Post-index maximum sagittal deformity (°; mean ± SD) | 39 ± 17 | 37 ± 17 | 0.724 |
| **Most recent major curve (°; mean ± SD)** | **47 ± 22** | **58 ± 23** | **0.038** |
| Most recent maximum sagittal deformity (°; mean ± SD) | 50 ± 22 | 56 ± 25 | 0.367 |
| | | | |
| *Outcomes* | | | |
| **Total complications (n)** | **37** | **152** | **0.015** |
| Number of patients with complications [n (%)] | 20 (74) | 50 (70) | 0.569 |
| Average complications per patients (n) | 1.4 | 2.1 | 0.111 |
| **Total UPRORs (n)** | **6** | **51** | **0.007** |
| Number of patients with UPRORs [n (%)] | 5 (19) | 27 (38) | 0.443 |
| | | | |
| *EOSQ-24 Domains* | | | |
| **Pulmonary Function [mean (95% CI)]** | **93 (88-99)** | **79 (72-86)** | **0.017** |
| **Physical Function [mean (95% CI)]** | **48 (33-63)** | **25 (18-33)** | **0.003** |
| **Satisfaction [mean (95% CI)]** | **62 (52-72)** | **48 (41-54)** | **0.021** |

SD, standard deviation; BMI, body mass index; UPROR, unplanned return to operating room; CI, confidence interval

**Table 2.** Comparison of patients with distal spine anchors who did not and did undergo subsequent extension to the pelvis.

| *Demographics* | Distal spine anchors – no extension (N=21) | Distal spine anchors – extension to pelvis (N=6) | p-value |
|---|---|---|---|
| Age at index surgery (years; mean ± SD) | 7.1 ± 2.0 | 5.9 ± 2.8 | 0.236 |
| BMI at index surgery (kg/m$^2$; mean ± SD) | 15.8 ± 2.9 | 15.4 ± 2.6 | 0.757 |
| Nonambulatory status at index surgery [n (%)] | 15 (71) | 4 (67) | 0.686 |
| Length of follow-up (years; mean ± SD) | 5.5 ± 2.9 | 7.6 ± 4.1 | 0.157 |
| | | | |
| *Radiographic data* | | | |
| Pre-index major curve (°; mean ± SD) | 81 ± 25 | 68 ± 13 | 0.247 |
| Pre-index pelvic obliquity (°; mean ± SD) | 8 ± 5 | 4 ± 1 | 0.247 |
| **Pre-index L5 tilt (°; mean ± SD)** | **11 ± 6** | **19 ± 8** | **0.032** |
| Pre-index maximum sagittal deformity (°; mean ± SD) | 68 ± 22 | 48 ± 14 | 0.077 |
| Post-index major curve (°; mean ± SD) | 47 ± 20 | 48 ± 10 | 0.934 |
| Post-index pelvic obliquity (°; mean ± SD) | 8 ± 7 | 9 ± 5 | 0.696 |
| Post-index maximum sagittal deformity (°; mean ± SD) | 39 ± 18 | 38 ± 13 | 0.878 |
| Most recent major curve (°; mean ± SD) | 51 ± 21 | 33 ± 22 | 0.119 |
| Most recent maximum sagittal deformity (°; mean ± SD) | 54 ± 24 | 37 ± 8 | 0.207 |
| | | | |
| *Outcomes* | | | |
| **Total complications (n)** | **23** | **14** | **0.026** |
| **Number of patients with complications [n (%)]** | **14 (67)** | **6 (100)** | **0.031** |
| **Average complications per patients (n)** | **1.1** | **2.3** | **0.029** |
| Total UPRORs (n) | 4 | 2 | 0.518 |
| Number of patients with UPRORs [n (%)] | 3 (14) | 2 (33) | 0.320 |
| | | | |
| *EOSQ-24 Domains* | | | |
| **Physical Function [mean (95% CI)]** | **57 (39-75)** | **26 (9-44)** | **0.047** |

SD, standard deviation; BMI, body mass index; UPROR, unplanned return to operating room; CI, confidence interval

Figure 1. This DSA patient had a pre-index L5 tilt of 25° and underwent extension to the pelvis at the time of final fusion.

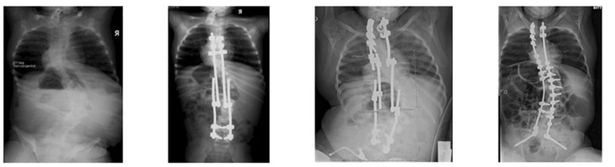

*Author Affiliations and Disclosures: Patrick J. Cahill, MD, The Children's Hospital of Philadelphia; NuVasive Inc (Consultant), Children's Spine Foundation (Grants/Research), Setting Scoliosis Straight Foundation (Grants/Research); Joel Gagnier, University of Michigan; Ying Li, MD, C.S. Mott Children's Hospital, Michigan Medicine, Scoliosis Research Society (Grants/Research), Medtronic, Inc. (Advisory Board or Panel); Robert F. Murphy, MD, Medical University of South Carolina; John T. Smith, MD, University of Utah; Paul D. Sponseller, MD, Johns Hopkins University; Pediatric Spine Study Group, Children's Spine Foundation; Jennylee Swallow, University of Michigan Medical School; Pediatric Spine Study Group, Pediatric Orthopaedic Society of North America (Grants/Research), FDA (Grants/Research), NuVasive (Grants/Research)*

### Paper # 16.

### Outcomes of magnetically controlled growing rods in severe early onset scoliosis. A matched comparison with traditional growing rods

*Antti J. Saarinen, Paul D. Sponseller, MD, Lindsay Andras, MD, David L. Skaggs, MD, John B. Emans, MD, George H. Thompson, MD, Ilkka J. Helenius, MD PhD Professor, Pediatric Spine Study Group*

**Summary**: Magnetically controlled growing rods (MCGR) provided similar deformity correction with less complications compared to traditional growing rods (TGR) in patients with severe early-onset scoliosis (EOS).

**Hypothesis**: We hypothesize that MCGR and TGR instrumentations present with similar deformity correction and spinal growth, and MCGR to have a reduced rate of complications in patients with severe EOS.

**Introduction**: TGRs are effective in controlling spinal deformity in EOS. However, repeated surgical lengthenings of TGRs expose patients to increased risk of complications. MCGRs allow outpatient based lengthenings and may reduce the risk of deep surgical site infection. In this study we compare MCGR and TGR in pediatric patients with severe (= 90°) EOS.

**Methods**: Inclusion criteria were age < 10 years at the time of surgery, severe EOS, = 2 years of follow-up, MCGR or TGR index procedure.

**Results**: The mean preoperative major curve was 102° in the MCGR (n = 29) and 105° in the TGR group (n = 28, p = 0.204). This decreased to 52° and 62° after the index operation (p = 0.009). At the final follow-up, the major curves were 52° and 63°, respectively (p = 0.011).

**Conclusion**: MCGR instrumentation provided similar deformity correction compared to the TGRs. Thoracic height improvement was slightly better in the TGR group. There were significantly fewer complications in the MCGR group. MCGR provides an effective and safe treatment option for children with severe EOS.

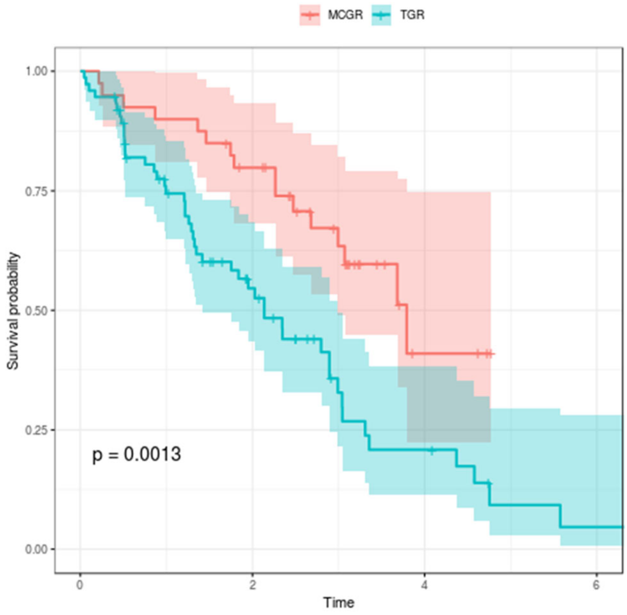

Kaplan-Meier analysis of instrumentation survival

| | MCGR (n=29) | TGR (n=28) | P-value |
|---|---|---|---|
| Age at surgery (years) | 7.1 (1.8 – 9.9) | 5.6 (1.9 – 9.9) | 0.007 |
| Female | 22 (76%) | 16 (58%) | 0.134 |
| Etiology: Neuromuscular | 18 (62%) | 9 (32%) | 0.024 |
| Syndromic | 3 (10%) | 9 (32%) | 0.044 |
| Idiopathic | 4 (14%) | 6 (21%) | 0.449 |
| Congenital | 4 (14%) | 4 (14%) | 0.957 |
| Follow-up (years) | 3.7 (2.2 – 5.6) | 8.6 (2.3 – 15) | <0.005 |
| Bilateral instrumentation | 24 (83%) | 19 (68%) | 0.191 |
| Major curve (°) Preoperative | 102 (90 –130) | 105 (90 – 139) | 0.204 |
| Index | 52 (21 – 85) | 62 (34 – 94) | 0.009 |
| 2-year follow-up | 52 (22 – 98) | 63 (23 – 103) | 0.011 |
| Thoracic height (mm), Preoperative | 151 (84 – 202) | 135 (80 – 203) | 0.027 |
| Index | 185 (138 –257) | 170 (105 – 301) | 0.082 |
| 2-year follow-up | 195 (151 – 289) | 186 (112 – 277) | 0.185 |
| Annual increase | 5.1 | 7.4 | 0.295 |
| Surgeries per year (range) | 0.46 (0.21 – 1.1) | 1.4 (0.51 – 2.7) | <0.005 |
| Lengthenings per year (range) | 2.8 (1.8 – 4.9) | 0.75 (0.29 – 1.9) | <0.005 |
| Unplanned surgeries per year (range) | 0.09 (0 – 0.83) | 0.09 (0 – 0.70) | 0.486 |

Patient characteristics.

| Complication | MCGR | TGR | P-value |
|---|---|---|---|
| Implant related | 15 | 43 | <0.005 |
| Rod fracture | 7 | 21 | <0.005 |
| Anchor pull out | 4 | 11 | 0.029 |
| Connector failure | 0 | 3 | 0.070 |
| Failure of distraction | 1 | 0 | 0.322 |
| Misplacement | 3 | 8 | 0.081 |
| Junctional kyphosis | 0 | 2 | 0.143 |
| Wound related | 4 | 15 | <0.005 |
| Superficial infection | 2 | 3 | 0.610 |
| Deep wound infection | 1 | 5 | 0.076 |
| Dehiscence | 1 | 7 | 0.019 |
| Neurologic deficit | 2 | 2 | 0.971 |
| Motor | 0 | 1 | 0.305 |
| Other | 2 | 1 | 0.574 |

Complications.

*Author Affiliations and Disclosures: Lindsay Andras, MD, Children's Hospital of Los Angeles; John B. Emans, MD, Boston Children's Hospital; Ilkka J. Helenius, MD PhD Professor, Turku University Hospital; Antti J. Saarinen, University of Turku; David L. Skaggs, MD, Children's Hospital of Los Angeles; Paul D. Sponseller, MD, Johns Hopkins University; Pediatric Spine Study Group, Children's Spine Foundation; George H. Thompson, MD, Rainbow Babies and Children's Hospital; Pediatric Spine Study Group, Pediatric Orthopaedic Society of North America (Grants/Research), FDA (Grants/Research), NuVasive (Grants/Research)*

### Paper # 17.

### Systematic investigation of metallosis associated with magnetically controlled growing rod implantation for early onset scoliosis

*Teng Zhang, PhD, Kam Yim Sze, ZW Peng, Kenneth MC Cheung, MD, YF Lui, Yat Wa Wong, Kenny Y H Kwan, Jason Pui Yin Cheung, MBBS, MMedSc, FRCS*

**Summary**: This was a prospective collection of metallosis and magnetically controlled growing rods (MCGRs) of 10 patients undergoing rod exchange. Titanium (Ti), Vanadium (V) and Neodymium (Nd) concentrations were increased. Black particles were present within the macrophages in the fibrotic tissues.

**Hypothesis**: For metallosis in MCGR surgery, we hypothesize that Nd should also be present in the soft tissues in addition to Ti and V. We expect a chronic inflammatory response with phagocytic foreign particles in immune cells.

**Introduction**: MCGRs have revolutionized surgery for early onset scoliosis. Complications especially metallosis (Fig. 1) is concerning as pseudo-capsule formations with black/grey particles are found. This study systematically investigates metallosis to reveal complete metal particle profile of the tissues surrounding the rod and the phagocytic immune response.

**Methods**: This was a prospective observational study of patients treated with MCGRs undergoing rod exchange. Metal-on-metal contact in the form of ring-like wear marks was found on the distracted portion of the piston immediately outside the barrel opening. Biopsies of paraspinal muscles and control tissue samples were taken. Spectrum analyses of the rod and biopsies were performed to reveal the metal components and concentrations.

**Results**: Ten patients were recruited. Ti, V and ND concentrations in the biopsies taken near the wear marks were found to be significantly higher than those in the control tissue samples (Fig. 2). Significantly increased Nd concentrations were also found in the tissues near the barrel of the MCGR. Chronic inflammation was revealed by the histological studies with fibrosis and macrophages infiltration. Black particles were present within the macrophages in the fibrotic tissues (Fig. 3).

**Conclusion**: Ti and V were generated mainly at the barrel opening due to metal-on-metal contact, whereas the Nd from the rotor of the MCGR is likely released from the barrel opening during distraction sessions. Phagocytotic immune cells with black particles inside raise cautions of the long-term implications of metallosis.

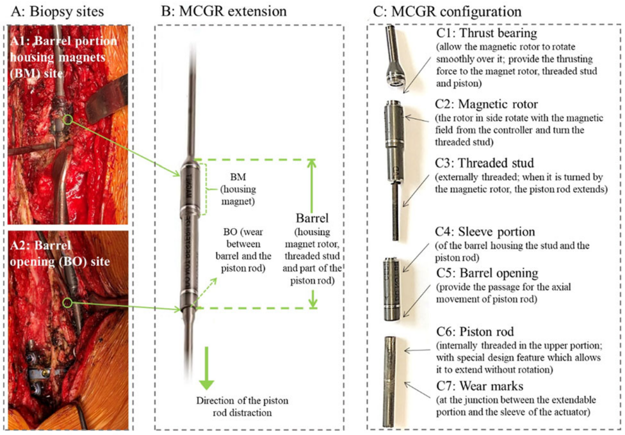

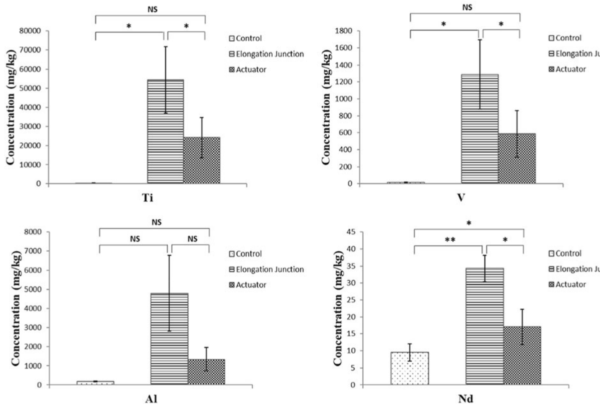

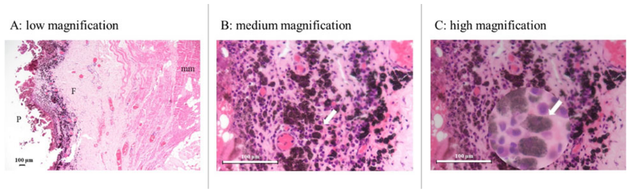

*Author Affiliations and Disclosures: Jason Pui Yin Cheung, MBBS, MMedSc, FRCS, The University of Hong Kong; Kenneth MC Cheung, MD, The University of Hong Kong; Kenny Y H Kwan, University of Hong Kong; YF Lui, University of Hong Kong; ZW Peng, University of Hong Kong; Kam Yim Sze, University of Hong Kong; Yat Wa Wong, University of Hong Kong; Teng Zhang, PhD, University of Hong Kong*

### Paper # 18

#### No difference in outcomes with high vs low implant density in growing construct conversion to posterior spinal fusion for early onset scoliosis

*Edward Compton, Purnendu Gupta, Jaime A. Gomez, MD, Kenneth Illingworth, David L. Skaggs, MD, Paul D. Sponseller, MD, Amer Samdani, Steven Hwang, Matthew Oetgen, MD MBA, Jennifer Schottler, George H. Thompson, MD, Michael G. Vitale, MD MPH, John T. Smith, MD, Lindsay Andras, MD, Pediatric Spine Study Group*

**Summary**: At 2-year follow-up of growth friendly graduates, curve correction and T1-S1 length gain were similar between high and low implant density (ID) constructs.

**Hypothesis**: Early onset scoliosis (EOS) patients treated with low density constructs will have similar outcomes as patients treated with high density constructs.

**Introduction**: Our purpose was to compare Early Onset Scoliosis (EOS) patients treated with low implant density to high density constructs when undergoing conversion to definitive fusion.

**Methods**: EOS patients treated with growth-friendly constructs converted to fusion between 2000 and 2017 reviewed from a multicenter database. ID was defined as number of pedicle screws, hooks, and sublaminar/bands per level fused. Patients were divided into low ID (< 1.6) and high ID (= 1.6). Exclusion criteria: < 2 years follow-up from fusion or inadequate radiographs.

**Results**: 152 patients met inclusion criteria with 39 (26%) patients in the high ID group and 113 (74%) patients in the low ID group. Groups were similar in age (p = 0.85), pre-fusion major curve (p = 0.45), number of levels fused (p = 0.34), operative time (p = 0.52), radiographic follow up (p = 0.73), and clinical follow up (p = 0.86). There was greater initial improvement in major curve in the high ID group (21.6o) than the low ID group (14.2°) (p = 0.01). During post-fusion follow-up, correction decreased 7.1o in the high ID group and 2.8 in the low ID group (p = 0.07). At final follow-up, major curve correction from pre-fusion was similar between groups (high ID:14.5° vs low ID:11.4°, p = 0.31). At final follow-up, there was no difference in T1-T12 length gain (p = 0.57), T1-S1 length gain (p = 0.65), coronal balance (p = 0.37) or sagittal balance (p = 0.87). There was no significant difference in revision rate between groups (high ID: 5.1% (2/39) and low ID: 9.7% (11/113)) (p = 0.52).

**Conclusion**: Although there was a slight trend towards increased complications in the low ID group, this study suggests that for many growth-friendly graduates, a low ID strategy produces similar outcomes with regards to curve correction and spinal length gain.

*Author Affiliations and Disclosures: Lindsay Andras, MD, Children's Hospital of Los Angeles; Edward Compton, Children's Hospital Los Angeles; Jaime A. Gomez, MD, Montefiore/Albert Einstein College of Medicine; Purnendu Gupta, Shriners Children's Hospital—Chicago; Steven Hwang, Shriners Hospitals for Children, Philadelphia; Kenneth Illingworth, Children's Hospital Los Angeles; Matthew Oetgen, MD MBA, Children's National Health System; Amer Samdani, Shriners Hospitals for Children, Philadelphia; Jennifer Schottler, Shriners Hospital for Children, Chicago; David L. Skaggs, MD, Children's Hospital of Los Angeles; John T. Smith, MD, University of*

*Utah; Paul D. Sponseller, MD, Johns Hopkins University; Pediatric Spine Study Group, Children's Spine Foundation; George H. Thompson, MD, Rainbow Babies and Children's Hospital; Michael G. Vitale, MD MPH, Columbia University Medical Center/Morgan Stanley Children's Hospital; Joshua Yang, Children's Hospital Los Angeles; Pediatric Spine Study Group, Pediatric Orthopaedic Society of North America (Grants/Research), FDA (Grants/Research), NuVasive (Grants/Research)*

### Paper # 19.

#### Where does vertebral growth occur during distraction by magnetically-controlled growing rods in patients with early onset scoliosis?

*Andy Yee, Kenneth MC Cheung, MD, Jianxiong Shen, Jason Pui Yin Cheung, MBBS, MMedSc, FRCS, Kenny Y H Kwan*

**Summary**: Distraction by magnetically-controlled growing rod (MCGR) can stimulate spinal growth but its effect on individual vertebral body is unknown. We showed that the highest percentage of total and annual vertebral height growth occurred in the vertebrae proximal to the distal instrumented vertebra (DIV−) but MCGR distraction had a negative effect on intervertebral discs development within the distracted levels.

**Hypothesis**: MCGR distraction accelerates longitudinal growth of the vertebral body in early-onset scoliosis (EOS).

**Introduction**: MCGR distraction has been shown to maintain spinal growth. The aim of this study was to investigate the effect of MCGR on vertebral body growth of the segments around the DIV in EOS patients.

**Methods**: EOS patients who underwent MCGR distractions with a minimum of 6 months distractions were included. Height, coronal width, and sagittal depth of vertebral body, and intervertebral spaces (IVS) height were measured at 2 segments proximal to the DIV (DIV−), DIV, and 2 segments distal to the DIV (DIV +) at baseline and final follow-up. The influence of MCGR on the vertebral growth were calculated.

**Results**: 21 EOS patients with a mean age at index surgery of 10 ± 3.2 years were included. Average duration of MCGR treatment was 3.7 years (range, 2–7 years). The total percentage growth of vertebral height was higher in DIV-group than DIV and DIV + group (23.3 ± 12.8% vs 16.3 ± 11.1% and 16.6 ± 9.1%). The DIV- group also exhibited the highest annual height growth compared with DIV and DIV + groups (7.5 ± 5.0% vs 5.8 ± 5.2% and 5.4 ± 3.9%). The total growth rates of width and depth demonstrated an increase from cranial to caudal vertebrae but did not reach statistical significance. All of DIV−, DIV and DIV + groups showed a reduction in IVS. The reduction in IVS in the DIV group demonstrated the greatest reduction in total and annual growth when compared to DIV + group (− 10.9 ± 6.0% vs − 2.0 ± 7.2%, p = 0.001).

**Conclusion**: The DIV- group had the highest total growth and fastest annual growth of vertebral height. These main findings indicated that distraction force from MCGR was likely to stimulate the longitudinal growth of individual vertebral body.

*Author Affiliations and Disclosures: Jason Pui Yin Cheung, MBBS, MMedSc, FRCS, The University of Hong Kong; Kenneth MC Cheung, MD, The University of Hong Kong; Kenny Y H Kwan, University of Hong Kong; Jianxiong Shen, Peking Union Medical College Hospital; Andy Yee, Queen Mary Hospital*

### Paper # 20.

#### The association between the utilization of traction and postoperative complications following growing rod instrumentation

*Benjamin D. Roye, MD-MPH, Michael Fields, Hiroko Matsumoto, PhD, Paul D. Sponseller, MD, Francisco Javier Sánchez Pérez Grueso, Oheneba Boachie-Adjei, MD, Kim Hammerberg, MD, Michelle Welborn, MD, Michael G. Vitale, MD MPH, Pediatric Spine Study Group*

**Summary**: This multicenter study with large sample size provides the best evidence to date for the efficacy of traction in correcting spinal deformities and preventing hardware associated complications in patients with early onset scoliosis.

**Hypothesis**: Patients who receive preoperative and/or intraoperative traction prior to implantation of a growing rod will have a lower risk of complications compared to those that do not undergo traction at 2 years follow-up.

**Introduction**: The purpose of this multicenter study was to investigate the association between preoperative/intraoperative traction and complications following growth friendly instrumentation for EOS.

**Methods**: Patients with EOS who underwent growth rod instrumentation prior to 2017 were identified from two registries. Patients were divided into two groups: preoperative traction group vs. no preoperative traction group. A subgroup analysis was done to compare intraoperative traction only vs no traction (Figure). Data was collected on any postoperative complication from implantation to up to two years post implantation.

**Results**: Of 381 patients identified, 57 (15%) and 69 (18%) patients received preoperative and intraoperative traction, respectively. After adjusting for etiology and degree of kyphosis, there was no evidence to suggest that preoperative halo traction reduced the risk of any complication following surgical intervention. Although not statistically significant, a sub-group analysis of patients with severe curves demonstrated a trend toward a markedly reduced hardware failure rate in patients undergoing preoperative halo traction (preop traction: 1 (3.1%) vs. no preop traction: 11 (14.7%), p = 0.083). Non-idiopathic, hyper-kyphotic patients treated with intraoperative traction were 61% less likely to experience any postop complication (p = 0.067) and were 74% (p = 0.091) less likely to experience UPROR when compared to patients treated without traction (Table).

**Conclusion**: Intraoperative traction may play an important role in preventing hardware associated complications and unplanned return to the operating room in patients with EOS, especially those with non-idiopathic etiology and hyper-kyphosis.

Figure 1. Patient groups and statistical analyses performed.

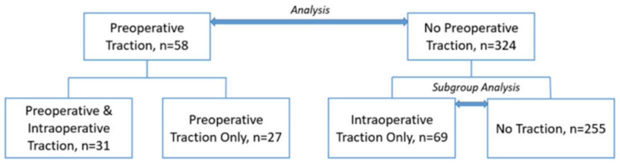

Table. Intraoperative traction vs. no traction radiographic outcomes and complications at two year follow up.

| | No Traction (n=69, 21.3%) | Intraoperative Traction (n=255, 78.7%) | P-Value |
|---|---|---|---|
| | *Demographics* | | |
| *Gender* | | | |
| Female | 131 (51.4%) | 43 (62.3%) | |
| Male | 124 (48.6%) | 26 (37.7%) | 0.106 |
| *Prior Treatment* | | | |
| None | 192 (75.3%) | 36 (52.2%) | |
| Brace | 43 (16.9%) | 26 (37.7%) | |
| Cast | 2 (0.8%) | 1 (1.4%) | |
| Brace and Cast | 0 (0%) | 6 (8.7%) | |
| Previous Surgery | 18 (7.1%) | 0 (0%) | <0.001 |
| *Etiology* | | | |
| Idiopathic | 38 (15%) | 13 (19.4%) | |
| Congenital | 64 (25.3%) | 6 (9%) | |
| Neuromuscular | 101 (39.9%) | 28 (41.8%) | |
| Syndromic | 50 (19.8%) | 20 (29.9%) | 0.022 |
| *Instrumentation Type* | | | |
| MCGR | 34 (13.3%) | 13 (18.8%) | <0.001 |
| Soine | 14 (41.2%) | 13 (100%) | |
| Rib | 20 (58.8%) | 0 (0%) | |
| VEPTR | 109 (42.7%) | 2 (2.9%) | |
| TGR | 112 (43.9%) | 54 (78.3%) | |
| | *Radiographic Parameters Mean±SD (Min, Max)* | | |
| Preop Primary Curve | 71.2±21.2 (10.0,129.0) | 79.1±17.0 (39.8,125.0) | 0.005 |
| 2 Year Primary Curve | 48.0±18.4 (12.0,115.0) | 44.6±14.8 (20.0,72.0) | 0.343 |
| Primary Curve % Correction | 31.6±28.0 (-88.2,87.4) | 43.8±17.7 (14.6,73.7) | 0.023 |
| Preop Compensatory Curve | 39.8±17.4 (7.0,97.0) | 44.8±18.8 (8.0,115.0) | 0.078 |
| 2 Year Compensatory Curve | 33.7±17.1 (3.0,102.0) | 33.7±14.9 (14.0,65.0) | 0.999 |
| Preop Global Kyphosis | 46.6±30.3 (-41.0,180.0) | 52.1±18.3 (17.0,85.0) | 0.186 |
| 2 Year Global Kyphosis | 44.3±19.5 (6.0,125.0) | 38.6±17.3 (3.0,72.0) | 0.176 |
| Preop Lordosis | -48.9±20.0 (-126.0,0.0) | 7.5±55.0 (-96.0,100.0) | <0.001 |
| 2 Year Lordosis | 38.9±33.3 (-76.0,82.0) | 18.5±50.1 (-85.0,87.0) | 0.011 |
| | *Complications n (%)* | | |
| Any Complication | 123 (48.2%) | 25 (36.2%) | 0.076 |
| Hardware Failure | 30 (11.8%) | 1 (1.4%) | 0.010 |
| Anchor Migration | 52 (20.4%) | 1 (1.4%) | <0.001 |
| Prominence/Protrusion | 8 (3.1%) | 3 (4.3%) | 0.622 |
| SSI | 17 (6.7%) | 2 (2.9%) | 0.237 |
| Other Wound Related | 14 (5.5%) | 2 (2.9%) | 0.378 |
| PJK | 2 (0.8%) | 2 (2.9%) | 0.158 |
| Progression | 3 (1.2%) | 2 (2.9%) | 0.303 |
| Neuro | 6 (2.4%) | 3 (4.3%) | 0.371 |
| Pain | 9 (3.5%) | 0 (0%) | 0.113 |
| Medical | 15 (5.9%) | 3 (4.3%) | 0.622 |
| UPROR | 53 (20.8%) | 4 (5.8%) | 0.004 |

*Author Affiliations and Disclosures: Hiroko Matsumoto, PhD, Columbia University; Benjamin D. Roye, MD-MPH, Columbia University; Francisco Javier Sánchez Pérez Grueso, Hospital La Paz; Paul D. Sponseller, MD, Johns Hopkins University; Pediatric Spine Study Group, Children's Spine Foundation; Michael G. Vitale, MD MPH, Columbia University Medical Center/Morgan Stanley Children's Hospital; Michelle Welborn, MD, Shriners Hospital, Depuy (Speaker's Bureau), K2M (Speaker's Bureau), NuVasive (Speaker's Bureau, Grants/Research); Pediatric Spine Study Group, Pediatric Orthopaedic Society of North America (Grants/Research), FDA (Grants/Research), NuVasive (Grants/Research)*

### Paper # 21.

#### Liposomal bupivacaine infiltration reduces opioid consumption after MCGR implantation

*Amy McIntosh, MD, Chris McLoed, David C. Thornberg*

**Summary**: Post-operative multi-modal pain management after MCGR insertion often involves the combination of both narcotic and non-narcotic medications. This cohort controlled case series highlights local infiltration anesthesia (LIA) with liposomal bupivacaine (exparel) to enhance post-operative pain control after insertion of MCGR. The exparel cohort consumed statistically significantly less morphine equivalents through all time points compared to the control cohort.

**Hypothesis**: We hypothesized that the Exparel cohort would have equivalent post-operative consumption of morphine equivalents compared to the control cohort.

**Introduction**: A multimodal approach to pain control occurs when several non-opioid analgesic medications are given in combination with opioid medication. The goal of these post-operative pathways is minimize narcotic usage. Exparel is a liposome encapsulated local anesthetic that has a duration of action up to 72 h. We compared MCGR insertion patients that received LIA with exparel to a group that received opioid based pain control.

**Methods**: We compared 2 cohorts of MCGR insertion patients. The exparel cohort had LIA of exparel, mixed with plain bupivacaine and normal saline, injected into the fascial and subcutaneous layers of the incision prior to closure. Otherwise, cohorts received the same multimodal post-operative pain protocol consisting of opioids, acetaminophen, ibuprofen and diazepam.

**Results**: The exparel cohort (n = 5), average age of 5.1 years at the time of MCGR implantation was compared to a control group (n = 6), average age 7.1 years. Both cohorts received standardized dosing consisting of an IV opioid with transition to oral opioid as tolerated. The exparel cohort consumed less morphine equivalents at all time points compared to the control group. (0–24 h: 6.6 mg vs 10.67 mg), (24–48 h: 8.4 mg vs. 16.17 mg), and (48–72 h: 5.4 mg vs. 14.5 mg) ($p = 0.047$).

**Conclusion**: LAI with exparel into the fascial and subcutaneous layers prior to closure was associated with less consumption of morphine equivalents in MCGR insertion patients at all time points in the acute post-operative period.

*Author Affiliations and Disclosures: Amy McIntosh, MD, Texas Scottish Rite Hospital for Children, NuVasive (Consultant); Chris McLoed, Texas Scottish Rite Hospital; David C. Thornberg, Texas Scottish Rite Hospital for Children*

### Paper # 22.

#### Matched comparison of growing rods versus primary posterior spinal fusion in "Tweeners" with early onset scoliosis

*Lukas G. Keil, MD, Alysa B. Nash, Til Stürmer, Yvonne M. Golightly, Feng-Chang Lin, Joseph D. Stone, James O. Sanders, MD, Craig R. Louer*

**Summary**: In 7 to 11-year-old "tweeners" with EOS in whom bracing fails, the optimal surgical option remains uncertain. We conducted a retrospective comparative study to compare growing rods (GRs) followed by definitive posterior spinal fusion (PSF) versus primary PSF with regard to thoracic height increase (surrogate of thoracic cavity size), deformity correction, complications, and total number of operations. Twenty-five GR patients were matched n:1 with Hypothesisment to 17 PSF patients selected from a cohort of 60 PSFs. Matching criteria included etiology (idiopathic, neuromuscular, syndromic, thoracogenic, or congenital), Cobb angle, and adjusted skeletal age at index surgery. All patients were successfully matched for etiology. Matches had mean delta Cobb of 1° and mean delta adjusted age at index of 0.5 years (GRs older than PSFs). Analysis of matched pairs demonstrated mean 2.1 cm greater overall T1-12H increase among GRs than their matched PSFs; mean 22% less overall coronal deformity correction among GRs than their matched PSFs, and mean 1 additional complication and 2 additional operations per GR patient. All of these differences were statistically significant. GRs remain a viable option for severe EOS in very young patients. However, in many "tweeners" aged 7–11 the ∼ 2 cm of thoracic height gained over primary PSF may not warrant the average ∼ 22% loss of deformity correction and additional 1 complication and 2 operations per patient. Surgeons and families should weigh these concerns when choosing a treatment plan.

**Hypothesis**: We sought to compare growing rods (GRs) followed by definitive posterior spinal fusion (PSF) versus primary PSF in patients aged 7–11 years with early onset scoliosis (EOS). We hypothesized that the incremental benefit of increased thoracic height afforded by GRs would be offset by increased rigidity (decreased deformity correction), more complications, and more total operations.

**Introduction**: For patients with early onset scoliosis (EOS) in whom bracing fails, all surgical options have significant drawbacks. Traditional and magnetically-controlled growing rods (TGRs and MCGRs) afford some correction and avoidance of progression while allowing further growth prior to definitive posterior spinal fusion (PSF). Unfortunately, their use causes iatrogenic deformity stiffness and is fraught with numerous, often severe complications. Similarly, primary PSF in the skeletally immature patient is not without morbidity. In 7 to 11-year-old "tweeners," the optimal surgical option remains uncertain.

**Methods**: This retrospective comparative study included EOS patients aged 7.0–11.9 years at index surgery treated with GRs followed by PSF (or explantation) or with primary PSF. Primary outcomes were thoracic height (T1-12H) and coronal deformity (Cobb angle). Outcomes in the primary PSF group were measured (1) pre-PSF and (2) post-PSF. In the GR group they were measured (1) pre-GR, (2) post-GR implantation, (3) post-growth phase (GP)/pre-PSF, and (4) post-PSF. Secondary outcomes were complications and total operations. GR patients were manually matched with Hypothesisment n:1 to PSF patients by age at index (adjusted for skeletal age if abnormal), etiology (idiopathic, neuromuscular, syndromic, thoracogenic, or congenital), and Cobb angle. No PSF match was used more than three times to minimize undue influence of any one patient. Data were analyzed using (1) the Wilcoxon signed rank test adjusted for dependency due to n:1 matching and (2) a linear mixed effects model adjusted for age difference between matched pairs.

**Results**: Twenty-eight patients treated with GRs (19 with MCGRs, 9 with TGRs) met criteria and were included. Three MCGRs were definitively explanted without PSF due to complications and were included but not matched, as post-PSF measurements could not be compared. For the 25 remaining GRs, we identified 17 primary PSF matches from a cohort of 60 PSFs. Four MCGRs have PSF scheduled for summer 2020; these were matched but excluded from pre- and post-PSF measurements. All patients were successfully matched for etiology. Matches had mean delta Cobb of 1° (p = 0.784) and mean delta adjusted age at index of 0.5 years (GRs older than PSFs, p = 0.025, Table 1). Among 28 GRs, median age at index was 10.0 years (IQR 2.0, range 7.0–11.9) with median T1-12H of 18.4 cm (IQR 3.0, range 14.4–23.6) and median Cobb of 73° (IQR 17, range 52–103). Median coronal deformity correction at GR implantation was 45% (IQR 29) with median 1.6 cm increase in T1-12H (IQR 1.2). During GP (median 33 months, IQR 21) T1-12H increased by median 0.2 cm (IQR 3.4, range − 2.8 to 3.6). Coronal deformity recurrence by median 35% of pre-GR curve (IQR 28%) was not fully corrected at PSF for median net deformity correction pre-GR to post-PSF of 36% (IQR 38%). Median increase in T1-12H pre-GR to post-PSF was 4.0 cm (IQR 2.9). GR patients had median 2 complications (IQR 1, range 0–6) and required median 2 operations (IQR 3, range 2–7). Among 52 complications the most common were instrumentation migration/failure (20), junctional kyphosis or severe curve progression (8), wound/skin breakdown or infection (8), and pneumonia/respiratory failure (11). Sixteen complications required aborting/truncating GR treatment. Among 17 PSFs, median age at index was 10.6 years (IQR 1.7, range 7.8–11.8) with median T1-12H of 20.1 cm (IQR 4.4, range 15.0–24.8) and median Cobb of 71° (IQR 17, range 38–108). Median deformity correction at PSF was 62% (IQR 30%) with median 1.7 cm increase in T1-12H (IQR 1.5). PSF patients had median 0 complications (IQR 1, range 0–4) and required median 1 operation (IQR 0, range 1–3). Among 10 complications the most common were junctional kyphosis or severe curve progression (3) and infection (3). Analysis of matched pairs demonstrated mean 2.1 cm greater overall T1-12H increase among GRs than their matched PSFs (p = 0.004); mean 22% less overall coronal deformity correction among GRs than their matched PSFs (p = 0.021), and mean 1 additional complication and 2 additional operations per GR patient (p = 0.038 and p = 0.004, respectively, Table 1).

**Conclusion**: GRs remain a viable option for severe EOS in very young patients. However, in many "tweeners" aged 7 to 11 the ∼ 2 cm of thoracic height gained over primary PSF may not warrant the average ∼ 22% loss of deformity correction and additional 1 complication and 2 operations per patient. Surgeons and families should weigh these concerns when choosing a treatment plan.

| | GRs → PSF (n=28) | Primary PSF (n=17) | Mean Difference Between Matched Pairs (n=25) | p-value† | p-value‡ |
|---|---|---|---|---|---|
| Age at index, years | 10.0 (2.0) | 10.6 (1.7) | 0.5 years* (GRs younger) | **0.025** | N/A |
| Thoracic height at index, cm | 18.4 (3.0) | 20.1 (4.4) | 1.0 cm (GRs shorter) | 0.173 | 0.277 |
| Cobb angle at index, ° | 73° (17°) | 71° (17°) | 1° (GRs smaller) | 0.784 | 0.773 |
| Thoracic height increase, cm | | | | | |
| GR Implantation | 1.6 (1.2) | | | | |
| Growth phase | 0.2 (3.4) | | | | |
| Overall (pre-index to post-PSF) | 4.0 (2.9) | 1.7 (1.5) | 2.1 (GRs greater) | **0.004** | **0.010** |
| Cobb angle % correction | | | | | |
| GR Implantation | 45% (29%) | | | | |
| Growth phase§ | -35% (28%) | | | | |
| Overall (pre-index to post-PSF) | 36% (38%) | 62% (30%) | 22% (GRs less) | **0.021** | **0.010** |
| Complications per patient, mean (SD) | 2 (1) | 0 (1) | 1 (GRs more) | **0.038** | **0.031** |
| Operations per patient, mean (SD) | 2 (3) | 1 (0) | 2 (GRs more) | **0.004** | **<0.001** |

*Adjusted for skeletal age if abnormal
†Wilcoxon signed rank test of matched pairs, adjusted for dependency introduced by n:1 matching (Rosner et al., 2006)
‡Linear mixed effects model with random intercept, adjusted for age difference between matched pairs
§Recurrence of deformity

Table 1. Comparison of growing rods (GRs) followed by definitive posterior spinal fusion (PSF) versus primary PSF, values expressed as median (IQR) unless otherwise specified

*Author Affiliations and Disclosures: Yvonne M. Golightly, Gillings School of Global Public Health, UNC, Chapel Hill, NC; Lukas G. Keil, MD, Department of Orthopaedic Surgery, University of North Carolina, Chapel Hill, NC; Feng-Chang Lin, Gillings School of Global Public Health, UNC, Chapel Hill, NC; Craig R. Louer, Department of Orthopaedic Surgery, University of North Carolina, Chapel Hill, NC; Alysa B. Nash, Department of Orthopaedic Surgery, University of North Carolina, Chapel Hill, NC; James O. Sanders, MD, University of North Carolina at Chapel Hill; Joseph D. Stone, Department of Orthopaedic Surgery, University of North Carolina, Chapel Hill, NC; Til Stürmer, Gillings School of Global Public Health, UNC, Chapel Hill, NC*

### Paper # 23.

### Is growth-friendly surgery adequate for the treatment of non-ambulatory early-onset scoliosis myelomeningocele patients?

*Norman Ramirez-Lluch, MD, Ryan E. Fitzgerald, Gerardo Olivella, John T. Smith, MD, Peter F. Sturm, MD, Paul D. Sponseller, MD, Lawrence I. Karlin, MD, Scott J. Luhmann, MD, Tricia St. Hilaire, MPH, Pediatric Spine Study Group*

**Summary**: Growth Friendly Surgery (GFS) in Non Ambulatory-Early Onset Scoliosis (NA-EOS) myelomeningocele patients has a potential role to detain spine deformity and increase T1-S1 spine height.

**Hypothesis**: Evaluate surgical outcomes and complications of NA-EOS myelomeningocele patients treated with GFS techniques.

**Introduction**: Non-ambulatory myelomeningocele patients tend to develop early-onset progressive spinal deformity affecting their overall health. Their surgical correction options are associated with high complication rate. Purpose of this study is to determine surgical outcomes and complications of NA-EOS myelomeningocele patients treated with GFS techniques.

**Methods**: This is a multicenter retrospective IRB approved study of all NA-EOS myelomeningocele pts treated with GFS techniques from 2004 to 2017 with at least 3-lengthening procedures was conducted. Demographic and radiographic data was evaluated at pre, immediate and most-recent post-op period.

**Results**: Thirty-seven NA-EOS myelomeningocele pts underwent GFS technique with an average age of 5.5 + 2.5 yrs. Mean EBL 58 mL and post-op length stay 4.1 + 1.9 days. Follow-up was 4.6 + 2.1 yrs. Average # of surgical lengthening procedures was 7.0 + 3.0. Average preop coronal Cobb angle was 67.7? + 33.0? with postop angle of 41.4? + 20.5?, and 60.5? + 28.1? angle at most recent follow-up. Mean preop sagittal Cobb angle showed 51.0? + 45.5? with postop of 35.2? + 35.1? angle and 52.5? + 22.5? angle at most recent FU. Fifteen-% of spine height T1-S1 increase was shown after first surgical procedure with continuous growth of 1-cm by yr. Seventy-one complications (60-surgeries and 11-non-operative medical interventions) occurred in 29/37 (78.4%) pts with an average of 2.4- complication per pt. Complication risk after initial implantation was 24%, increasing 6% with each lengthening procedure. Most common complications were device related (76%), infection (14.1%), pain (5.6%), pneumothorax (1.4%) and disease related death (2.8%).

**Conclusion**: Substantial complication rate was reported in NA-EOS myelomeningocele pts. However, use of GFS technique in this population has a potential role to detain spine deformity and increase T1-S1 spine height.

*Author Affiliations and Disclosures: Ryan E. Fitzgerald, Riley Hospital for Children; Lawrence I. Karlin, MD, Children's Hospital Boston; Scott J. Luhmann, MD, Washington University School of Medicine; Gerardo Olivella, Department of Orthopedic Surgery, University of Puerto Rico; Norman Ramirez-Lluch, MD, Hospital de la Concepcion—San German; John T. Smith, MD, University of Utah; Paul D. Sponseller, MD, Johns Hopkins University; Tricia St. Hilaire, MPH, Children's Spine Foundation; Pediatric Spine Study Group, Children's Spine Foundation; Peter F. Sturm, MD, Cincinnati Children's Hospital Medical Center; Pediatric Spine Study Group, Pediatric Orthopaedic Society of North America (Grants/Research), FDA (Grants/Research), NuVasive (Grants/Research)*

### Paper # 24.

### Half of MCGR "stall" 3 years after implantation

*Amy McIntosh, MD, Charles E. Johnston, MD, Brandon A. Ramo, MD, Anna M. McClung, Researcher, David C. Thornberg*

**Summary**: Half (27/57) 47% of all MCGR implanted and followed at a single institution had stalling confirmed by x-ray measurements taken in an EOS machine by 3.3 years post-operatively.

**Hypothesis**: Increasing time since implantation leads to increasing stall rates for MCGR.

**Introduction**: MCGR patients with instrumentation implanted at our institution who had undergone least one lengthening were included. Age at placement, preop cobb, preop diagnosis, lengthening duration, and total attempted lengthenings were compared.

**Methods**: MCGR patients with instrumentation implanted at our institution who had undergone least one lengthening were included. Age at placement, preop cobb, preop diagnosis, lengthening duration, and total attempted lengthenings were compared.

**Results**: 10 syndromic, 12 congenital, 16 EOIS, 19 NM were included (n = 57). 6 were converted from TGR to MCGR (mean 6.8 yrs) while 51 had index placement of MCGR (mean 6.5 yrs). Mean preop cobb was 79° (47°–128°) in the 51 patients. 27/57 (47%) had suspected/definite rod stalling while 30/57 (53%) had no indication of stalling. Patients with stalling had been treated longer (3.3 vs 2.3 yr, p = 0.003) with more attempted lengthenings (9.3 vs 6.5 attempts, p = 0.009). There were no differences between age at insertion (6.4 vs 6.6 yr, p = 0.7), preop cobb (81° vs 77°, p = 0.7), or underlying diagnosis (50%, 50%, 47%, 40%). 11/57 (19%) had their MGR removed. 8 of those patients went on to definitive fusion, 2 patients were converted to TGR, and 1 patient had a new MCGR placed.

**Conclusion**: Half (27/57) 47% of MCGR "stall" after an average of 9 attempted lengthenings and by an average of 3.3 years post implantation.

| Variable | 0 No Stalling [N=30] | 1 Stalling [N=27] | p-value |
| --- | --- | --- | --- |
| Age [Normal] | 6.6 yr | 6.4 yr | 0.696 |
| Duration [Normal] | 2.3 yr | 3.3 yr | 0.003 |
| Number of Lengthenings [Not] | 6.5 lengthenings | 9.3 lengthenings | 0.009 |
| PreOp Cobb [N=49] [0=27 1=22] [Not] | 77 degrees | 81 degrees | 0.658 |

*Author Affiliations and Disclosures: Charles E. Johnston, MD, Texas Scottish Rite Hospital; Anna M. McClung, Researcher, Texas Scottish Rite Hospital for Children; Amy McIntosh, MD, Texas Scottish Rite Hospital for Children, NuVasive (Consultant); Brandon A. Ramo, MD, Texas Scottish Rite Hospital for Children; David C. Thornberg, Texas Scottish Rite Hospital for Children*

### Paper # 25.

### Scoliosis flexibility correlate with post-operative outcomes following growth friendly surgery

*Riley J. Bowker, Kevin Morash, MD, Burt Yaszay, MD, Lindsay Andras, MD, Peter F. Sturm, MD, Paul D. Sponseller, MD, George H. Thompson, MD, Pediatric Spine Study Group, Ron El-Hawary, MD*

**Summary**: As lower pre-operative flexibility was associated with less post-operative scoliosis correction and pre-operative flexibility < 30% was associated with a higher risk of post-operative complications, curve flexibility should be considered when deciding upon the timing of growth friendly surgery.

**Hypothesis**: For EOS patients who have received growth friendly surgery (GFS), lower pre-op flexibility will result in decreased scoliosis correction and a higher risk of post-op complications.

**Introduction**: There has been insufficient study of the relationship between pre-op flexibility and post-op outcomes for EOS patients who receive GFS.

**Methods**: EOS patients with pre-op flexibility x-rays (traction or bend) were identified. Pre-op % flexibility and immediate post-op % correction were calculated for each patient. Complications were recorded. Pearson correlations were determined for % flexibility vs % correction for all patients and were compared between etiologies and between device types (MCGR, TGR, VEPTR).

**Results**: 107 patients (14 congenital, 43 NM, 31 syndromic, 19 idiopathic) with mean age 7.1 yr at index surgery were identified. Pre-op scoliosis was 77o. Mean flexibility of 36% did not differ between etiologies. Immediate post-op scoliosis was 46o* with mean correction of 38%. Percent correction did not differ by etiology (Table 1), but did differ between device type (MCGR 45%, TGR 40%, VEPTR 14% *; Table 2). Pearson correlation for preoperative % flexibility vs % correction was fair (r = 0.37*). This correlation was observed for idiopathic (r = 0.53*) and NM (r = 0.46*), but not for congenital or syndromic. At a mean of 4.8 yr f/u, 66 patients (62%) experienced at least one complication. Risk ratio for developing a complication was

1.58 (1.18–2.11) for patients with pre-op flexibility < 30%*. *denotes p < 0.05.

**Conclusion**: As lower pre-operative flexibility was associated with less post-operative scoliosis correction and pre-operative flexibility < 30% was associated with a higher risk of post-operative complications, curve flexibility should be considered when deciding upon the timing of growth friendly surgery.

| Etiology | n | Mean Flexibility | Mean Correction | Correlation | p Value |
|---|---|---|---|---|---|
| Idiopathic | 19 | 34% | 37% | 0.53 | <0.05 |
| Congenital | 14 | 30% | 37% | 0.13 | 0.67 |
| Neuromuscular | 43 | 38% | 39% | 0.46 | <0.01 |
| Syndromic | 31 | 37% | 38% | 0.24 | 0.20 |

| Device | n | Mean Flexibility | Mean Correction | Correlation | p Value |
|---|---|---|---|---|---|
| MCGR | 39 | 40% | 45% | 0.60 | <0.0001 |
| TGR | 54 | 35% | 40% | 0.12 | 0.38 |
| VEPTR | 14 | 29% | 14% | 0.02 | 0.95 |

*Author Affiliations and Disclosures: Lindsay Andras, MD, Children's Hospital of Los Angeles; Riley J. Bowker, IWK Health Centre; Ron El-Hawary, MD, IWK Health Centre; Depuy Synthes (Grants/Research), Medtronic (Grants/Research), Orthopediatrics (Consultant, Advisory Board or Panel, Stock/Shareholder (self-managed); Kevin Morash, MD, IWK Health Centre; Paul D. Sponseller, MD, Johns Hopkins University; Pediatric Spine Study Group, Children's Spine Foundation; Peter F. Sturm, MD, Cincinnati Children's Hospital Medical Center; George H. Thompson, MD, Rainbow Babies & Children's Hospital; Burt Yaszay, MD, Rady Children's Hospital—San Diego; Pediatric Spine Study Group, Pediatric Orthopaedic Society of North America (Grants/Research), FDA (Grants/Research), NuVasive (Grants/Research)*

### Paper # 26.

### Collagen X Biomarker (CXM) is predictive of growth cessation in Idiopathic Scoliosis

*Michelle Welborn, MD, Ryan Coghlan, James O. Sanders, MD, Vishwas R. Talwalkar, Amer Samdani, MD, Joseph Stone, MD, Daniel Bouton, MD, William Horton, Susan Sienko*

**Summary**: Assessment of the cessation of growth is important for determining brace duration for scoliosis. Sanders stage and Risser score poorly predict growth cessation. CXM levels appear to predict growth cessation.

**Hypothesis**: In this study we sought to evaluate if SS7 patients still had measureable growth and if CXM levels were predictive of cessation of growth.

**Introduction**: Assessment of cessation of growth for is critical for determining surgical and brace treatment of scoliosis patients. Risser score (RS) was the gold standard but it has been shown that it can result in the mistreatment of 1/4 braced patients with AIS. Additional studies showed over 20% of Sanders stage (SS) 7 pts had curve progression at cessation of bracing. Type X collagen (COLX) is produced in the growing physis during enchondral ossification. CXM is a breakdown product of COLX. CXM, thus, is a direct measure of enchondral ossification and longitudinal bone growth.

**Methods**: Q6mo anthropometrics and spine PA biplanar slot scanner images including the hand were assessed for major curve magnitude, RS, triradiate cartilage status (TRC), Greulich and Pyle bone age (BA), and SS. Serial Dried Blood Spots (DBS) to obtain CXM levels were collected 3 consecutive days Q1-2 months based on SS.

**Results**: During the 2.5 year period of the study 47 patients with idiopathic scoliosis, Cobb = 20 were enrolled. Only SS7 patients were included in this subanalysis, 9 patients became SS7 during that period. CXM levels were assayed in quadruplicate for a total of 52 samples. CXM results were highly reproducible with an intraassay coefficient of variation of 3%, and interassay of 12%. No patient with a CXM level < 5 ng/ml had remaining growth.

**Conclusion**: CXM is the first biomarker specific to longitudinal bone growth. We previously established that it is a patient-specific, real time measure of growth velocity with high correlation to anthropometric and radiographic measures of growth. In this study we found that all SS7 patients with a CXM > 5 ng/ml had remaining growth and none of the patients < 5 ng/ml had remaining growth.

| Subject # | Gender | Height Velocity (cm/yr) | Closest CXM (ng/ml) to visit | Mean CXM (ng/ml) between visits |
|---|---|---|---|---|
| 1003 | F | 1.6 | 9.56 | 9.5 |
| | | .60 | 6.65 | 7.24 |
| | | .50 | 5.92 | 6.47 |
| 1014 | F | 4.0 | 6.85 | 6.66 |
| | | 0.0 | 4.43 | 5.74 |
| 1015 | M | 4.88 | 15.68 | 18.67 |
| 1021 | F | 3.2 | 3.26 | 6.75 |
| 1022 | F | 2.06 | 9.22 | 10.06 |
| 1024 | M | 7.04 | 16.31 | 24.55 |
| 1027 | F | 3.40 | 9.35 | 10.97 |
| | | .60 | 6.51 | 7.61 |
| 1036 | F | 2.01 | 6.11 | 9.06 |
| 1039 | F | 2.00 | 11.00 | 10.53 |

Table: Adolescents in SS 7, height velocity, CXM closest to visit and the mean CXM between visits

*Author Affiliations and Disclosures: Daniel Bouton, MD, Shriners Hospital for Children – Portland, Medtronic (Consultant); Ryan Coghlan, Shriners Hospital for Children Portland; William Horton, Shriners Hospital for Children Portland; Amer Samdani, MD, Shriners Hospital Outpatient Clinic; James O. Sanders, MD, University of North Carolina at Chapel Hill; Susan Sienko, Shriners Hospital for Children Portland; Joseph Stone, MD, Pediatric Orthopedic Associates; Vishwas R. Talwalkar, Shriners Hospital for Children; Michelle Welborn, MD, Shriners Hospital, Depuy (Speaker's Bureau), K2M (Speaker's Bureau), NuVasive (Speaker's Bureau, Grants/Research)*

### Paper # 27.

### Fetal spinal anomalies—incidence and future perspectives—retrospective study of 10,000 scans

*Hriday P. Acharya, Abhay Nene, Prashant Acharya*

**Summary**: Prenatal diagnosis of spinal anomalies is now a well established concept in the developed world. Looking for spinal anomalies is now an integral part of most prenatal ultrasound evaluations worldwide. Here we look into how big this spectrum of disease is; which anomalies can commonly be identified and how often are they identified at a tertiary care center.We try to look into the fact that such anomlies can be followed up after birth and help in early diagnosis and proper management.

**Hypothesis**: Diagnosing anomalies make the basis of treatment in any patient with EOS. If we follow up such patient even before birth, an early treatment can give us a lead time in avoiding adverse events.

**Introduction**: Prenatal diagnosis of spinal anomalies is now a well established concept in the developed world. Looking for spinal anomalies is now an integral part of most prenatal ultrasound evaluations worldwide.We don't have many published figures relating to the prenatal diagnosis of spinal anomalies. Here we look into how big this spectrum of disease is; which anomalies can commonly be identified and how often are they identified at a tertiary care center, giving us a better & real idea.

**Methods**: We report the results of 10,000 consecutive prenatal ultrasound scans specifically with respect to the incidence and distribution of spinal anomaly. All scans done at a tertiary center by a single Fetal Medicine expert,over the last 8 years, were studied from a prospective database. Of the data obtained,all patients having spinal bony and cord anomalies were included in the study. Open neural tube defects with acrania were excluded.

**Results**: Out of 10,000 patients studied, 89 were diagnosed with spine and Spinal cord anomalies. Of these 89 patients, 57 had spinal dysraphism.10 had one or more level of hemivertebra.9 patients presented with intra-uterine scoliosis and 22 with kyphosis. Sacral agenesis was present in 7 of these patients.

**Conclusion**: Prenatal diagnosis of spinal anomaly will be a critical cog in the wheel of creating a good perinatal care system in developing nations where logistics and fiscal constraints are overbearing and resource allocation is critical. A team approach comprising of a fetal medicine expert,an obstetrician & Spine surgeon,would help in this.

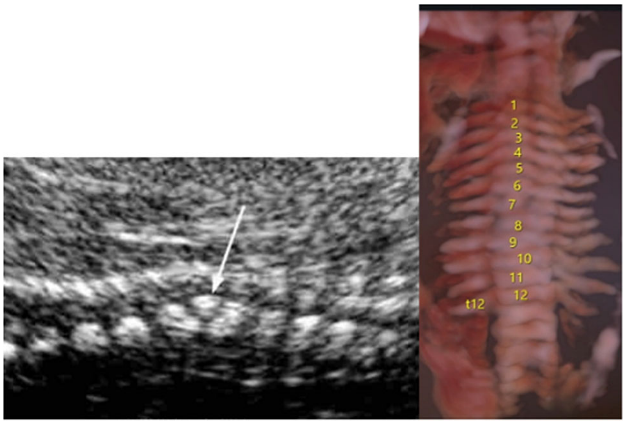

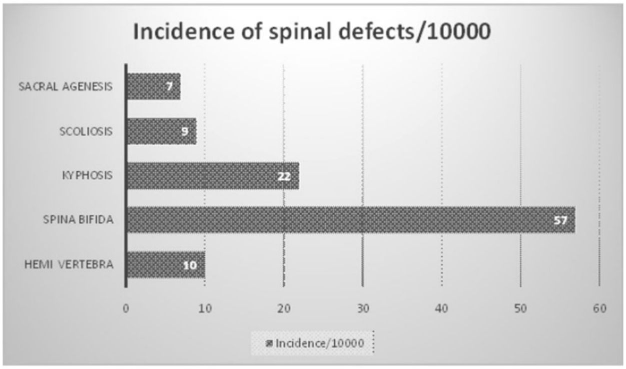

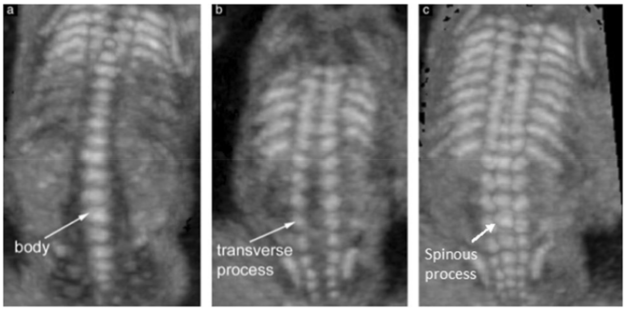

*Author Affiliations and Disclosures: Hriday P. Acharya, Lilavati Hospital and Research Center; Prashant Acharya, Paras Advanced Center for Fetal Medicine; Abhay Nene, Lilavati Hospital*

### Paper # 28.

### Correlation of collagen X biomarker (CXM) with peak height velocity and radiographic measures of growth in idiopathic scoliosis

*Michelle Welborn, MD, Ryan Coghlan, James O. Sanders, MD, Vishwas R. Talwalkar, Amer Samdani, MD, Joseph Stone, MD, Daniel Bouton, MD, William Horton, Susan Sienko*

**Summary**: Current techniques assessing pediatric growth status have minimum > 6 month standard error (SE). Mid-term results indicate CXM is a patient-specific real time measure of growth velocity that is highly reproducible with low SE.

**Hypothesis**: Our Replace is that higher levels of CXM would correlate with rapid longitudinal bone growth while lower CXM levels will indicate growth cessation.

**Introduction**: Assessment of growth in pediatric patients with spinal deformity is critical. Current techniques poorly predict growth and have a large SE. Type X collagen is produced in the growing physis during enchondral ossification. CXM is a breakdown product from type X collagen that can be measured in serum. CXM is thus a direct measure of enchondral ossification.

**Methods**: Q6mo anthropometrics and spine PA biplanar slot scanner images including the hand were assessed for major curve magnitude, Risser score, triradiate cartilage status (TRC), Greulich and Pyle bone age (BA), and Sanders Stage (SS). Longitudinal serial Dried Blood Spots (DBS) were collected on 3 consecutive days Q1-2 months based on SS to obtain CXM levels.

**Results**: 47 patients with idiopathic scoliosis, Cobb = 20 were enrolled. Mean age at enrollment was 11.77 years (range 7.07–16.55 years). CXM levels were assayed in quadruplicate for a total of 3103 samples. CXM results were highly reproducible with an intraassay coefficient of variation of 3%, and interassay of 12%. CXM 3-day average Pearson correlation coefficients was significantly correlated with BA R = 0.860, $p < 0.001$, RS R = 0.607, $p < 0.001$, SS R = 0.716, $p < 0.001$ and with height R = 0.721, $p < 0.001$. No patient with a CXM level < 5 ng/ml had remaining growth.

**Conclusion**: CXM is the first biomarker specific to longitudinal bone growth. Early results indicate it is a patient-specific, real time measure of growth velocity with highly correlated to the established anthropometric and radiographic measures of growth. Furthermore, it appears to be predictive of cessation of growth. It is highly reproducible with a low SE. Longer term follow-up is required to determine the ability of CXM to help guide clinical decision-making.

| | All patients | | Excluding patients <8 yo | |
|---|---|---|---|---|
| | Correlation | P value | Correlation | P value |
| CXM (mean value between visits used to determine height velocity) | 0.721 | P<0.001 | 0.736 | P<0.001 |
| Greulich and Pyle | -0.860 | P<0.001 | -0.588 | P<0.001 |
| Risser Sign | -0.607 | P<0.001 | -0.661 | P<0.001 |
| Sanders Stage | -0.716 | P<0.001 | -0.694 | P<0.001 |

Table 1: Correlation of height velocity between visits and radiographic measures of skeletal maturity

| SS | Gender | # of Samples | Mean CXM level | Range CXM Level | SS | Gender | Number of Samples | Mean CXM level | Range CXM Level |
|---|---|---|---|---|---|---|---|---|---|
| 1 | F | 8 | 24.64 | 15.42-41.27 | 1 | M | 5 | 18.43 | 13.41-25.17 |
| 2 | F | 17 | 28.92 | 14.73-46.54 | 2 | M | | | |
| 3 | F | 16 | 19.36 | 11.2-35.73 | 3 | M | 6 | 20.78 | 13.11-31.36 |
| 4 | F | 8 | 15.44 | 7.84-22.76 | 4 | M | | | |
| 5 | F | 8 | 12.76 | 8.94-19.66 | 5 | M | | | |
| 6 | F | 10 | 9.57 | 6.26-12.62 | 6 | M | 2 | 10.27 | 9.16-11.37 |
| 7 | F | 17 | 8.82 | 3.26-15.78 | 7 | M | 2 | 16.00 | 15.68-16.31 |

Table 2: Mean CXM values in ng/ml by Sanders Stage and Gender

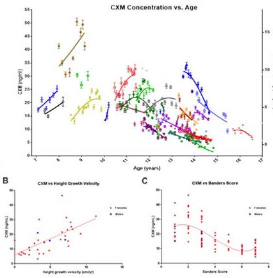

**Figure:** (A) CXM concentration data vs. age shown for each individual in this study, each color representing a different individual. Boys are in shades of blue. Mean CXM values and SE bars are shown for each month, with monthly readings connect by a solid line. (B) CXM concentration data vs. growth velocity. A non-linear line of best fit is shown in red to fit the data (Pearsons r=0.42). (C) CXM concentration data vs. SS for individuals in this study. A third order polynomial non-linear line of best fit is added to the data set in red (Pearsons r= 0.52).

*Author Affiliations and Disclosures: Daniel Bouton, MD, Shriners Hospital for Children – Portland, Medtronic (Consultant); Ryan Coghlan, Shriners Hospital for Children Portland; William Horton, Shriners Hospital for Children Portland; Amer Samdani, MD, Shriners Hospital Outpatient Clinic; James O. Sanders, MD, University of North Carolina at Chapel Hill; Susan Sienko, Shriners Hospital for Children Portland; Joseph Stone, MD, Pediatric Orthopedic Associates; Vishwas R. Talwalkar, Shriners Hospital for Children; Michelle Welborn, MD, Shriners Hospital, Depuy (Speaker's Bureau), K2M (Speaker's Bureau), NuVasive (Speaker's Bureau, Grants/Research)*

### Paper # 29.

### Does anterior vertebral body morphology differ based on age and gender?

*Smitha Mathew, Jaime P. Lazardi, Annalise N. Larson, MD, Todd Milbrandt*

**Summary**: Our study provides accurate anatomic measurements to determine anterior vertebral body screw lengths and trajectories in patients with surgical magnitude AIS curves.

**Hypothesis**: Anterior vertebral body morphology varies and its measurement will help determine anterior screw length and trajectory.

**Introduction**: Anterior spinal instrumentation is growing in use for the treatment of scoliosis. However there are limited anatomic studies in children to guide appropriate screw length, diameter and trajectory. We sought to provide standardized vertebral body morphology values to be used as a guide for the pediatric orthopedic surgery practice.

**Methods**: Retrospective chart review between 2011 and 2019 at a single tertiary center. Scoliosis patients undergoing spinal surgery were imaged with low dose intraop CT prior to instrumentation. Reformatting was performed in plane of deformity to measure anterior vertebral body height at midline, horizontal width and diagonal length ('back-to-front' trajectory). Age at surgery, Risser sign, Sanders score, apical vertebra, proximal thoracic/ thoracic/ lumbar Cobb magnitude were recorded.

**Results**: 833 vertebral bodies between T4-L3 were measured in 102 patients with severe scoliosis. Mean age was 14.2 yrs. Most frequent apical vertebra was T8. Mean Cobb angle was 56°. Vertebral height allowed for 6 mm diameter screw placement up to T4 or T5. Screw length did not vary significantly based on trajectory, but decreased significantly with caudal to cranial progression from 20 to 35 mm. Male gender was associated with longer screw trajectory at all levels, with a mean 3–5 mm increased length at T10 through L3 ($p < 0.01$). Vertebral width also weakly correlated with preop standing height at all levels (Figs. 1,2).

**Conclusion**: Anterior vertebral instrumentation is growing in utilization, but there is limited anatomic data to guide instrumentation systems. This study provides normative data from a representative scoliosis patient population which will help assist screw selection. The 2 trajectories ('back-to-front' length and horizontal) result in similar lengths for screw placement. Screw length was associated with gender and standing height. Further work will assess impact of gender and age on screw length.

**Figure 1**: Mean values of anterior vertebral body horizontal width and diagonal length ('back-to-front' trajectory) increased cranially to caudally

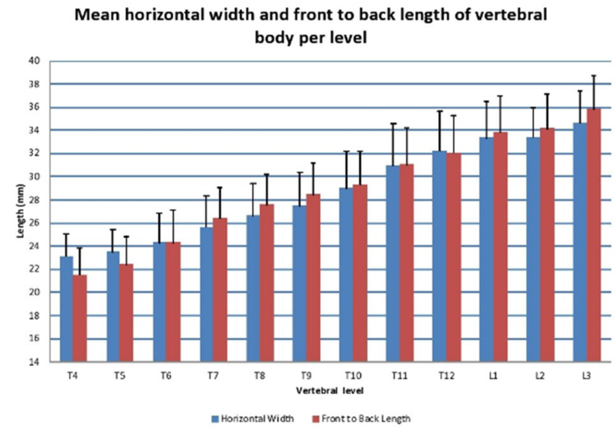

**Figure 2**: Mean vertebral height at midline increased cranially to caudally

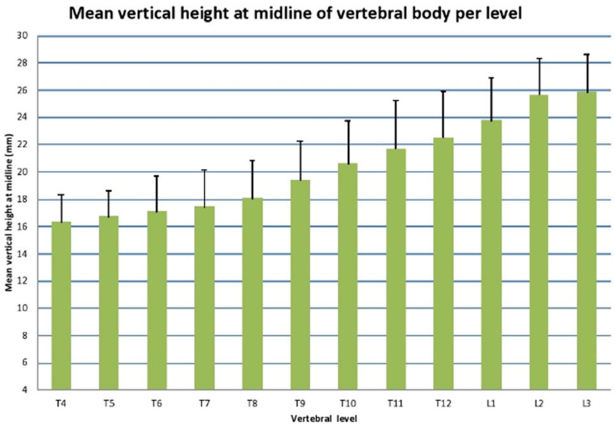

*Author Affiliations and Disclosures: Annalise N. Larson, MD, Mayo Clinic; Jaime P. Lazardi, Mayo Clinic; Smitha Mathew, Mayo Clinic; Todd Milbrandt, Mayo Clinic*

### Paper # 30.

### Novel association between FBN1 variants and congenital scoliosis

*Nan Wu, Jianguo Zhang, Sen Zhao, Zhihong Wu, Shengru Wang, Guixing Qiu*

**Summary**: In this study, we analyzed exome sequencing (ES) data of 615 Chinese CS from the Deciphering Disorders Involving Scoliosis and COmorbidities (DISCO) project. Cosegregation studies for 103

familial CS identified a novel heterozygous nonsense variant, c.2649G > A (p.Trp883Ter) in FBN1. A mutational burden test showed that the deleterious FBN1 variants were significantly enriched in CS subjects.

**Hypothesis**: We hypothesize that FBN1, which is known to be associated with Marfan syndrome, could be also associated with congenital scoliosis.

**Introduction**: Congenital scoliosis (CS) is a form of scoliosis caused by congenital vertebral malformations. Genetic predisposition has been demonstrated in CS. We previously reported that TBX6 loss-of-function causes CS in a compound heterozygous model; however, this model can explain only 10% of CS. Many monogenic and polygenic CS genes remain to be elucidated.

**Methods**: In this study, we analyzed exome sequencing (ES) data of 615 Chinese CS from the Deciphering Disorders Involving Scoliosis and Comorbidities (DISCO) project. The association between FBN1 and CS was then analyzed by extracting FBN1 variants from ES data of 574 sporadic CS and 828 controls.

**Results**: Cosegregation studies for 103 familial CS identified a novel heterozygous nonsense variant, c.2649G > A (p.Trp883Ter) in FBN1. 30 novel variants were identified and prioritized for further analyses. A mutational burden test showed that the deleterious FBN1 variants were significantly enriched in CS subjects (OR? = ?3.9, P? = ?0.03 by Fisher's exact test). One missense variant, c.2613A > C (p.Leu871Phe) was recurrent in two unrelated CS subjects, and in vitro functional experiments for the variant suggest that FBN1 may contribute to CS by upregulating the transforming growth factor-beta (TGF-ß) signaling.

**Conclusion**: Our study expanded the phenotypic spectrum of FBN1 and provided novel insights into the genetic etiology of CS.

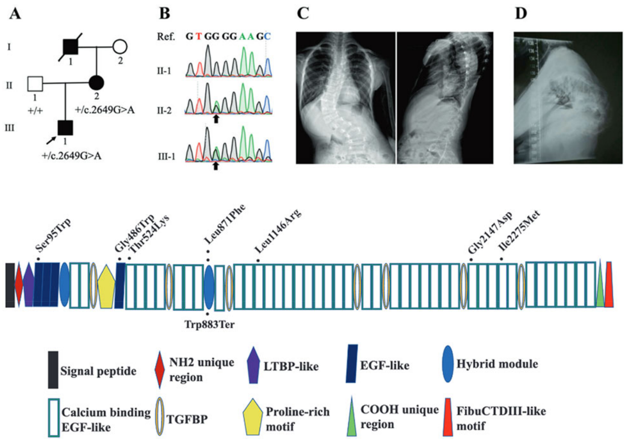

*Author Affiliations and Disclosures: Guixing Qiu, Peking Union Medical College Hospital; Shengru Wang, Peking Union Medical College Hospital; Nan Wu, Peking Union Medical College Hospital; Zhihong Wu, Peking Union Medical College Hospital; Jianguo Zhang, Peking Union Medical College Hospital; Sen Zhao, Peking Union Medical College Hospital*

### Paper # 31.

### Defining an early onset scoliosis "Graduate"—the Pediatric Spine Study Group Experience

*Christina Hardesty, MD, Robert F. Murphy, MD, Jeff J. Pawelek, BS, Michael P. Glotzbecker, MD, Pooria Hosseini, MD, MSc, Charles E. Johnston, MD, John B. Emans, MD, Behrooz A. Akbarnia, MD*

**Summary**: The graduate committee employed the delphi method to create a consensus definition of an early onset scoliosis (EOS) "graduate."

**Hypothesis**: Despite a number of different factors used to describe EOS graduates in the literature, including skeletal maturity, follow-up intervals, thoracic height, the graduate committee felt a consensus definition could be reached.

**Introduction**: Increasingly, patients with early onset scoliosis (EOS) are completing a growth friendly surgical program followed by observation, removal of implants or a definitive spinal fusion. These patients are colloquially referred to as "graduates." A standardized definition of a graduate is needed for research and comparing the outcomes, family counseling, and a better understanding of the population.

**Methods**: A 15-question electronic survey was completed by 39 experienced pediatric spine surgeons to identify factors salient to the definition of a graduate of EOS surgical programs. A Delphi/Nominal group technique session with nine questions was then performed face-to-face with 21 members of the Pediatric Spine Study Group to discuss and refine the definition. A follow-up electronic survey was then distributed to these same 21 members to gain consensus on the final definition.

**Results**

From the initial survey, it was identified that a graduate did not require definitive spinal fusion after a growing program. From the Delphi session, it was determined that skeletal maturity was the most important factor in defining a graduate. A strictly defined minimum length of follow-up was not felt to be a prerequisite for qualification of graduation. After the final electronic version was distributed, > 80% of respondents agreed upon the final definition, thereby achieving consensus.

**Conclusion**: The Pediatric Spine Study Group recommends adoption of the following definition: A "graduate" is a patient who has undergone any surgical program to treat early onset scoliosis, who has reached skeletal maturity and does not have a planned surgical intervention for EOS in the future.

*Author Affiliations and Disclosures: Behrooz A. Akbarnia, MD, University of California, San Diego; John B. Emans, MD, Boston Children's Hospital; Michael P. Glotzbecker, MD, Rainbow Babies & Children's Hospital; Christina Hardesty, MD, Rainbow Babies and Children's Hospital, Medtronic (Consultant), Orthopediatrics (Consultant); Pooria Hosseini, MD, MSc, San Diego Spine Foundation; Charles E. Johnston, MD, Texas Scottish Rite Hospital; Robert F. Murphy, MD, Medical University of South Carolina; Jeff J. Pawelek, BS, Growing Spine Foundation*

### Paper # 32.

### Coronavirus pandemic impact on management and treatment for early onset scoliosis

*Carina Lott, MS, Jason B. Anari, MD, Patrick J. Cahill, MD*

**Summary**: As Coronavirus disease 2019 (COVID-19) spreads worldwide, the overall incidence in early onset scoliosis (EOS) patients and the impact it has on their care remains unknown. We aim to determine the impact of COVID-19 on patient encounters of EOS patients.

**Hypothesis**: Incidence of EOS patients presenting with respiratory illnesses or fever/cough increased during the months of the pandemic compared to the same months in 2019.

**Introduction**: COVID-19 has radically affected the way physicians and institutions manage care for medically complex patients. EOS

patients often have poor pulmonary reserve with various comorbidities, possibly making them high risk for morbidity associated with COVID-19. We aim to examine how COVID-19 has affected patient encounters during peak viral load in our geographic region.

**Methods**: Retrospective review of EOS patients who were admitted inpatient, presented to the ER, or admitted from the ER at our institution during the pandemic (March–June 2020) compared to the same time window pre-pandemic (March–June 2019). Demographics and patient encounters were compared. COVID-19 testing was performed at our institution via in-house developed polymerase chain reaction test with 95% specificity.

**Results**: In March-June 2019, 226 EOS patients were admitted to the hospital while 107 patients were admitted during the pandemic. All patients were screened for COVID-19 during the pandemic; 1 patient tested positive and was asymptomatic. There was a significant decrease in the total number of hospital admissions during the pandemic ($p < 0.001$). Inpatient admissions significantly decreased ($p < 0.001$) and the number of surgical procedures performed was significantly lower during COVID-19 ($p < 0.001$). Presentations to the ER ($p = 0.21$) and admissions from the ER ($p = 0.11$) were similar between both time points. There was no increase in patients with respiratory illness ($p = 1$) and fever/cough ($p = 0.38$) at admission.

**Conclusion**: EOS patient encounters of respiratory illness and fever/cough did not increase due to COVID-19. EOS patients are not contracting COVID-19 at a higher rate than the general public nor are they showing evidence of illness from the virus. With appropriate precautions and prescreening, surgical procedures may be performed safely in EOS patients.

**Table 1.** Demographics and Patient Encounters of Early Onset Scoliosis Patients in Pre-Pandemic and Pandemic Time Periods.

| | Pre-Pandemic (March-June 2019) (n= 226) | Pandemic (March-June 2020) (n=107) | p-value |
|---|---|---|---|
| Gender (M/F) | | | |
| *Male* | 116 | 48 | 0.29 |
| *Female* | 110 | 59 | |
| Lives in patient facility (n) | 8 | 4 | 0.923 |
| Total hospital admissions | | | |
| *March* | 92 | 8 | **<0.001** |
| *April* | 73 | 13 | |
| *May* | 80 | 25 | |
| *June* | 64 | 56 | |
| Inpatient | | | |
| *March* | 59 | 2 | **<0.001** |
| *April* | 46 | 8 | |
| *May* | 47 | 21 | |
| *June* | 39 | 46 | |
| Presentations to ER | | | |
| *March* | 21 | 3 | 0.21 |
| *April* | 13 | 0 | |
| *May* | 23 | 0 | |
| *June* | 9 | 1 | |
| Admissions from ER | | | |
| *March* | 12 | 3 | 0.11 |
| *April* | 14 | 5 | |
| *May* | 10 | 4 | |
| *June* | 16 | 9 | |
| Total number of surgical procedures performed | | | |
| *March* | 53 | 2 | **<0.001** |
| *April* | 43 | 9 | |
| *May* | 37 | 22 | |
| *June* | 34 | 45 | |
| Total number of spine procedures performed | | | |
| *March* | 32 | 0 | **<0.001** |
| *April* | 22 | 3 | |
| *May* | 25 | 16 | |
| *June* | 16 | 28 | |
| Reason for Admission: Respiratory Illness | | | |
| *Inpatient* | 3 | 0 | 1.00 |
| *Presentation to ER* | 11 | 2 | |
| *Admission from ER* | 27 | 5 | |
| Reason for Admission: Fever/Cough | | | |
| *Inpatient* | 1 | 0 | 0.38 |
| *Presentation to ER* | 9 | 1 | |
| *Admission from ER* | 3 | 2 | |

*Author Affiliations and Disclosures: Jason B. Anari, MD, Children's Hospital of Philadelphia; Patrick J. Cahill, MD, The Children's Hospital of Philadelphia; NuVasive Inc (Consultant), Children's Spine Foundation (Grants/Research), Setting Scoliosis Straight Foundation (Grants/Research); Carina Lott, MS, Children's Hospital of Philadelphia*

### Paper # 33.

### Risk of early complication following anterior vertebral body tethering for idiopathic scoliosis

*Abdullah Saad Abdulfattah Abdullah, Stefan Parent, MD PhD, Firoz Miyanji, MD, Kevin Smit, Joshua Murphy, MD, David L. Skaggs, MD, Purnendu Gupta, Michael G. Vitale, MD MPH, Robert Cho, Ron El-Hawary, MD*

**Summary**: 120 skeletally immature patients from multiple centers with 40°–70° idiopathic scoliosis were treated with anterior Vertebral Body Tethering (aVBT) and followed for 1 yr to assess their peri-operative complications and curve correction. This multicenter series illustrated the safety profile for aVBT with an 8.3% complication rate and a 0.8% unplanned return to the operating room (UPROR) at 1 yr postop.

**Hypothesis**: There are low rates of early complication and UPROR following aVBT for idiopathic scoliosis.

**Introduction**: For select patients, aVBT is an alternative to traditional spinal fusion surgery. Samdani has previously published data for a small group treated with aVBT but, to our knowledge, this will be the largest study that has investigated early complications associated with aVBT.

**Methods**: Of 175 patients treated with aVBT, 120 met inclusion criteria. Clinical and radiographic data were analyzed.

**Results**: Mean age was 12.6 yr (8.2–15.7 yr), Risser0-3, thoracic scoliosis 51.2°(40–70°). Immediate postop scoliosis 26.9°(6–53°)* and at 1 yr 22.8°(− 11 to 50°)*. Pre-op T5-T12 kyphosis 16° (− 23 to 52°), 1 yr postop 18° (− 14 to 61°). *$p < 0.05$. All patients underwent thoracoscopic approach with median 4 incisions, EBL 200 ml(20–900 ml), surgical time 215 min (111–472), anesthesia time 303 min(207–480), and hospital stay 4.5 days (2–9). During the in-hospital stay, there was a 0.8% rate of complication and no UPROR: 1pneumothorax requiring reinsertion of chest tube. By 90 days postop, there was a 5% rate of complication. 5 complications developed after discharge:1 CSF leak treated with blood patch injection in the clinic, 2 pleural effusions requiring chest tubes,1 wound infection and 1pneumonia treated with antibiotics. By 1 yr postop, there was a 0.8% rate of UPROR and 8.3% rate of complication. After 90 days, 4 additional complications developed: 2 upper limb paresthesia required outpatient medical management,1 CSF leak required UPROR and 1 curve reversal.

**Conclusion**: This large, multicenter series of aVBT demonstrated an 8.3% complication rate and a 0.8% UPROR rate at 1 yr postop. This early study found higher rates of CSF leaks and overall complications than would be expected for PSFI at 1 yr post-op.

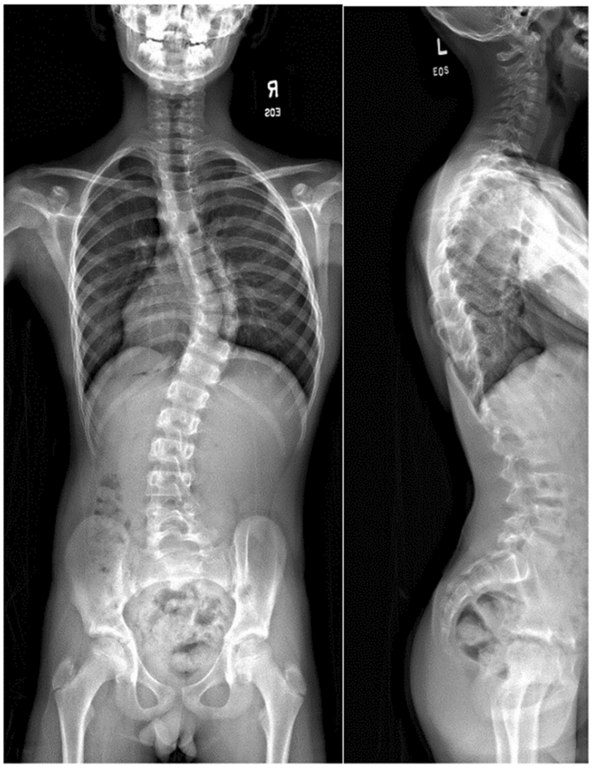

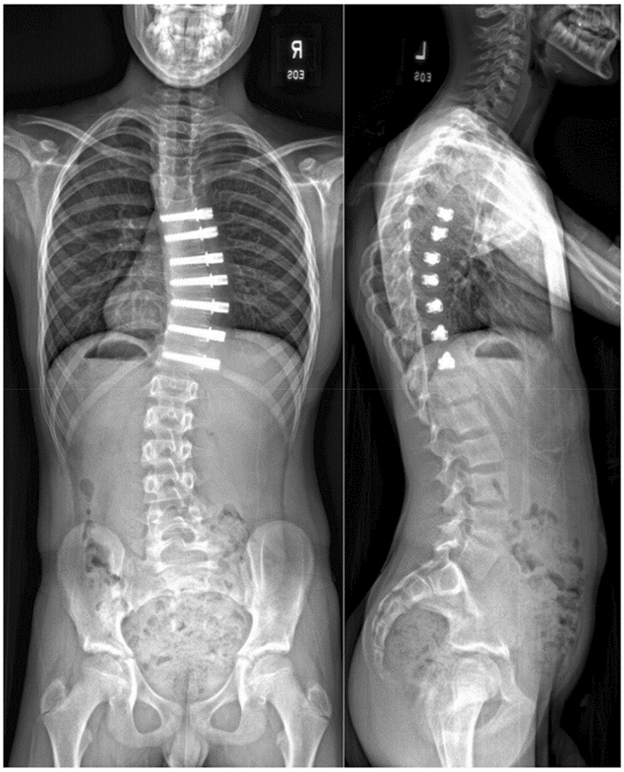

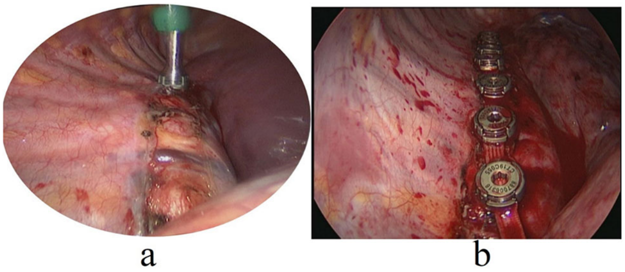

*Author Affiliations and Disclosures: Abdullah Saad Abdulfattah Abdullah, University Health Network; Robert Cho; Ron El-Hawary, MD, IWK Health Centre; Depuy Synthes (Grants/Research), Medtronic (Grants/Research), Orthopediatrics (Consultant, Advisory Board or Panel, Stock/Shareholder (self-managed)); Purnendu Gupta, Shriners Childrens Hospital—Chicago; Firoz Miyanji, MD, BC Children's Hospital; Joshua Murphy, MD, Children's Healthcare of Atlanta; Stefan Parent, MD PhD, CHU Hôpital Ste-Justine; David L. Skaggs, MD, Children's Hospital of Los Angeles; Kevin Smit, 4. Children's Hospital of Eastern Ottawa; Michael G. Vitale, MD MPH, Columbia University Medical Center/Morgan Stanley Children's Hospital.*

### Paper # 34.

### Does aggressive tensioning of the tether cord improve postoperative major cobb correction in scoliosis patients undergoing anterior vertebral body tethering?

*Todd Milbrandt, Smitha Mathew, Abdul Fettah Buyuk, D. Dean Potter, Annalise N. Larson, MD*

**Summary**: In AVBT, aggressive tensioning of the tether cord resulted in 3-month major Cobb correction (45%) similar to preop bending films correction (46%), unlike the 3-mth correction with twist tensioning (38%) which was inferior to the preop bending films correction (54%).

**Hypothesis**: Aggressive tensioning of the cord will produce major Cobb correction similar to preop bending films correction.

**Introduction**: Studies on surgical techniques, including tensioning of the polyethylene-terephthalate (PET) cord, in AVBT are lacking. Tensioning of the cord partially straightens the spine and hence determines postop Cobb angle correction. The amount of tensioning of the cord could help determine the extent of this postop correction.

**Methods**: Patients who underwent AVBT at a single tertiary referral center were reviewed. Patients were categorized as those who underwent twist tensioning of the cord and those with aggressive extra-thoracic tensioning. Preop bending films and 3 month postop EOS radiographs were reviewed and changes in major Cobb recorded. Patients with a 1st erect at 3 month follow up were included in the study.

**Results**: 59 patients met inclusion criteria, with 10 males (16.9%) and 49 females (83.1%). 20(33.9%) patients underwent intraop twist tensioning of cord while 39(66.1%) underwent aggressive tensioning. Mean preop major Cobb correction on bending films in twist tensioning group was 29° while at 3 mth postop it was 21°(p = 0.0006). % correction obtained pre-operatively with bending was 54% while at 3 months postop it was 38% (p = 0.0004). In the aggressive tensioning group, mean preop major Cobb correction on bending films was 24° while at 3 month postop it was 24°(p = 0.69). % correction obtained preoperatively with bending was 46% while at 3 months postop it was 45% (p = 0.71) (Table).

**Conclusion**: Patients who had aggressive tensioning of the cord had major Cobb correction at 3 month follow up similar to those obtained preoperatively on bending films. Twist tensioning of cord had decreased correction. Though AVBT is growing in popularity, many aspects of the surgical technique need to be elucidated. Surgeons need to critically look into their surgical techniques and postop results to help steepen the learning curve.

**Table: Twist tensioning vs Aggressive tensioning**

| | Twist | Aggressive |
|---|---|---|
| Number (n) | 20 | 39 |
| Mean Age at sx (yrs) | 13.5 | 12.9 |
| Gender | Males 5<br>Females 15 | Males 5<br>Females 34 |
| Major Cobb | Thoracic 19<br>Lumbar 1 | Thoracic 34<br>Lumbar 5 |
| Bender type | Standing 6<br>Fulcrum 13<br>Supine 1 | Standing 18<br>Fulcrum 19<br>Supine 2 |
| Pre-op Correction (Bending) | 29° | 24° |
| 3 month Correction | 21° (p = 0.0006) | 24° (p = 0.69) |
| % Pre-op Correction (Bending) | 54% | 46% |
| % 3 month Correction | 38% (p = 0.0004) | 45% (p = 0.71) |

*Author Affiliations and Disclosures: Abdul Fettah Buyuk, Gillette Children's Specialty Healthcare; Annalise N. Larson, MD, Mayo Clinic; Smitha Mathew, Mayo Clinic; Todd Milbrandt, Mayo Clinic; D. Dean Potter, Mayo Clinic*

### Paper # 35.

### Identifying the optimal instrumented level: Prospective case series of 102 patients treated by vertebral body tethering (VBT) with 2-year follow up

*Kenny Y H Kwan, Chris Tang, Stefan Parent, MD PhD, Ron El-Hawary, MD, Firoz Miyanji, MD, Kenneth MC Cheung, MD*

**Summary**: VBT for AIS patients can control curve progression and maintain spinal growth, but the optimal lowest instrumented vertebra (LIV) is not known. We found that choosing a LIV shorter than the lower end vertebra (LEV) had an increased risk of adding-on compared with the LEV or LEV + 1.

**Hypothesis**: We hypothesed there was no difference in selecting LEV or LEV + 1 as the LIV at minimum 2 year follow-up in terms of adding-on in VBT.

**Introduction**: VBT can control curve progression whilst maintaining spinal motion. However, there is no consensus on the optimal choice of LIV.

**Methods**: AIS patients with Lenke 1 curves and a minimum of 2 year follow-up from the PSSG database were reviewed. The patients were stratified into 3 groups according to the selection of LIV: (1) LEV group, (2) LEV + 1 group, and (3) LEV-1 (one level proximal to LEV) group. Primary outcome was the presence of distal adding-on at minimum 2-year follow up radiographs.

**Results**: 102 patients were stratified into 3 groups according to the selection of LIV: (1) End vertebra (EV) group (n = 77), (2) EV + 1 group (n = 11) and (3) EV-1 group (n = 14). EV-1 has significantly higher number of adding-on (42.9%, 6 out of 14) than the other 2 groups (EV: 9.1% (7 out of 77), EV + 1 group: 9.1% (1 out of 11)) in 2-year follow up.

**Conclusion**: Choosing a LIV shorter than the LEV had increased risk of adding-on but not the LEV or LEV + 1 in VBT surgery.

*Author Affiliations and Disclosures: Kenneth MC Cheung, MD, The University of Hong Kong; Ron El-Hawary, MD, IWK Health Centre; Depuy Synthes (Grants/Research), Medtronic (Grants/Research), Orthopediatrics (Consultant, Advisory Board or Panel, Stock/Shareholder (self-managed); Kenny Y H Kwan, University of Hong Kong; Firoz Miyanji, MD, BC Children's Hospital; Stefan Parent, MD PhD, CHU Hôpital Ste-Justine; Chris Tang, Queen Mary Hospital.*

### Paper # 36.

### AVBT vs. Posterior Spinal Fusion for Idiopathic Scoliosis: Results from a Single Tertiary Care Center

*Smitha Mathew, Annalise N. Larson, MD, D. Dean Potter, Todd Milbrandt*

**Summary**: Anterior vertebral body tethering (AVBT) compared to posterior spinal fusion surgery results in decreased major curve correction, operative time, blood loss and length of stay.

**Hypothesis**: AVBT performed in idiopathic scoliosis patients would results in lower blood loss, operative time, and quicker recovery but decreased initial curve correction compared to spinal fusion.

**Introduction**: Anterior vertebral body tethering (AVBT) is a non-fusion technique for the treatment of scoliosis in growing children. However there are limited studies outlining its short- and long-term outcomes. Our center has undertaken an FDA-approved IDE study (FDA IDE G18003, NCT03506334) to prospectively study AVBT using the Dynesys system/Zimmer Biomet Tether system and a thoracoscopic/mini-open lumbar approach.

**Methods**: 30 AVBT patients were matched 1:1 by age, Risser sign and curve magnitude with a cohort of historic AIS fusion patients. 19 AVBT patients were enrolled prior to surgery, and 11 were enrolled after surgery. Hospital record, complications, and preoperative and follow up (3 months, 1 year and 2 year) radiographs were collected. 22 AVBT patients had at least 1 year follow up while 10 had a 2 year follow up.

**Results**: Age, gender, and thoracic curve magnitude were similar between the two groups. Operative time, anesthesia time, blood loss and length of stay was significantly less in the AVBT group compared to the fusion group ($p < 0.0001$) 0.1 AVBT patient had a return to OR due to overcorrection while 2 developed pleural effusion within 30 days of surgery which resolved with thoracentesis. At 3 month follow up, percent correction of the thoracic curve was 45% in the AVBT group compared to 66% in the fusion group ($p = < 0.0001$) which was stable at 2-year follow up with 44% in the AVBT group compared to 66% in the fusion group (p = 0.006) (Table).

**Conclusion**: In this series, length of surgery, blood loss, and hospital stay was decreased in the AVBT group as compared to the fusion patients. Initial major Cobb correction was also decreased in the AVBT group. At 1- and 2-year follow-up, curve correction was less than the fusion group, but stable over time in the AVBT group, while surgical magnitude/recovery was less compared to fusion patients.

**Table: AVBT vs Fusion**

| | AVBT | Fusion | P-values |
|---|---|---|---|
| Number | 30<br>19 – Prospective<br>11 – Retrospective | 30<br>30 – Retrospective | |
| Age at surgery (Years) | 13.1 (SD 1.0) | 13.2 (SD 1.03) | 0.9 |
| Gender | Females (27); Males (3) | Females (27); Males (3) | |
| Thoracic Cobb (°) | 49 (SD 8.9) | 55 (SD 8.9) | |
| Sanders Score | 2 (1 pts), 3 (2 pts), 4 (26 pts), 5 (1 pts) | | |
| Risser (Mean) | 0.7 | 0.8 | 0.7 |
| Blood Loss (ml) | 250 (SD 168) | 927 (SD 518) | <0.0001 |
| Operative Time (hrs) | 4.6 (SD 1.4) | 6.8 (SD 1.0) | <0.0001 |
| Anesthesia Time (hrs) | 7.1 (SD 2.0) | 8.8 (SD 1.1) | <0.0001 |
| Length of stay (days) | 3.3 (SD 0.9) | 5 (SD 1.4) | <0.0001 |
| 3 month % Correction Prox Thoracic | 24% (SD 30) | 45% (SD 63) | <0.0001 |
| 3 month % Correction Thoracic | 45% (SD 16) | 66% (SD 16) | <0.0001 |
| 3 month % Correction Lumbar | 32% (SD 19) | 55% (SD 25) | <0.0001 |
| 1 year % Correction Prox Thoracic | 27% (SD 29) | 55% (SD 23) | 0.0002 |
| 1 year % Correction Thoracic | 53% (SD 19) | 68% (SD 16) | 0.003 |
| 1 year % Correction Lumbar | 34% (SD 26) | 52% (SD 47) | 0.002 |
| 2 year % Correction Prox Thoracic | 36% (SD 23) | 55% (SD 18) | 0.04 |
| 2 year % Correction Thoracic | 44% (SD 22) | 66% (SD 16) | 0.006 |
| 2 year % Correction Lumbar | 21% (SD 22) | 57% (SD 35) | 0.001 |

*Author Affiliations and Disclosures: Annalise N. Larson, MD, Mayo Clinic; Smitha Mathew, Mayo Clinic; Todd Milbrandt, Mayo Clinic; D. Dean Potter, Mayo Clinic*

### Paper # 37.

### Does preoperative and intraoperative imaging for anterior vertebral body tethering predict postoperative correction?

*Abdul Fettah Buyuk, Smitha Mathew, Todd Milbrandt, D. Dean Potter, Annalise N. Larson, MD*

**Summary**: Surgeons should be aware that intraoperative correction during AVBT deteriorates by a mean of 10° on the first erect spine radiographs. Lumbar curves and patients who have atypical scoliosis have even great loss of correction on average. In general, surgeons and families can expect correction on first erect imaging similar to that seen on preoperative bending radiographs.

**Hypothesis**: There would be a loss of correction seen from the intraoperative radiograph to the first erect radiograph and that pre-operative flexibility radiographs would correlate with postoperative first erect curve correction.

**Introduction**: Anterior vertebral body tethering (AVBT) is an emerging approach for idiopathic scoliosis. However, over correction and under correction are common causes of revision surgery, and intraoperative tensioning of the cord is one key component in order to achieve appropriate correction. It is unclear how preoperative curve flexibility relates to intraoperative correction for AVBT and the amount of correction seen on the first erect radiographs.

**Methods**: A total of 57 patients underwent anterior body tethering were included. Preoperative flexibility films and intraoperative radiographs were compared to first erect standing radiographs to see determine if there was a correlation in Cobb angle.

**Results**: Preoperative major Cobb angle measured 52° ± 9°. Major Cobb angle on bending films was 24° ± 9° ($p < 0.001$). Intraoperative imaging showed correction to a mean of 17° ± 9°. Postoperative first erect standing radiographs showed correction to a mean of 27° ± 11° ($p < 0.001$). Mean difference between intraoperative radiograph and first erect radiograph was 10°. Improved correction was seen in the major lumbar curves versus major thoracic curve patterns on preoperative flexibility films (70% vs. 52%, p = 0.014), intraoperatively (94% vs 63%, $p < 0.001$), and at first erect radiograph (68% vs. 44%, p = 0.002). There was a greater correction loss was seen in lumbar curves (mean 14° vs. 10°, p = 0.03).

**Conclusion**: Surgeons should expect major Cobb angle to increase on first erect radiographs compared to intraoperative films, although preoperative bending radiographs provide a reasonable estimate of postoperative correction.

| | TABLE 1. Curve magnitude before surgery, intraoperatively and postoperatively | | | | |
|---|---|---|---|---|---|
| | Preop | Bending | Intraop | 1st Erect | Correction Loss |
| Cobb Angle All (º, n=57) | 52<br>(41, 78) | 24<br>(5, 42) | 17<br>(-7, 38) | 27<br>(3,54) | 10<br>(0,22) |
| % Correction from Preop | Not applicable | 54% | 67% | 48% | 19% |

| TABLE 2. Curve magnitude before surgery, intraoperatively and postoperatively for Thoracic vs. Thoracolumbar/Lumbar | | | | | |
|---|---|---|---|---|---|
| | Preop | Bending | Intraop | 1st Erect | Correction Loss |
| Thoracic (º, n=51) | 52<br>(41,78) | 25<br>(8,42) | 19<br>(-7,38) | 29<br>(3,54) | 10<br>(0-20) |
| Thoracolumbar/Lumbar (º, n=6) | 53<br>(41,60) | 16<br>(5,24) | 3<br>(-2,7) | 17<br>(8,28) | 14<br>(10,22) |
| | | **P=0.02** | **P<0.001** | **P=0.007** | **P=0.03** |
| % Correction from Preop, Thoracic | Not applicable | 52% | 63% | 44% | 19% |
| % Correction from Preop, Lumbar | Not applicable | 70% | 94% | 68% | 26% |
| | | **P=0.014** | **P<0.001** | **P=0.002** | **P=0.03** |

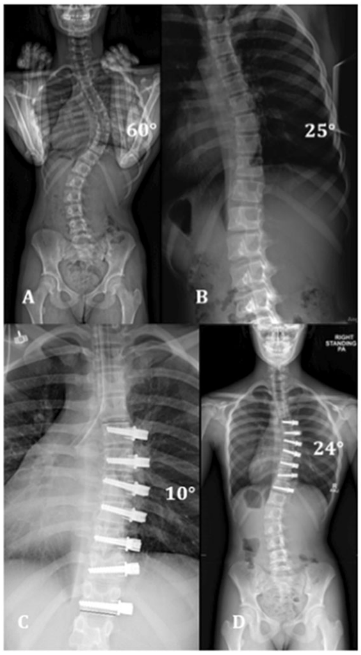

**Figure-3: 14 years old female who underwent AVBT. A) preop standing spine radiograph B) Preop fulcrum radiograph C) intraoperative radiograph D) Postoperative first erect x-ray**

*Author Affiliations and Disclosures: Abdul Fettah Buyuk, Gillette Children's Specialty Healthcare; Annalise N. Larson, MD, Mayo*

*Clinic; Smitha Mathew, Mayo Clinic; Todd Milbrandt, Mayo Clinic; D. Dean Potter, Mayo Clinic*

### Paper # 38.

#### Rate of scoliosis correction correlates with height velocity and slows substantially at 2 years after anterior spinal growth tethering (ASGT) for idiopathic scoliosis

*Yohei Takahashi, Wataru Saito, Burt Yaszay, MD, Carrie Bartley, Tracey Bastrom, Peter O. Newton, MD*

**Summary**: There is a correlation between the rate of scoliosis correction as measured by screw angulation and height velocity for 1–2 years following ASGT for AIS.

**Hypothesis**: Adolescent idiopathic scoliosis (AIS) correction due to ASGT is related to time and growth after implantation.

**Introduction**: Anterior Spinal Growth Tethering (ASGT) has been introduced as a means to progressively correct adolescent idiopathic scoliosis (AIS) due to growth without fusion. The purpose of this study was to evaluate associations between changes in screw angulation and height after ASGT.

**Methods**: Patients with ASGT for AIS between 2012 and 2016 and > 2 yrs of follow-up were retrospectively studied. Screw angulation (more accurate than endplates) of each tethered segment was measured at all postop time points with corresponding patient height measurements. We calculated each segment's screw angulation rate of change (deg/month) and height velocity (cm/month) between each patient's visits (range 3–12 visits/pt) and divided visits into 4 groups by duration after ASGT (< 1 yr, 1–2 yr, 2–3 yr, > 3 yr). Data was excluded after tether breakage, tether or screw removal.

**Results**: We analyzed 23 patients (16F, 7 M) aged 12.2 ± 1.6 years with thoracic AIS of 53 ± 8°. All patients were immature at the time of surgery (Risser 0–1, Sanders 2–3). Mean follow-up was 3.5 ± 1.1 (range 2–5) years. Screw plowing was not identified. In total, we calculated 654 between visit screw angulation changes. The rate of change for each segment's screw angulation after ASGT at < 1 yr, 1–2 yr, 2–3 yr, and > 3 yr was − 0.17, − 0.15, − 0.05, and 0.02 deg/mo [(–) values indicate reduction in scoliosis], and height velocity was 0.63, 0.47, 0.29, and 0.19 cm/mo, respectively. There were significant correlations between changes in screw angulation and increases in height in the group 1–2 years (r = – 0.45, p < 0.001) and 2–3 years (r = − 0.21, p < 0.015) after ASGT.

**Conclusion**: Correction was associated with overall height changes and occurred primarily within 2 years of surgery in this cohort of largely Risser 0 patients. The correction rate was ~ 2 deg/segment/year for the first 2 years after which correction decreased (2-3 yr) and ceased (> 3 yrs). Surgical timing and height velocity are important factors in generating postoperative correction after ASGT.

**The Rates of Change for Intersegmental Screw Angulation (Degrees/Month)**

| Level | N | <1year(N) | 1-2years(N) | 2-3years(N) | 3+years(N) |
|---|---|---|---|---|---|
| T5/T6 | 52 | -0.26 (16) | -0.14 (20) | -0.05 (11) | -0.07 (5) |
| T6/T7 | 107 | -0.23 (28) | -0.18 (35) | -0.09 (23) | 0.03 (21) |
| T7/T8 | 117 | -0.16 (31) | -0.15 (38) | -0.07 (27) | -0.02 (21) |
| T8/T9 | 107 | -0.17 (31) | -0.15 (37) | 0.07 (22) | -0.01 (17) |
| T9/T10 | 113 | -0.07 (31) | -0.16 (38) | -0.04 (24) | 0.07 (20) |
| T10/T11 | 105 | -0.23 (31) | -0.13 (38) | -0.02 (21) | 0.08 (15) |
| T11/T12 | 48 | -0.16 (14) | -0.13 (17) | -0.26 (8) | 0.01 (9) |
| T12/L1 | 5 | -0.09 (3) | -0.17 (2) | - | - |
| Total | 654 | -0.17 (185) | -0.15 (225) | -0.05 (136) | 0.02 (108) |

*Author Affiliations and Disclosures: Carrie Bartley, Rady Children's Hospital; Tracey Bastrom, Rady Children's Hospital; Peter O. Newton, MD, Rady Children's Hospital, San Diego; Wataru Saito, Kitasato University; Yohei Takahashi, Japanese Red Cross Shizuoka Hospital; Burt Yaszay, MD, Rady Children's Hospital—San Diego*

### Paper # 39.

#### Thoracic paravertebral block reduces analgesic requirements in scoliosis patients undergoing anterior vertebral body tethering

*Smitha Mathew, Todd Milbrandt, D. Dean Potter, Annalise N. Larson, MD*

**Summary**: Anterior vertebral body tethering (AVBT) patients who received thoracic paravertebral block (PVB) compared to standard postop pain management had decreased analgesic requirements and length of stay.

**Hypothesis**: Addition of thoracic PVB to our standard postop pain management program would reduce narcotic requirement and length of stay in scoliosis patients undergoing AVBT.

**Introduction**: AVBT is increasingly being performed, yet postop pain management has not been optimized. Multiple chest wall portals and chest tube drainage cause significant postop pain. We sought to determine whether addition of thoracic PVB could provide greater pain relief, reduce analgesic requirement and length of stay and improve patient satisfaction.

**Methods**: Patients who underwent AVBT at a single tertiary center were reviewed. All patients received single intrathecal inj at completion of the procedure in addition to standardized pain management program which included ibuprofen, ketorolac, acetaminophen and opioids (hydromorphone, oxycodone) as needed. 43 patients received a thoracic PVB with lidocaine infusion (left in place for 4–6 days) in addition to standardized care, whereas 24 controls did not have PVB. Length of stay, maximum postop Numeric Pain Intensity Scale (NPIS), and total dose of analgesics/ narcotics administered during hospitalization were noted.

**Results**: 69 patients met inclusion criteria, with 11 males (16%) and 58 females (84%). Postop NPIS > 7 was reported in 9 out of 24 controls (37.5%) and 13 out of 45 patients (28.8%) who received a thoracic PVB in addition to standardized care (p = 0.46). Mean cumulative opioid dose administered was 148.2 oral Morphine Milligram Equivalent(MME) in control group vs.46.6 MME in PVB group (p < 0.0001). Mean analgesic consumption was 13,453 mg in controls vs. 8838 mg in PVB group (p = 0.0001). Mean length of stay in the control group was 3.8 days while in the PVB group it was

3.0 days ($p < 0.001$,Table). There were no complications associated with the PVB.
**Conclusion**: Though postop pain management was adequate in both groups, a thoracic PVB reduced postop analgesic requirement and length of stay, thus improving pain management and potentially reducing risk of opioid dependence in patients undergoing AVBT.

**Table: Use of thoracic paravertebral block decreased length of stay and reduced multimodal analgesic requirement**

| | Patients with usual care (intrathecal hydromorphone injection + acetaminophen/ ibuprofen/ ketorolac and narcotics as needed) (n = 24) | Patients with thoracic paravertebral block in addition to usual care (n = 45) | P value |
|---|---|---|---|
| Age (years) | 13.3 (SD 1.5) | 13.0 (SD 1.4) | 0.6 |
| Mean length of stay (days) | 3.8 (SD 0.92) | 3.0 (0.62) | **<0.001** |
| Maximum Reported Pain Score ≥7/10 | 9 (37.5%) | 13 (28.9%) | 0.47 |
| Mean cumulative dose of opioids (oxycodone/ hydromorphone) during admission (MME) | 148.2 | 46.6 | **<0.001** |
| Mean cumulative dose of acetaminophen/ ibuprofen/ ketorolac during admission (mg) | 13,453 | 8,838 | **0.0001** |

*Author Affiliations and Disclosures: Annalise N. Larson, MD, Mayo Clinic; Smitha Mathew, Mayo Clinic; Todd Milbrandt, Mayo Clinic; D. Dean Potter, Mayo Clinic*

### Paper # 40.

### How does BMI affect post-VBT outcomes?

*Amir S. Mishreky, Stefan Parent, MD PhD, Firoz Miyanji, MD, Joshua Murphy, MD, Pediatric Spine Study Group, Ron El-Hawary, MD*

**Summary**: The outcomes of AIS/JIS patients treated with VBT were compared between underweight, normal weight, and overweight patients. All groups had similar scoliosis correction on 1st erect x-rays and similar risk of complication at 2 yr post-op; however, overweight patients had a risk ratio of 4.7 for progression of scoliosis from 1st post-op erect to 2 year post-op as compared to underweight and normal weight patients.
**Hypothesis**: Overweight patients treated with VBT have decreased initial scoliosis correction, decreased correction over time, and a higher risk of complications as compared to those with normal/low BMI.
**Introduction**: BMI in children has been defined as: Underweight (< 5thile for age), normal (5–85%ile), and overweight (> 85%ile). There has been little research on the effect of BMI on outcomes of VBT.
**Methods**: AIS/JIS patients with VBT with 2 yr f/u from a multicenter EOS database were evaluated pre-op, 1st erect, and 2 yr post-op. ANOVA was used to compare the 3 categories of BMI with significance as per Tukey–Kramer HSD post hoc test. Risk of scoliosis progression was analysed with Mid-P exact test.
**Results**: 121 patients (51 underweight, 58 normal, 12 overweight; mean age 12.5 ± 1.6 yr; BMI 18.8 ± 4.6) were identified. After VBT, scoliosis improved over time (Fig. 1; $p < 0.05$). Comparing underweight, normal, and overweight groups: Mean pre-op age (13 yr, 13 yr, 12 yr), scoliosis (52o, 50o, 52o), pre-op kyphosis (29o, 28o, 33o), peri-op scoliosis correction (44%, 42%, 46%), and complications by 2 yr f/u (23%, 24%, 17%) were similar between groups. There was one broken tether in each of the underweight and normal weight groups. Change in scoliosis % correction from 1st erect to 2 yr post-op was not significantly different between groups (Fig. 2); however, risk ratio for scoliosis progression during this period was 4.74 (1.02- 22.02; p = 0.04) for overweight patients.
**Conclusion**: Overweight patients treated with VBT had similar peri-op scoliosis correction and similar risk of complication as compared to underweight and normal weight patients. As compared to other patients, overweight patients had a risk ratio of 4.7 for progression of scoliosis during the first two years post-op.

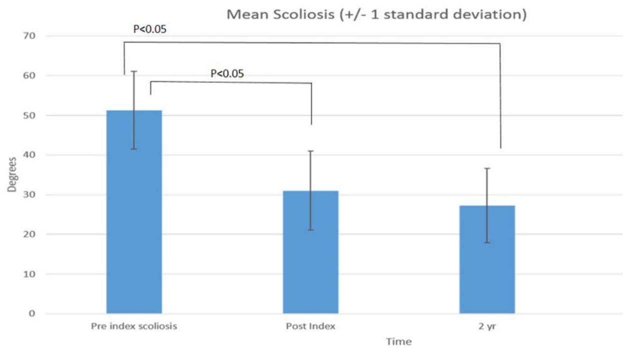

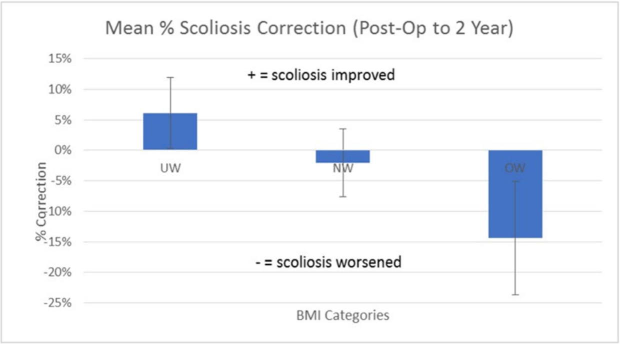

*Author Affiliations and Disclosures: Ron El-Hawary, MD, IWK Health Centre; Depuy Synthes (Grants/Research), Medtronic (Grants/Research), Orthopediatrics (Consultant, Advisory Board or Panel, Stock/Shareholder (self-managed)); Amir S. Mishreky, IWK Health Centre; Firoz Miyanji, MD, BC Children's Hospital; Joshua Murphy, MD, Children's Healthcare of Atlanta; Stefan Parent, MD PhD, CHU Hôpital Ste-Justine; Pediatric Spine Study Group, Children's Spine Foundation; Pediatric Spine Study Group, Pediatric Orthopaedic Society of North America (Grants/Research), FDA (Grants/Research), NuVasive (Grants/Research).*

### Paper # 41.

### Do SRS-22R outcomes improve after anterior vertebral body tethering?

*Annalise N. Larson, MD, Smitha Mathew, D. Dean Potter, Todd Milbrandt*

**Summary**: SRS-22R pain and overall scores improved in scoliosis patients undergoing anterior vertebral body tethering (AVBT) at

1 year post-op as compared to pre-operative and 3-month post-op values.

**Hypothesis**: SRS-22R scores would improve in scoliosis patients following AVBT.

**Introduction**: Success following AVBT has been reported as improvement in spinal curvature. However, patient-reported outcomes following surgery also play an important role in determining success of a procedure.The SRS-22R is a validated patient reported outcome for scoliosis. We sought to determine the change in SRS-22R in patients following AVBT.

**Methods**: Patients who underwent AVBT between 2015 and 2020 at a single tertiary center were reviewed. Patients completed the SRS-22R Patient Questionnaire at their pre-op and follow up (3 months, 1 year and 2 year) visits. Hospital records and maximum postop pain scores were also recorded. Patients with at least 1 year follow up were included in the study.

**Results**: 35 patients met inclusion criteria. There were 7 males (20%) and 28 females (80%). The mean pre-op SRS-22R pain scores was 4.3 while overall SRS-22R score was 4.1 (Table 1). On matched pair analysis, there was significant improvement in SRS-22R pain and overall scores at 1 year follow-up as compared to pre-op scores (4.25 vs 4.56, p = 0.03 and 4.06 vs 4.33, p = < 0.0001)) and 3-month follow up scores (4.05 vs 4.55, p = < 0.0001 and 4.05 vs 4.37, p = < 0.0001). We noted significant improvement in SRS-22R pain scores at 2-year follow-up as compared to the 3-month scores (3.99 vs 4.48, p = 0.03) (Table 2). On multivariate analysis, the most significant predictor of 3-month postop SRS-22R pain scores was preop SRS-22R pain score (R2 0.14 (0.70–0.06), p = 0.02). There was no significant correlation between maximum postop pain scores of > 5 and pre-op SRS-22R pain scores.

**Conclusion**: There was significant improvement in SRS-22R pain and overall scores at 1 year following AVBT. We noted that the most significant predictor of 3-month postop SRS-22R pain scores was pre-op SRS-22R pain score. Our results indicate that disease-specific quality of life measures improve after AVBT. Further work will compare improvements in SRS-22R scores following spinal fusion surgery.

**Table 1**: SRS-22R Pain scores and overall scores

| | Pre op (n=58) | 3 mth (n=43) | 1 year (n=35) | 2 year (n=14) |
|---|---|---|---|---|
| SRS pain score | 4.3 (SD 0.54) | 4.1 (SD 0.62) | 4.54 (SD 0.4) | 4.48 (SD 0.84) |
| SRS overall score | 4.1 (SD 0.37) | 4.12 (SD 0.35) | 4.34 (SD 0.28) | 4.23 (SD 0.43) |

**Table 2: Matched pair analysis: SRS-22R Pain and Overall scores**

| | Pre op | 3mth | 1 yr | 2yr | P value |
|---|---|---|---|---|---|
| SRS Pain pre op vs 3 month (n=28) | 4.23 | 4.03 | | | 0.13 |
| SRS pre op vs 3 month (n=28) | 4.07 | 4.03 | | | 0.62 |
| SRS Pain pre op vs 1 year (n=32) | 4.25 | | 4.56 | | **0.03** |
| SRS pre op vs 1 year (n=32) | 4.06 | | 4.33 | | **<0.0001** |
| SRS Pain pre op vs 2 year (n=13) | 4.13 | | | 4.44 | 0.2 |
| SRS pre op vs 2 year (n=13) | 3.98 | | | 4.2 | 0.12 |
| SRS Pain 3 month vs 1 year (n=30) | | 4.05 | 4.55 | | **<0.0001** |
| SRS 3 month vs 1 year (n=30) | | 4.05 | 4.37 | | **<0.0001** |
| SRS Pain 3 month vs 2 year (n=14) | | 3.99 | | 4.48 | **0.03** |
| SRS 3 month vs 2 year (n=14) | | 3.96 | | 4.23 | 0.1 |
| SRS Pain 1 year vs 2 year (n=14) | | | 4.51 | 4.48 | 1.0 |
| SRS 1 year vs 2 year (n=14) | | | 4.31 | 4.23 | 0.54 |

*Author Affiliations and Disclosures: Annalise N. Larson, MD, Mayo Clinic; Smitha Mathew, Mayo Clinic; Todd Milbrandt, Mayo Clinic; D. Dean Potter, Mayo Clinic*

### Paper # 42.

### Anterior spinal growth tethering leads to asymmetric growth of the periapical vertebrae

*Peter O. Newton, MD, Yohei Takahashi, Yi Yang, Burt Yaszay, MD, Carrie Bartley, Tracey Bastrom, Carlo Munar*

**Summary**: This study provides 3D radiological evidence that anterior spinal growth tethering (ASGT) modulates vertebral and disc growth in patients with progressive scoliosis correction.

**Hypothesis**: ASGT with progressive scoliosis correction is associated with asymmetrical periapical vertebral body growth.

**Introduction**: ASGT utilizes a flexible cord to limit convex spinal growth in immature scoliosis patients.

**Methods**: Patients with ASGT for AIS (all Risser 0 at surgery) between 2012 and 2016, > 2 years of follow-up, and 3D reconstructions based on bi-planar images were retrospectively studied. Patients were divided into two groups: progressive scoliosis correction (PC) or not (NPC). From the 3D reconstructions, averages of the 3 apical vertebral and disc heights (Rt, Lt, Ant, Post) and angular measures were made. The rate of change for each measure (mm/mo, deg/mo) from first erect to 2-year follow-up was compared between groups. Patients were excluded if tether breakage or revision surgery occurred.

**Results**: Fourteen (Risser 0, Sanders 2–3) patients aged 11.4 ± 1.4 years with right thoracic idiopathic scoliosis of 52 ± 9° were included, 7 per group (6F, 1 M/group). Mean follow-up was 3.6 ± 1.1 (range 2–5) years. Although vertebral growth occurred in both groups, the PC group increased the convex, left sided vertebral height at 0.13 mm/mo compared to just 0.05 mm/mo in the NPC group, p = 0.001. Right (tethered side) vertebral growth was not different (PC: 0.07 mm/mo, NPC: 0.05 mm/mo, p = 0.2). The rate of change in coronal vertebral wedging was − 0.11 deg/mo compared to − 0.02 deg/mo for the PC and NPC groups respectively, p = 0.004. The coronal disc angulation also decreased with rates similar to those seen in the vertebrae (PC: − 0.12 deg/mo, NPC: -0.04 deg/mo, p = 0.03) and was associated with loss of right (convex) disc height

(PC: − 0.06 mm/mo) with little effect on the concavity (PC: − 0.01 mm/mo).

**Conclusion**: ASGT in immature patients with thoracic scoliosis can asymmetrically modulate the growth of the periapical vertebrae and discs. Progressive reduction in scoliosis after ASGT was associated with faster concave growth rates in the vertebrae and loss of disc height on the convexity. Given the immaturity of the entire cohort, it remains unclear why some patients responded better than others.

| Average Rate of Change for Each Variable Measurements from the First Erect Post-operative Visit to 2-Year Follow-Up | | | | |
|---|---|---|---|---|
| | PC Group | NPC Group | p-value* | Effect Size |
| Left Vertebral Height (mm/mo) | 0.13 | 0.05 | **0.001** | -0.84 |
| Right Vertebral Height (mm/mo) | 0.07 | 0.05 | 0.165 | -0.39 |
| Anterior Vertebral Height (mm/mo) | 0.09 | 0.03 | **0.007** | -0.70 |
| Posterior Vertebral Height (mm/mo) | 0.12 | 0.06 | **0.007** | -0.70 |
| Vertebral Kyphosis (deg/mo) | 0.03 | 0.02 | 0.620 | -0.15 |
| Vertebral Cobb (deg/mo) | -0.11 | -0.02 | **0.004** | -0.73 |
| Left Disc Height (mm/mo) | -0.01 | -0.01 | 0.535 | -0.19 |
| Right Disc Height (mm/mo) | -0.06 | -0.03 | 0.209 | -0.36 |
| Anterior Disc Height (mm/mo) | -0.04 | -0.01 | 0.805 | -0.09 |
| Posterior Disc Height (mm/mo) | -0.05 | -0.03 | 0.535 | -0.19 |
| Disc Kyphosis (deg/mo) | -0.004 | 0.02 | 0.535 | -0.19 |
| Disc Cobb (deg/mo) | -0.12 | -0.04 | **0.026** | -0.60 |

*p-value was calculated by Mann Whitney U Test.
Bold values represent statistical significance.

*Author Affiliations and Disclosures: Carrie Bartley, Rady Children's Hospital; Tracey Bastrom, Rady Children's Hospital; Carlo Munar, Rady Children's Hospital; Peter O. Newton, MD, Rady Children's Hospital, San Diego; Yohei Takahashi, Japanese Red Cross Shizuoka Hospital; Yi Yang, University of Melbourne; Burt Yaszay, MD, Rady Children's Hospital—San Diego*

### Paper # 43.

#### Cost-utility analysis of anterior vertebral body tethering versus spinal fusion in idiopathic scoliosis from the perspective of the US integrated healthcare delivery system

*David W. Polly, Jr., MD, A. Noelle Larson, Amer Samdani, William Rawlinson, Hannah Brechka, Alex Porteous, William Marsh, Richard Ditto*

**Summary**: Alongside safety and efficacy data, cost-effectiveness (CE) estimates can guide adoption of new healthcare technologies. We conducted an early cost-utility analysis (CUA) to compare anterior vertebral body tethering (VBT) with spinal fusion.

**Hypothesis**: To compare VBT with fusion as a first-choice surgical treatment for skeletally immature patients (aged > 10 years) with idiopathic scoliosis (IS), Cobb angles 30–65°, who failed nonoperative management, from a US integrated healthcare delivery system perspective.

**Introduction**: VBT is a non-fusion, minimally invasive, growth-modulating procedure with early positive clinical outcomes reported in skeletally immature patients with IS (1,2). VBT may offer quality-of-life (QoL) benefits over fusion in allowing patients to retain greater range of motion after surgery.

**Methods**: A Markov state transition model was used to capture a 15-year period following index surgery. Transition probabilities informing revision and subsequent fusion procedures were based on published surgical outcomes and an ongoing VBT observational study (NCT02897453; 1, 2). Utilities were derived from patient-reported outcomes (PROs; SRS-22r mapped to EQ5D) following fusion (3) or VBT. Index and revision procedure costs were included. Probabilistic (PSA) and deterministic sensitivity analyses (DSA) were performed.

**Results**: VBT was associated with higher costs but also higher quality-adjusted life years (QALYs) than fusion (incremental costs: $45,546; incremental QALYs: 0.56). The incremental CE ratio for VBT vs fusion was $80,860/QALY gained (Table). Mean PSA results were similar to the base case, indicating that results were generally robust to uncertainty. Results were most sensitive to variations in utility values in the DSA (Figure).

**Conclusion**: This is the first CUA comparing VBT with fusion in this population and suggests that VBT may be a cost-effective alternative to fusion, given a willingness-to-pay threshold of $100,000/QALY. The results rely on QoL benefits for VBT over fusion; further analyses with longer term PROs for VBT and head-to-head studies are warranted.

**Table**: Cost utility analysis results

| | Base case results | | Mean probabilistic results | |
|---|---|---|---|---|
| | Anterior VBT | Spinal fusion | Anterior VBT | Spinal fusion |
| Total cost ($) | $96,897 | $51,351 | $95,540 | $51,363 |
| Total QALYs | 11.29 | 10.73 | 11.29 | 10.72 |
| Incremental cost ($) | $45,546 | --- | $44,177 | --- |
| Incremental QALYs | 0.56 | --- | 0.57 | --- |
| ICER versus spinal fusion ($/QALY gained) | $80,860 | --- | $77,441 | --- |
| % of PSA simulations cost-effective [a] | --- | --- | 70.8% | 29.2% |

[a] 1,000 Monte Carlo simulations performed at a WTP threshold of $100,000/QALY gained.
Abbreviations: ICER: incremental cost-effectiveness ratio; PSA: probabilistic sensitivity analysis; QALY: qualityadjusted life year; VBT: vertebral body tethering; WTP: willingness-to-pay.

**Figure**: Deterministic sensitivity analysis

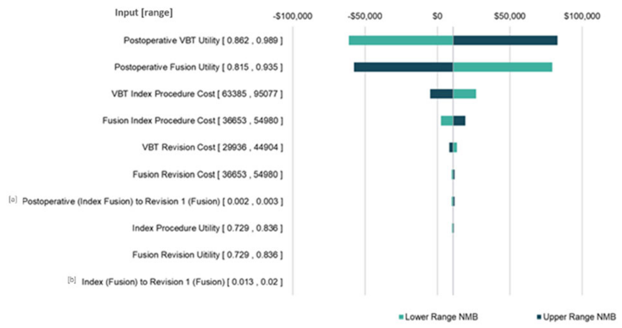

Sensitivity of NMB (based on a WTP threshold of $100,000/QALY) to changes in top 10 model parameters; lowering the parameter indicated in light green, increasing the parameter indicated in dark green. [a] The probability of revision without prior fusion in the last three months; [b] the probability of revision with prior fusion in the last three months. Abbreviations: NMB: net monetary benefit; VBT: vertebral body tethering.

*Author Affiliations and Disclosures: Hannah Brechka, Costello Medical; Hannah Brechka, Costello Medical Consulting Ltd; Richard Ditto, Zimmer Biomet (Employee, Salary, Stock, Stock/Shareholder (self-managed)); A. Noelle Larson, Mayo Clinic; William Marsh, Costello Medical Consulting Ltd; David W. Polly, Jr., MD, University of Minnesota; Alex Porteous, Costello Medical Consulting Ltd; William Rawlinson, Costello Medical Consulting Ltd; Amer Samdani, Shriners Hospitals for Children, Philadelphia*

### Paper # 44.

#### Does the use of growth friendly instrumentation reduce need for positive pressure ventilation in non-neuromuscular scoliosis?

*Klane K. White, MD, Gregory Redding, MD, Brian D. Snyder, MD PhD, David P. Roye, Jr., MD, Oscar Mayer, Patrick J. Cahill, MD, Norman Ramirez-Lluch, MD, Peter F. Sturm, MD*

**Summary**: Existing literature on respiratory support needs after growing rod placement are sparse and have consisted of a large number of neuromuscular patients or those with spondylocostal dysostosis. A review of non-neuromuscular patients with EOS demonstrates an improved response to distraction based growth friendly constructs in patients with a congenital etiology compared to those with syndromic etiologies.

**Hypothesis**: Treatment with distraction based growth friendly spine constructs decreases need for external ventilatory support in non-neuromuscular patients with EOS.

**Introduction**: Restrictive lung disease in early onset scoliosis is associated with hypoventilation and a requirement for positive pressure ventilator support. Previous studies on respiratory support needs after growing rod placement have consisted of neuromuscular (weak) and spondylocostal dysostosis patients. The benefit of growth friendly instrumentation in reducing the use of ventilatory support in non-neuromuscular, early onset scoliosis is not known.

**Methods**: 28 children with EOS on PPV treated with growth friendly constructs with minimum two-year follow-up were identified. Patients with neuromuscular etiology, previous spine surgery, revision surgery or primary TIS were excluded. PPV type, assisted ventilation rating (AVR), BMI, C-EOS etiology, coronal and sagittal Cobb angle, T1-T12 height, T1-S1 height were recorded preoperatively and at $> 2$ years.

**Results**: By C-EOS classification, 13 were congenital/structural, 14 syndromic, 0 idiopathic. AVR improved in 32% (n = 9), was unchanged in 67% and worsened in 7% (combined no improvement n = 19). Improvement varied significantly based on C-EOS etiology (p = 0.003): for patients with improved AVR, 8/9 (89%) were congenital etiology, 1/9 syndromic; for those without improvement 5/19 (28%) were congenital and 13/19 (72%) were syndromic. Patients without improvement demonstrated worsened kyphosis and reduced T1-T12 growth at two years post-operatively.

**Conclusion**: Patients with congenital scoliosis fair better than those with syndromic scoliosis with regard to their need for PPV support. Worsening kyphosis and poor T1-T12 height gain are associated with reduced AVR improvement.

| | Preop Major Cobb (n=23) | Postop Major Cobb (n=16) | Preop Minor Cobb (n=23) | Postop Minor Cobb (n=16) | Preop Kyphosis (n=15) | Postop Kyphosis (n=13) | Preop T1-T12 Height (n=18) | Postop T1-T12 Height (n=15) | Preop T1-S1 Height (n=19) | Postop T1-S1 Height (n=16) |
|---|---|---|---|---|---|---|---|---|---|---|
| **Better (n=9)** | 59.29 | 51.33 | 29 | 21.67 | 42.5 | 33.5 | 10.60 | 16.58 | 17.97 | 28.1 |
| | (27.32) | (9.29) | (18.44) | (14.15) | (24.41) | (9.19) | (2.93) | (3.34) | (4.64) | (4.66) |
| Mean Diff (pre-post) | -7.95 | | -7.33 | | -9 | | 5.97 | | 10.13 | |
| **Same (n=19)** | 53.5 | 55 | 24.63 | 28.85 | 41.36 | 58.73 | 15.07 | 16.42 | 25.13 | 27.56 |
| | (24.05) | (16.13) | (14.61) | (15.49) | (25.01) | (31.87) | (4.43) | (4.08) | (7.02) | (6.02) |
| Mean Diff (pre-post) | 1.5<br>p=0.69 | | 4.22<br>p=0.45 | | 17.37<br>**p=0.07*** | | 1.35<br>p=0.32 | | 2.43<br>**p=0.03*** | |

Demographics

| | Better (n=9) | Same (n=19) | P value |
|---|---|---|---|
| Age | 3.19 (3.5) | 4.08 (2.46) | 0.2603 |
| Gender (Female) | 5 (56%) | 13 (68%) | 0.507 |
| Preop BMI | 19.13 (3.42) | 18.49 (6.07) | 0.3826 |
| Etiology<br>• Congenital<br>• Syndromic | <br>8 (89%)<br>1 (11%) | <br>5 (28%)<br>13 (72%) | <br>**0.003*** |

*Author Affiliations and Disclosures: Patrick J. Cahill, MD, The Children's Hospital of Philadelphia; NuVasive Inc (Consultant), Children's Spine Foundation (Grants/Research), Setting Scoliosis Straight Foundation (Grants/Research); Oscar Mayer, Children's Hospital of Philadelphia (CHP); Norman Ramirez-Lluch, MD, Hospital de la Concepcion—San German; Gregory Redding, MD, Seattle Children's Hospital; David P. Roye, Jr., MD, Morgan Stanley Children's Hospital; Brian D. Snyder, MD PhD, Boston Children's Hospital; Peter F. Sturm, MD, Cincinnati Children's Hospital Medical Center; Klane K. White, MD, Seattle Children's Hospital, Biomarin (Grants/Research, Consultant), Ascendis (Grants/Research)*

### Paper # 45.

#### Longitudinal assessment of pulmonary hypertension and cardiac function by echocardiogram in early onset scoliosis

*Carina Lott, MS, Catherine Qiu, Patrick J. Cahill, MD, Catherine Avitabile, Jason B. Anari, MD*

**Summary**: Early onset scoliosis (EOS) patients can have pulmonary hypertension (PH) and cardiac ventricular dysfunction due to respiratory insufficiency, but the effect of surgery on heart function is unknown. We aim to assess changes in PH, right ventricular (RV) and left ventricular (LV) function in patients undergoing insertion of rib-based construct with echocardiograms. After surgery, RV systolic function significantly improved and LV systolic function preserved showing likely improvement in PH.

**Hypothesis**: RV and LV function in EOS patients will improve after insertion of rib-based construct.

**Introduction**: Cardiopulmonary disease is a problem surgeons face when treating EOS patients as many may have PH and risk RV dysfunction due to altered chest mechanics. We aimed to understand the prevalence of PH in patients undergoing rib-based instrumentation and assess the impact of surgery on cardiac function.

**Methods**: We reviewed serial echocardiograms (echo) in EOS patients undergoing rib-based surgery from 2008 to 2019. Patients with a pre-op, post-op, and recent outpatient echo with = 2 yr f/u were included. PH was defined by at least one echo criteria: RV pressure $> 1/2$ systemic blood pressure estimated from tricuspid regurgitant jet velocity, bidirectional or right-to-left PDA shunt, flattened or bowing ventricular septum at end-systole, and/or treatment with PH medications at initial presentation. RV systolic function was evaluated by tricuspid annular plane systolic excursion (TAPSE) and Z-scores for age were generated. LV function was evaluated by M-mode shortening fraction. One-way ANOVA was performed for statistical analysis.

**Results**: 11 patients met inclusion criteria; 8 had a pre-op diagnosis of PH. PH was most commonly diagnosed by a flattened ventricular septum at end-systole. There was a trend toward improvement in echo evidence of PH (p = 0.09) and TAPSE improved, reflecting significant improvement in RV systolic function (p = 0.05). LV shortening fraction remained stable after surgery (p = 0.18). 8 patients were treated with pulmonary vasodilators during the study period, most commonly sildenafil.

**Conclusion**: Many patients showed improvement in PH after rib-based surgery as measured on echo, showing the potential benefit of surgery in EOS.

**Table 1.** Patient characteristics and serial echocardiographic data (n=11).

| Variables | Pre-op Echo | Post-op Echo | Most Recent Echo | P Value |
|---|---|---|---|---|
| Age at echo (years) | 4.61±5.38 | 5.45±5.42 | 9.16±5.97 | |
| Interval from surgery (days) | 127±88.80 | 177±133 | 1532±1341 | |
| Height (cm) | 85.5±34.30 | 97.5±28.60 | 117.4±27.40 | |
| Weight (kg) | 14.6±9.13 | 17.1±8.75 | 24.4±10.30 | |
| Presence of PH | 8 (73%) | 3 (27%) | 5 (45%) | |
| By echocardiographic criteria | 7 | 3 | 5 | **0.03** |
| On PH medications at initial presentation | 4 | n/a | n/a | |
| Echocardiographic criteria of PH | | | | |
| RV pressure > ½ systemic blood pressure | 1 | 1 | 0 | 0.09 |
| Flattened or bowing ventricular septum at end-systole | 7 | 3 | 5 | |
| Bidirectional or right to left PDA shunt | 1 | 0 | 0 | |
| TAPSE, cm | 1.35±0.37 | 1.44±0.30 | 1.70±0.37 | **0.05** |
| TAPSE Z-score | -1.66±1.92 | -2.41±2.58 | -1.73±2.23 | 0.63 |
| Left Ventricle Internal Diameter in Diastole (LVIDd) Z-Score | -0.65±2.45 | -1.23±1.59 | -0.90±1.72 | 0.65 |
| Left Ventricle Internal Diameter in Systole (LVIDs) Z-Score | -0.86±2.26 | -1.36±1.37 | -0.70±1.71 | 0.50 |
| Left Ventricle Shortening Fraction (LVSF) | 39.3±4.46 | 39.36±4.03 | 36.7±5.01 | 0.18 |

**Note:**
PH: pulmonary hypertension
RV: right ventricle
PDA: patent ductus arteriosus
TAPSE: tricuspid annular plane systolic excursion

*Author Affiliations and Disclosures: Jason B. Anari, MD, Children's Hospital of Philadelphia; Catherine Avitabile, Children's Hospital of Philadelphia; Patrick J. Cahill, MD, The Children's Hospital of Philadelphia; NuVasive Inc (Consultant), Children's Spine Foundation (Grants/Research), Setting Scoliosis Straight Foundation (Grants/Research); Carina Lott, MS, Children's Hospital of Philadelphia; Catherine Qiu, The Children's Hospital of Philadelphia*

### Paper # 46.

### Radiographic outcomes and pulmonary function from early surgery in non-neuromuscular scoliosis: follow-up to a landmark study

*Pablo Eamara, Charles E. Johnston, MD, Anna M. McClung, Researcher, David C. Thornberg, Dong-Phuong Tran, MS, ChanHee Jo*

**Summary**: We reviewed 28 EOS patients with index surgery < 9 yr, minimum 5 yr postop follow-up.18/28 had = 50% pred PFT results, 17 had = 18 cm T1-12 length. There was no difference in % pred PFTs based on T1-12 length being over /under 18 cm. However, patients with = 50% pred PFTs had smaller curves and longer T1-12 preop and at follow-up. Initial surgery below/above age 5 yr made no difference in curve magnitude or % pred PFTs at follow-up.

**Hypothesis**: Early surgery (< 5 yrs) does not necessarily impair PFTs if correction can also be accomplished.

**Introduction**: Karol's oft-quoted landmark study advised avoidance of early fusion, which produced PFTs < 50% pred when final T1-12 length = 18 cm, although correction and Cobb values were not reported. We compared PFTs depending on age at initial surgery (below/above 5 yr), T1-12 length (O/U 18 cm) and curve correction after 5 yr minimum f/u.

**Methods**: Patients having initial surgery < 9 yr 2004–14 were reviewed. N-M, skeletal dysplasia and pre-existing pulmonary dx's excluded. Age @ surgery, T1-12 length, Cobb angle and PFTs were compared.

**Results**: There were 15 congenital, 11 syndromic, 2 EOIS diagnoses, 22 having growth-friendly index surgery (mean age 4.8 yr) while 6 had definitive fusion (mean 5.1). Index surgery before/after 5 yrs had no effect on final curve size or % pred PFTs. 18/28 (64%) had PFTs = 50% while 17/28 (61%) had T1-12 = 18 cm@ f/u. Patients with T1-12 = 18 cm had actual lung volumes > those with T1-12 < 18 cm but % PFTs were no different (FVC pred 54 vs 53%, FEV1 pred 54 vs 49% (p = ns). Patients with = 50% pred PFTs had smaller initial & f/u curves—66 and 44, compared to 83 and 60O (p = 0.05, 0.006) for < 50%pred group; and longer T1-12 at index and f/u – 14.7 and 19.8, compared to 11.7 and 16.8 cm (p = 0.03,0.05) for < 50%pred.

**Conclusion**: O/U 18 cm for T1-12 length at 5 y min follow-up post index surgery showed no difference in % pred PFTs, due to effective curve correction (39%) in patients < 18 cm. Those with PFTs > 50% pred had smaller curves and greater T1-12 length index and final compared to < 50% PFT patients. Index surgery above/below 5 yrs made no difference in final Cobb or % pred PFT.

| Patients at Latest Follow-Up, minimum 5 years after Index Surgery [N=28] | Under or Over 50% Predicted | | | Under or Over 18 cm T1-T12 Height | | | Under or Over 5 yo. at Index Surgery | | |
|---|---|---|---|---|---|---|---|---|---|
| | < 50% | ≥ 50% | p-value | < 18 cm | ≥ 18 cm | p-value | < 5 yo | ≥ 5 yo | p-value |
| Index Cobb (Degrees) | 82.9° | 66.3° | 0.050 | 88° | 62° | 0.001 | 79.7° | 65.2° | 0.175 |
| Index T1-T12 Height (mm) | 11.7 | 14.7 | 0.034 | 10.6 | 15.6 | 0.000 | 12.4 | 15.6 | 0.018 |
| Index T6 Chest Depth (mm) | 68.8 | 70.1 | 0.797 | 67.6 | 71.0 | 0.483 | 69.9 | 69.2 | 0.890 |
| Age at Index Surgery (yrs.) | 4.37 | 5.14 | 0.344 | 3.59 | 5.69 | 0.005 | 3.52 | 6.93 | 0.000 |
| Time between Surgery & Follow-Up (yrs.) | 9.4 | 8.3 | 0.309 | 9.1 | 8.5 | 0.563 | 9.3 | 7.7 | 0.106 |
| Age at Follow-Up (yrs.) | 13.8 | 13.5 | 0.785 | 12.7 | 14.2 | 0.127 | 12.9 | 14.7 | 0.066 |
| Final F/U Major Cobb (Degrees) | 60.1° | 44.1 | 0.006 | 53.4° | 47.5° | 0.332 | 51.0° | 47.9° | 0.613 |
| Final F/U T1-T12 Height (cm) | 16.8 | 19.8 | 0.052 | 14.59 | 21.35 | 0.000 | 17.55 | 20.47 | 0.051 |
| Final F/U T6 Chest Depth (mm) | 88.6 | 86.6 | 0.799 | 88.3 | 86.7 | 0.851 | 91.1 | 81.5 | 0.209 |
| FVC Actual (L) Final F/U | 0.97 | 1.91 | 0.000 | 1.19 | 1.83 | 0.011 | 1.45 | 1.79 | 0.206 |
| FEV1 Actual (L) Final F/U | 0.85 | 1.56 | 0.000 | 1.08 | 1.46 | 0.062 | 1.23 | 1.43 | 0.317 |
| FVC % Predicted Final F/U | 32.9 % | 64.9% | 0.000 | 53.4 % | 53.5 % | 0.925 | 52.2 % | 55.4 % | 0.693 |
| FEV1 % Predicted Final F/U | 32.9 % | 61.2% | 0.000 | 54.2 % | 49.1 % | 0.548 | 50.6 % | 51.8 % | 0.890 |
| △ Cobb (Degrees) [Post – Pre] | -22.7° | -22.2° | 0.952 | -34.6° | -14.53° | 0.011 | -25.7° | -17.3° | 0.314 |
| △ T1-T12 Height (cm) [Post – Pre] | 50.6 | 50.9 | 0.977 | 3.99 | 5.79 | 0.091 | 5.19 | 4.91 | 0.802 |
| △ Chest Depth (mm) [Post – Pre] | 19.8 | 16.5 | 0.606 | 2.07 | 1.57 | 0.423 | 2.11 | 1.23 | 0.314 |

*Author Affiliations and Disclosures: Pablo Eamara, FLENI Foundation; ChanHee Jo, Texas Scottish Rite Hospital for Children; Charles E. Johnston, MD, Texas Scottish Rite Hospital; Anna M. McClung, Researcher, Texas Scottish Rite Hospital for Children; David C. Thornberg, Texas Scottish Rite Hospital for Children; Dong-Phuong Tran, MS, Texas Scottish Rite Hospital*

### Paper # 47.

### Radiation exposure during the treatment of spinal deformities: Benchmarking the radiation exposure during the treatment journey

*Jwalant S. Mehta, FRCS Orth, James Kho, Kirsten Hodgson, Lu Yiping, Upasana Topiwala, Ravindra Thimmiah, Vijay Sawlani, Rajesh Botchu*

**Summary**: The treating clinicians should be cognizant of the levels of radiation doses that the patients are exposed to. The doses are 2–3 times higher when surgery is undertaken. Clinicians should look at safer alternatives in the outpatients, intra-operative and post-operative settings.

**Hypothesis**: To benchmark the radiation dose during the patients journey during treatment for a spinal deformity.

**Introduction**: The perceived risks of radiation poses a challenge to the treating clinicians. Monitoring curve progression requires radiation exposure. There are no studies or guidelines that document the radiation doses during routine monitoring and when undertaking surgical corrections, of children and adults. Our objective is to document the radiation exposure doses in various clinical settings for spinal deformity patients.

**Methods**: The radiation dose database identified 3765 patients under the age of 18 years (mean age 12.19 years) treated in our unit between January 1, 2008 and 31 December 2016. The patients were dichotomised into surgical (789 patients) and non-surgical (2976 patients). We documented the number and doses of ionising radiation imaging events (radiographs, CT scans or intra-operative fluoroscopy) for each patient. All the doses for plain radiographs, CT scans and intra-operative fluoroscopy were unified to a single unit effective dose by the medical physicist (mSv).

**Results**: There were more ionising radiation-based imaging events and higher radiation dose exposures in the surgical v non-surgical groups ($p < 0.001$*). The difference in effective dose for under 18 years between the surgical and non surgical groups is statistically significant with the surgical group being significantly higher. This leads to a higher estimated risk of cancer induction for the surgical group (1:222 surgical v 1:1418 non-surgical). In all cases the effective dose was significantly higher than the natural background radiation exposure.

**Conslusion**: Spinal deformity treatments are radiation heavy. The dose exposure is 2 to 3 times higher when surgical treatment is undertaken. Clinicians should be cognisant of this and appraise their practices to be able to reduce the radiation dose when possible.

| | Non surgical | Surgical | P values |
|---|---|---|---|
| **<=18 years** | | | |
| Number of X ray event | 3.17±2.17 | 8.57±4.18 | <0.001* |
| Number of CT events | 0.06±0.27 | 0.38±0.68 | <0.001* |
| Total X ray + fluoro dose (cGycm²) | 324.92±1603.79 | 865.53±773.36 | <0.001* |
| Total CT dose (mGycm) | 33.77±222.27 | 276.92±570.25 | <0.001* |
| | | | |
| **0-9 years** | | | |
| Number | 695 | 146 | |
| Mean age | 4.99±2.91 | 5.54±2.64 | |
| Average Dose (mSv) | 51.90±245.01 | 684.81±1477.8 | <0.001 |
| Average risk (1 in...) | 1419±2423 | 223±373 | |
| Comparison with background Average (years) | 22.57±107.00 | 297.74±642.52 | |
| | | | |
| **10-18 years** | | | |
| Number | 2281 | 640 | |
| Mean age | 14.32±2.15 | 13.95±2.10 | |
| Average Dose (mSv) | 228.25±1049.05 | 1362.31±2442.64 | <0.001 |
| Average risk (1 in...) | 412±559 | 84±143 | |
| Comparison with background Average (years) | 99±456 | 592.31±1062.02 | |

**Figure 1:** Regression analysis of age v total dose

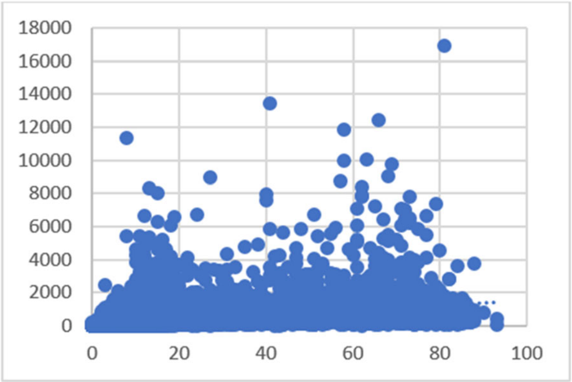

**Figure 2**: Regression analysis of the total dose v number of radiograph events

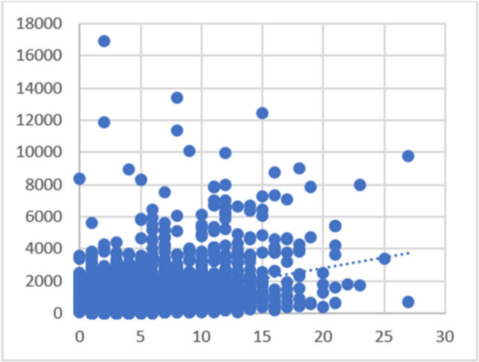

*Author Affiliations and Disclosures: Rajesh Botchu, Royal Orthopaedic Hospital; Kirsten Hodgson, University Hospitals Birmingham NHS Foundation Trust; James Kho, Royal Orthopaedic Hospital; Jwalant S. Mehta, FRCS Orth, Royal Orthopaedic Hospital and Birmingham Children's Hospital, Stryker Spine / K2M (Consultant), DePuy Synthes (Grants/Research), NuVasive (Grants/Research, Speaker's Bureau, Stock/Shareholder (self-managed)); Vijay Sawlani, University of Birmingham Medical School; Ravindra Thimmiah, Royal Orthopaedic Hospital; Upasana Topiwala, University of Birmingham Medical School; Lu Yiping, Huashan Hospital*

### Paper # 48.

### Looking under the hood: factors that drive successful study group participation and publications in pediatric spine programs

*Sonya B. Levine, BA, Bradley T. Hammoor, MS, Abby Morris, MHA, Sushrut Arora, MPH, Afrain Z. Boby, MS, Hiroko Matsumoto, PhD, Michael W. Fields, BS, Matthew Oetgen, MD MBA, Tricia St. Hilaire, MPH, Brandon A. Ramo, MD, Douglas L. Brockmeyer, MD, Richard Anderson, MD, John T. Smith, MD, Michael G. Vitale, MD MPH, David L. Skaggs, MD, Pediatric Spine Study Group.*

**Summary**: This study compares survey results from members of an EOS registry with registry generated performance scores and publication outcomes. Full-time research staff and dedicated spine focus were highly associated with increased performance scores and publication volume.

**Hypothesis**: Centers that perform well with regard to EOS registry participation share certain features, providing guidance to centers that wish to improve participation.

**Introduction**: Multicenter clinical research is critical in a constantly evolving landscape, particularly in early onset scoliosis (EOS). There is variability among centers with regard to the "quality of participation" in research, as well as amount of publication on EOS. The purpose of this study is to examine factors associated with "high-performing" centers.

**Methods**: 21 academic medical centers participating in an EOS registry were assessed. Factors examined included research personnel, spine research focus and regular participation of faculty in research, collected through a survey. Outcomes included the quality of participation in the study group, derived from quarterly site reports generated by the registry and average annual publication volume of each center using PubMed searches.

**Results**: Centers with full-time spine research staff had higher average quality of participation scores (90 vs 60, p = 0.026) as did centers with dedicated spine research meetings (90 vs 70, p = 0.074) than those that lacked these features. Additionally, centers with higher average publication volumes were more likely to have full-time research staff (8.0 vs 3.5, p = 0.115), a research team focused on spine (8.9 vs 4.8, 0.067) and a dedicated physician assistant (PA) or nurse with > 95% focus on spine (9.0 vs 5.4, p = 0.107).

**Conclusion**: The single most important and significant factor in quality of participation in the registry was a center having full-time research staff. Factors associated with publication volume was a research team focused primarily on spine and a spine dedicated PA or nurse. If institutions want to improve spine research, we recommend investing in these factors.

*Author Affiliations and Disclosures: Richard Anderson, MD, Neurosurgeons of New Jersey; Sushrut Arora, MPH, New York–Presbyterian Hospital; Afrain Z. Boby, MS, Columbia University Medical Center; Douglas L. Brockmeyer, MD, University of Utah; Michael W. Fields, BS, Columbia University; Bradley T. Hammoor, MS, Columbia University Medical Center; Sonya B. Levine, BA, Columbia University Medical Center; Hiroko Matsumoto, PhD, Columbia University; Abby Morris, MHA, Columbia University Medical Center; Matthew Oetgen, MD MBA, Children's National Health System; Brandon A. Ramo, MD, Texas Scottish Rite Hospital for Children; David L. Skaggs, MD, Children's Hospital of Los Angeles; John T. Smith, MD, University of Utah; Tricia St. Hilaire, MPH, Children's Spine Foundation; Pediatric Spine Study Group, Children's Spine Foundation; Michael G. Vitale, MD MPH, Columbia University Medical Center/Morgan Stanley Children's Hospital; Pediatric Spine Study Group, Pediatric Orthopaedic Society of North America (Grants/Research), FDA (Grants/Research), NuVasive (Grants/Research)*

**Open Access** This article is licensed under a Creative Commons Attribution 4.0 International License, which permits use, sharing, adaptation, distribution and reproduction in any medium or format, as long as you give appropriate credit to the original author(s) and the source, provide a link to the Creative Commons licence, and indicate if changes were made. The images or other third party material in this article are included in the article's Creative Commons licence, unless indicated otherwise in a credit line to the material. If material is not included in the article's Creative Commons licence and your intended use is not permitted by statutory regulation or exceeds the permitted use, you will need to obtain permission directly from the copyright holder. To view a copy of this licence, visit http://creativecommons.org/licenses/by/4.0/.

Publisher's Note

Springer Nature remains neutral with regard to jurisdictional claims in published maps and institutional affiliations.

